# Translation in astrocyte distal processes sets molecular heterogeneity at the gliovascular interface

**DOI:** 10.1038/celldisc.2017.5

**Published:** 2017-03-28

**Authors:** Anne- Cécile Boulay, Bruno Saubaméa, Nicolas Adam, Stéphanie Chasseigneaux, Noémie Mazaré, Alice Gilbert, Mathieu Bahin, Leïla Bastianelli, Corinne Blugeon, Sandrine Perrin, Juliette Pouch, Bertrand Ducos, Stéphane Le Crom, Auguste Genovesio, Fabrice Chrétien, Xavier Declèves, Jean-Louis Laplanche, Martine Cohen-Salmon

**Affiliations:** 1 Neuroglial Interactions in Cerebral Physiopathology/Collège de France, Center for Interdisciplinary Research in Biology (CIRB)/Centre National de la Recherche Scientifique CNRS, UMR 7241/Institut National de la Santé et de la Recherche Médicale INSERM, U1050/University Pierre et Marie Curie UPMC, ED, No. 158/MEMOLIFE Laboratory of Excellence and PSL Research University, Paris, France; 2 Variabilité des réponses aux psychotropes, INSERM U1144/Faculté de Pharmacie de Paris/Université Paris Descartes/Université Paris Diderot/Université Sorbonne Paris Cité, Paris, France; 3 Cellular and Molecular Imaging Facility, INSERM US25/CNRS UMS 3612/Faculté de Pharmacie de Paris/Université Paris Descartes/Université Sorbonne Paris Cité, Paris, France; 4 Institut Pasteur, Human Histopathology and Animal Models, Paris, France; 5 Université Paris Descartes–Sorbonne Paris Cité, Paris, France; 6 Service de Neuropathologie, Centre Hospitalier Sainte-Anne, Paris, France; 7 Plateforme Bioinformatique, Ecole Normale Supérieure/Institut de Biologie de l’ENS (IBENS) /INSERM, U1024/CNRS, UMR 8197, Paris, France; 8 Plateforme Génomique, Ecole Normale Supérieure/PSL Research University/IBENS/INSERM/CNRS, Paris, France; 9 Plateforme de qPCR à Haut Débit, Ecole Normale Supérieure/IBENS, Paris, France; 10 Laboratoire de Physique Statistique, ENS/CNRS UMR 8550/PSL Research University/Université Paris Diderot Sorbonne Paris-Cité/Sorbonne Universités UPMC, Paris, France; 11 Evolution Paris Seine, Institut de Biologie Paris-Seine (IBPS)/Sorbonne Universités, UPMC/CNRS UMR 7138, Paris, France

**Keywords:** astrocyte, gliovascular unit, endfeet, translating ribosome immunoprecipitation, local translation, mRNAs localization

## Abstract

Astrocytes send out long processes that are terminated by endfeet at the vascular surface and regulate vascular functions as well as homeostasis at the vascular interface. To date, the astroglial mechanisms underlying these functions have been poorly addressed. Here we demonstrate that a subset of messenger RNAs is distributed in astrocyte endfeet. We identified, among this transcriptome, a pool of messenger RNAs bound to ribosomes, the endfeetome, that primarily encodes for secreted and membrane proteins. We detected nascent protein synthesis in astrocyte endfeet. Finally, we determined the presence of smooth and rough endoplasmic reticulum and the Golgi apparatus in astrocyte perivascular processes and endfeet, suggesting for local maturation of membrane and secreted proteins. These results demonstrate for the first time that protein synthesis occurs in astrocyte perivascular distal processes that may sustain their structural and functional polarization at the vascular interface.

## Introduction

Astrocytes, which are the most numerous neuroglial cells in the central nervous system (CNS), are multipolar cells. Through specific physical coverage of neuron processes [1, 2], they control the formation, maturation and activity of synapses [3] by sensing neuronal inputs and in turn modulating neighboring synaptic elements through various mechanisms, such as uptake or release of neuroactive factors [4]. Astrocytes also send endfeet-terminated processes that contact blood vessels and fully sheath the brain vascular system [5]. There, they display a specific molecular repertoire that allows them to regulate perivascular homeostasis, blood–brain barrier (BBB) integrity, crosstalk with the peripheral immune system, endothelial transport and vessel contractility in response to neuronal activity [6–8]. Astrocyte polarity, which allows astrocytes to integrate and regulate neuronal and vascular signals, is crucial to the brain and its alteration is described in several neurological and psychiatric diseases [7, 9]. In particular, pathologies associated with vascular dysfunction, including epilepsy, ischemic brain damage and Alzheimer’s disease, have been coupled to the loss of astrocyte polarity at the vascular interface [10–13]. Thus, a question of prime importance is how such functional polarization is set in astrocytes. Interestingly, the surface of astroglial processes far exceeds that of the soma. Indeed, astrocytes (literally ‘star-like cells’) display a highly complex morphology, with numerous processes of ~50 μm in the gray matter (protoplasmic) or up to 300 μm in the white matter (fibrous). This morphology allows them to contact synapses and vessels and cover a surface area of ~60 000–80 000 μm^^2^^ [14]. This extreme level of ramification, the large volume occupied by astrocytes (~65 000 μm^3^ per protoplasmic astrocyte) and their capacity to dynamically regulate synaptic and vascular functions necessarily pose unique challenges for the optimization of protein availability in both space and time [14]. The compartmentalization of mRNAs to distal regions of the cytoplasm is one of the most prominent and evolutionarily conserved mechanisms for the spatial and temporal regulation of protein synthesis in polarized cells. It allows the establishment of specific molecular repertoires in subcellular domains in response to environmental cues, thereby contributing to cell polarity. In the brain, distal mRNA localization has been described in neurons, in which the cytoplasmic processes can reach more than 1000-fold the diameter of the cell body. It has been described in growing axons, growth cones and in mature dendrites, and has been suggested in mature axons (reviewed in Buxbaum *et al.* [15]). Moreover, a subpopulation of mRNAs has been shown to localize to distal neuronal processes [16].

Here we focused on the gliovascular interface and demonstrated for the first time that mRNAs distribution and protein synthesis occur distally in astrocyte perivascular endfeet.

## Results

### Astrocyte mRNAs are distributed in astrocyte perivascular processes and endfeet

**Figure 1:**
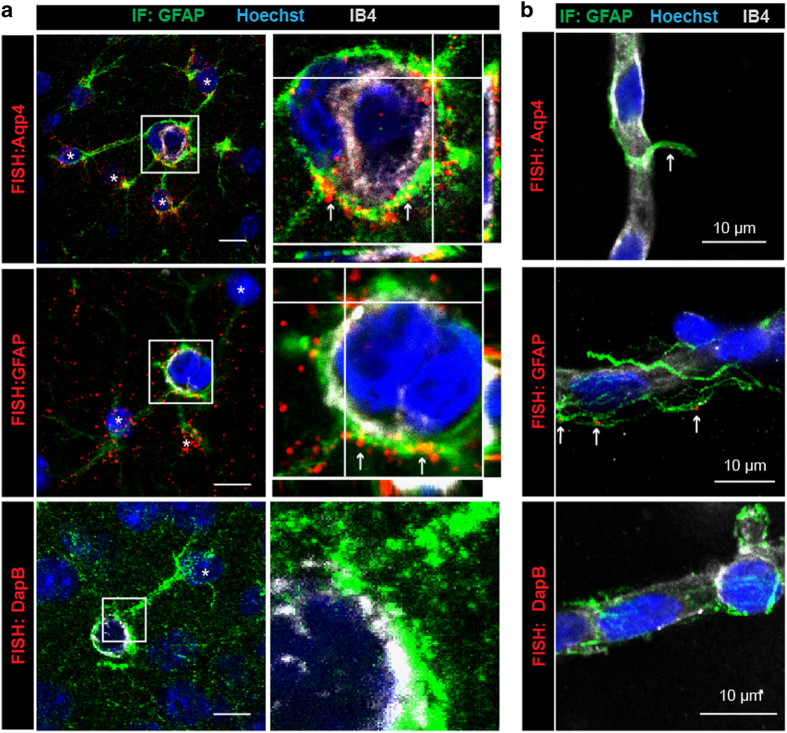
mRNAs are present in astrocyte perivascular processes and endfeet. (**a**) Representative confocal images of astrocyte-specific mRNAs encoding Aqp4, GFAP and dapB (negative probe) detected by fluorescent *in situ* hybridization (FISH by RNAscope) on brain sections of 2-month-old C57BL6 mice. Astrocytes (here in the hippocampus) are immunostained for GFAP (green). GFAP is a cytoskeleton protein specific to astrocytes whose immunolabeling indicates the presence of astrocyte processes and endfeet. GFAP fibers do not fill the endfeet, which explains why astrocyte FISH signals are not necessarily co-localized with GFAP. Aqp4 or GFAP mRNAs are mostly detected in PVAPs and endfeet (red) (white arrows). Few Aqp4 mRNAs are detected at the level of IB4. They might belong to vascular cells, although Aqp4 is mostly expressed by astrocytes [17, 18, 20]. The astrocyte somata are indicated with an asterisk. Enlarged views of boxed areas show details of the FISH signals in PVAPs and endfeet. (**b**) Representative confocal images of astrocyte-specific mRNAs encoding Aqp4 and GFAP on 2-month-old C57BL6 mice-purified brain vessels. Perivascular Aqp4 or GFAP mRNAs are detected at the level of GFAP immunolabeled fibers attached to the surface of purified brain vessels (red) (white arrows). Enlarged views of boxed areas show details of the perivascular FISH signals. In **a** and **b**, the vessel surface is stained with IB4 (gray) and nuclei with Hoechst (blue). Orthogonal analysis of areas indicated by white lines show the detail of FISH signals at the level of GFAP fibers at the vessel surface.

We explored the distribution in astrocytes of mRNAs encoding for astrocyte-specific proteins, Aquaporin 4 (Aqp4), a water channel enriched in the perivascular astroglial membrane [17, 18] and the cytoskeletal glial fibrillary acidic protein (GFAP), by performing high-resolution fluorescent *in situ* hybridization (FISH) on brain sections (Figure 1a). Co-immunostaining of GFAP, the main intermediate filament protein in astrocytes, allowed us to simultaneously visualize astrocytes and their processes, including perivascular astrocyte processes (PvAP) and endfeet. Vessel walls were labeled with Isolectin GS-B4 (IB4). Because the FISH procedure includes a mandatory protease digestion, we focused on the hippocampus, in which the GFAP immunolabeling was most preserved. Surprisingly, Aqp4 and GFAP mRNAs were not only present in the astrocyte somata but also scattered in GFAP-labeled processes. Moreover, most of the FISH signals were detected surrounding the IB4-stained vessel surface and at the level of GFAP-labeled fibers abutting vessels, regardless of their diameter. No FISH signal could be detected using the negative control probe against dapB (*Bacillus subtilis* dihydrodipicolinate reductase). These results suggest that astrocyte mRNAs are distributed in distal perivascular processes and endfeet.

### Characterization of the astrocyte endfeet transcriptome

**Figure 2:**
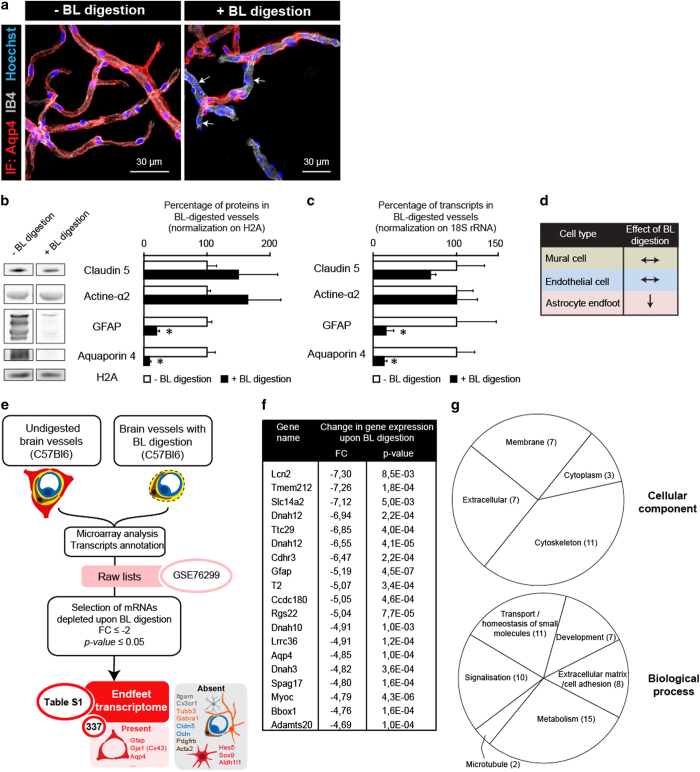
Characterization of the endfeet transcriptome. (**a**) Representative confocal images of purified brain vessels with or without partial basal lamina (BL) digestion. The vessel surface is stained with IB4 (gray). Astrocyte perivascular membrane are immunolabeled for Aqp4 (red). The nuclei are stained with Hoechst (blue). Digestion of the BL partially removes astroglial perivascular membranes (white arrows). (**b**) Comparative Western blot analysis of Claudin5 (Cldn5), Smooth muscle actin (Acta2), GFAP and Aquaporin 4 (Aqp4) protein levels in brain vessels purified from 2-month-old mice with or without partial BL digestion. Histone2A (H2A) was used as the loading control. The percentage of expression in digested vessels compared with that in undigested vessels (set as 100%; *n*=3): Cldn5 149±62%, *P*=0.2; Acta2 164±53%, *P*=0.2; GFAP 20±4%, *P*=0.05; Aqp4 8±2%, *P*=0.05. Mann–Whitney two-tailed test, *P=0.05. (**c**) Comparative quantitative PCR analysis of Claudin5 (Cldn5), Smooth muscle actin (Acta2), GFAP and Aquaporin 4 (Aqp4) transcripts in brain vessels purified from 2-month-old mice with or without partial BL digestion. The percentage of expression in digested vessels compared with that in undigested vessels (set as 100%; *n*=3): Cldn5 70±5% *P*=0.2; Acta2 100±2% *P*=0.2; GFAP 9±2%, *P*=0.05; Aqp4 6±1%, *P*=0.05. Mann–Whitney two-tailed test, *P=0.05. (**d**) Recapitulative table of partial BL digestion effects on purified brain vessels and associated astrocyte endfeet. (**e**) Analysis flowchart of the endfeet transcriptome. Transcripts depleted upon BL digestion with a fold-change (FC) ⩽−2 and a *P*-value ⩽0.05 were selected. The endfeet transcriptome displays 337 mRNAs including the astroglial-specific Gja1 (Cx43), Aqp4 or GFAP. Neuronal-, microglial-, endothelial- and mural cell-specific mRNAs are absent, as well as the astroglial-specific Hes5, Sox9 and Aldh1l1 indicating that only a subset of astrocyte mRNAs are distributed in endfeet. (**f**) List of the 20 most depleted mRNAs upon BL digestion compared with the undigested vessels. (**g**) Gene Ontology analysis of the ‘cellular component’ and ‘biological process’ pathways significantly depleted in the brain vessels upon BL digestion. The amount of pathways for each category is indicated.

Next, we aimed to characterize the pool of mRNAs present in astrocyte endfeet. In a previous study, we demonstrated that astrocyte endfeet perivascular membranes remain attached to mechanically isolated brain vessels of all sizes, whereas astrocyte cell bodies as well as oligodendrocytes, microglia and neurons (with the exception of few neuronal fibers) are lost during the purification [19]. Thus, we considered whether astrocyte mRNAs detected in endfeet (Figure 1a) would get trapped in the endfeet membranes that remain attached to the vessel walls during brain vessel purification. We tested this hypothesis by performing FISH on purified brain vessels (Figure 1b). Consistent with our hypothesis, discrete points of Aqp4 and GFAP FISH labeling were detected above the vessel surface labeled by IB4, at the level of GFAP-imunolabeled fibers. Thus, the presence of astrocyte mRNAs in purified brain vessels was likely due to the co-purification of perivascular astrocyte membranes left attached to the vessel surface. As these membranes are connected to the vessel surface by the basal lamina (BL), we next reasoned that a mild BL enzymatic digestion might detach them and possibly deplete brain vessels of astrocyte perivascular mRNAs, without altering their structure. Consistently, the uniform Aqp4 immunolabeling present at the surface of purified vessels, demonstrating the presence of perivascular astroglial endfeet membranes, became discontinuous after partial enzymatic BL digestion (Figure 2a). To confirm these results, we performed a western blot analysis of proteins extracted from purified brain vessels that were or were not treated for BL digestion (Figure 2b). It showed that Aqp4 or GFAP levels were strongly decreased upon digestion, in contrast to the level of mural (vascular smooth muscle cells (VSMCs) and pericytes) and endothelial cell proteins, smooth muscle aortic alpha-actin (Acta2) and Claudin 5 (Cldn5), respectively. We next compared by quantitative PCR the level of Aqp4, GFAP, Cldn5 and Acta2 mRNAs in purified undigested and digested brain vessels (Figure 2c). As observed for proteins, the level of astrocytic GFAP and Aqp4 mRNAs was strongly decreased upon BL digestion, while no effect could be detected for Cldn5 and Acta2. Collectively, these results strongly suggest that partial BL digestion removes astrocyte perivascular membranes and specifically depletes brain vessels of astroglial proteins and mRNAs but had no detectable effect on vascular cells (Figure 2d). Thus, the mRNAs most depleted from purified brain vessels upon BL digestion are those that are primarily present in astrocyte endfeet.

To further identify these transcripts, we compared mRNAs extracted from brain vessels purified from 2-month-old C57Bl6 mice, with or without BL enzymatic digestion. Four independent complementary DNA (cDNA) libraries were prepared for each condition (digested and undigested) and hybridized on an Affymetrix Mouse Gene 2.0 Array (Figure 2e). We selected transcripts depleted upon BL digestion with a fold-change (FC) ⩽−2 and a *P-*value ⩽0.05 (Figure 2e and f, Supplementary Table S1). A Gene Ontology (GO) analysis indicated that mRNAs depleted upon partial BL digestion encoded mostly membrane, cytoskeleton and secreted proteins mainly involved in metabolism, homeostasis, signalization, cell adhesion and development (Figure 2g, Supplementary Table S2). Consistent with our previous results, mRNAs encoding for GFAP and Aqp4 were among the markers most significantly depleted upon BL enzymatic digestion, along with other mRNAs encoding for known astroglial-specific proteins such as Connexin43 (Gja1) or Kir4.1 (Kncj10) (Figure 2e, Supplementary Table S1). In contrast, Hes5, Sox9 or Aldh1l1 mRNAs that are specific to astrocytes [20], were not depleted upon BL digestion (Figure 2e, Supplementary Table S1). The level of mRNAs encoding for known enriched markers of endothelial cells, mural cells or potentially expressed by macrophages present in perivascular spaces and neuronal fibers attached to the surface of the purified brain vessels [19], was not or weakly modified upon BL digestion (Figure 2e and f, Supplementary Table S1). Thus, the mRNAs depleted upon BL digestion were those co-purified with astrocyte perivascular membranes and present in astrocyte endfeet. To confirm these results, FISH detection on brain sections as well as purified brain vessels was performed for some of them (Supplementary Figure S1). Importantly, astrocyte endfeet transcripts represented a subset of astrocyte mRNAs. Due to the partial nature of BL digestion and the low sensitivity displayed by microarrays, the transcripts identified here were likely abundant in endfeet. They define the astrocyte endfeet transcriptome.

### Characterization of the astrocyte endfeet ribosomal-bound transcriptome

**Figure 3:**
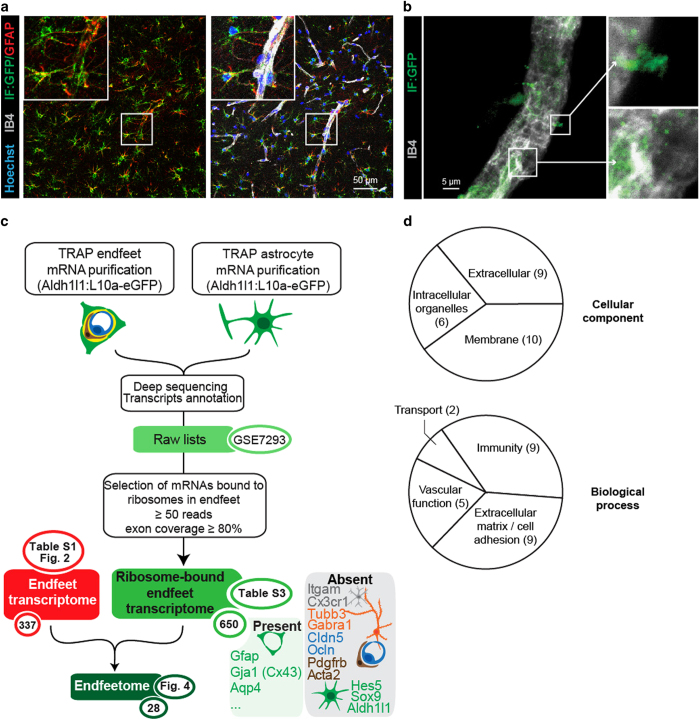
Analysis of the astrocyte endfeet ribosomal-bound transcriptome. (**a**, **b**) Representative confocal images of GFP-tagged ribosomes (green) in 2-month-old Aldh1l1:L10a-eGFP mice, (**a**) on brain section (here in the hippocampus) and (**b**) at the surface of purified brain vessels. Enlarged views of squared areas show details of perivascular astroglial ribosomes. The vessel surface is stained with IB4 (gray) and nuclei with Hoechst (blue). Astrocytes are immunostained for GFAP (red). (**c**) Flowchart for the combined analysis of the endfeet transcriptome and the endfeet and whole astrocyte TRAP ribosome-bound transcriptome. The selection criteria are detailed in the white box. Lists of transcripts and pathway analysis generated at each step are indicated in green circles, with the corresponding tables and the number of transcripts. The ribosome-bound endfeet transcriptome displays 650 mRNAs including the astroglial-specific Gja1 (Cx43), Aqp4 or GFAP. Neuronal-, microglial-, endothelial- and mural cell-specific mRNAs are absent, as well as the astroglial-specific Hes5, Sox9 and Aldh1l1 mRNAs indicating that only a subset of astrocyte mRNAs bound to ribosomes is present in endfeet. The combination of the endfeet transcriptome and the TRAP ribosome-bound transcriptomes defines the endfeetome, which displays 28 transcripts. (**d**) Gene Ontology analysis of the enriched ‘cellular component’ and ‘biological process’ pathways in the endfeet ribosome-bound transcriptome. Numbers indicate the amount of pathways for each category.

**Figure 4:**
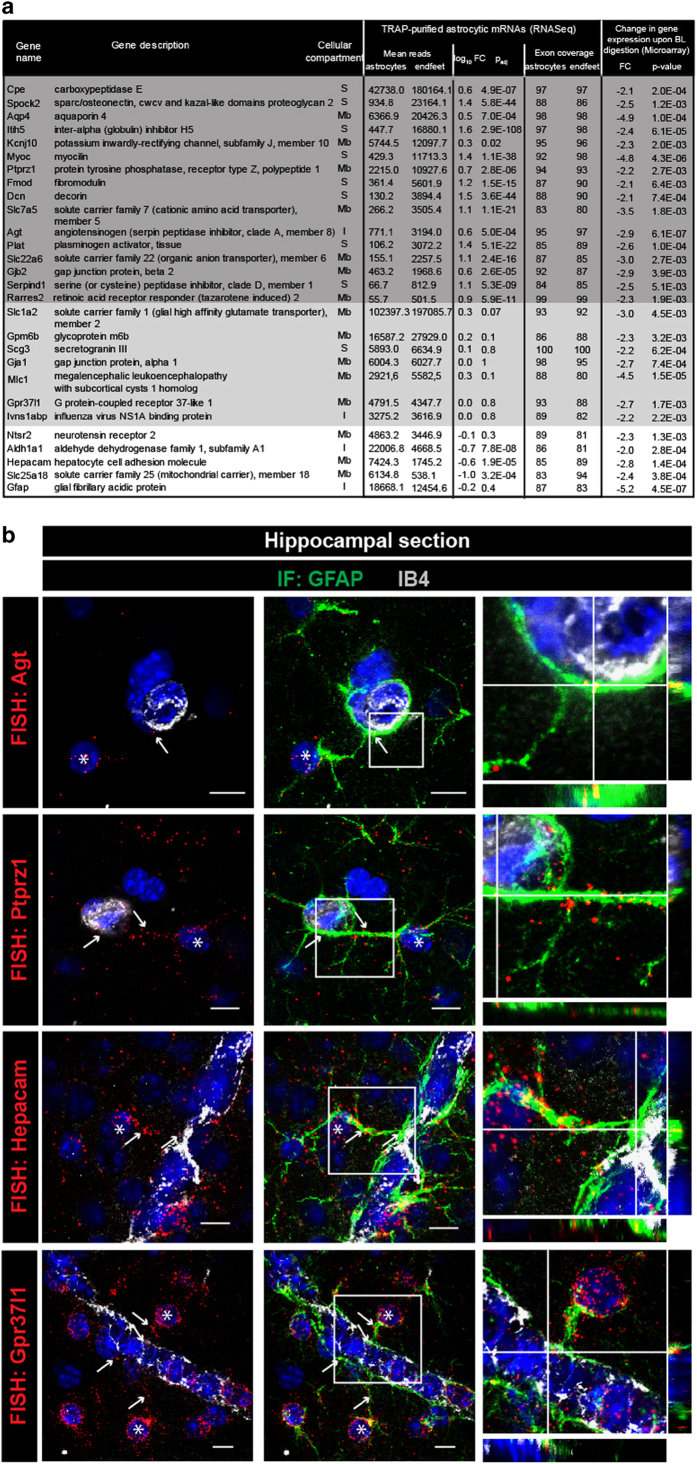
The endfeetome. (**a**) List of mRNAs. The endfeetome common to the endfeet transcriptome and the endfeet and whole astrocyte ribosome-bound (TRAP) transcriptomes. Endfeet mRNAs enriched in the whole astrocyte TRAP libraries compared to the endfeet TRAP libraries (RNAseq: log_10_ FC<0, *P*_adj_⩽0.05) are indicated in white. Endfeet mRNAs equally present in both types of TRAP libraries (RNAseq: *P*_adj_>0.05) are indicated in light gray. Endfeet mRNAs enriched in the endfeet TRAP libraries compared to whole astrocytes TRAP libraries (RNAseq: log_10_ FC>0, *P*_adj_⩽0.05) are indicated in dark gray. The cellular compartment of each corresponding protein is indicated (Mb, membrane; S, secreted; I, intracellular). (**b**) Representative confocal images of some endfeetome transcripts detected by FISH (red) in hippocampus slices. The vessel surface is stained with IB4 (gray) and nuclei with Hoechst (blue). The astrocytes are immunostained for GFAP. The astrocyte somata are indicated with an asterisk. The white arrows indicate extravascular FISH labeling on the vessels and at the level of GFAP positive filaments in PvAPs and endfeet. On the right, orthogonal analysis of areas indicated by white lines on the enlarged views shows the detail of FISH signals at the level of GFAP fibers at the vessel surface.

We then considered whether mRNAs of the astrocyte endfeet transcriptome were also bound to ribosomes in astrocyte endfeet and could potentially be involved in local translational events. We first explored the distribution of ribosomes in astrocytes on adult brain sections of the transgenic mouse strain Aldh1l1-eGFP/Rpl10a (Aldh1l1:L10a-eGFP), in which the ribosomal protein L10a is fused with eGFP and expressed under the control of the Aldh1l1 astrocyte-specific promoter [21] (Figure 3a). We observed that GFP-tagged ribosomes were present not only in astrocyte somata but also in processes labeled with GFAP, including PvAPs and endfeet (Figure 3a). Interestingly, astrocyte ribosomes were also observed at the surface of purified Aldh1l1:L10a-eGFP brain vessels of all types (Figure 3b). Together with our precedent results, these observations suggested the possibility that some endfeet mRNAs were bound to ribosomes. We combined our previously described brain vessel isolation with an astrocyte-specific translating ribosome affinity purification (TRAP) [21, 22] (Figure 3c). Brain vessels were purified from 2-month-old Aldh1l1:L10a-eGFP mice, GFP-tagged ribosomes retained within astrocyte endfeet perivascular membranes were immunoprecipitated, and ribosome-bound mRNAs were extracted. The same TRAP procedure was performed on whole brains to extract whole astrocyte ribosome-bound mRNAs. After reverse-transcription, the cDNA libraries were sequenced (3 ‘whole astrocyte TRAP’ libraries and 6 ‘endfeet TRAP’ libraries), and raw data were analyzed following the flowchart detailed in Figure 3c. The reads were first aligned against the *Mus musculus* genome to identify the corresponding genes. The raw lists were then refined to keep mRNAs with more than 50 reads and exon coverage ⩾80% in order to only select complete mRNAs representing potential translational events. Since all endfeet transcripts should be sequenced in whole astrocytes, only transcripts present in both lists were further considered (Supplementary Table S3). Importantly, most mRNAs encoding for known enriched markers of endothelial cells, mural cells, oligodendrocytes, neurons or microglia/macrophages were absent from this refined list, in contrast to mRNAs encoding for known astroglial-specific proteins such as Connexin43 (Gja1), GFAP or Aqp4 [20] (Figure 3c, Supplementary Table S3). As previously found in the endfeet transcriptome, mRNAs encoding for Hes5, Sox9 or Aldh1l1 were not present in endfeet (Figure 3c, Supplementary Table S3). Similarly to the endfeet transcriptome, GO analysis revealed that the extracellular and membrane compartments were the most highly enriched ‘cellular component’ pathways in the endfeet-TRAP list and immune processes and cell adhesion were the most enriched ‘biological process’ pathways, suggesting for segregation of specific ribosome-bound mRNAs in endfeet (Figure 3d, Supplementary Table S4). We then considered the ribosomal status of the endfeet transcriptome combining it with the whole astrocyte and endfeet TRAP lists (Figure 3c). Among the 310 mRNAs of the endfeet transcriptome, 28 that mainly encoded for secreted and membranous proteins were present in the endfeet-TRAP list, thus possibly bound to ribosomes. We defined this list as the endfeetome (Figure 4a). Among these transcripts, 7 were equally detected in whole astrocytes and in endfeet TRAP lists (*P*_adj_>0.05); 5 were enriched in the whole astrocyte compared to the endfeet TRAP lists (log_10_ FC<0, *P*_adj_⩽0.05) thus potentially more abundant in the astrocyte soma; 16 were enriched in the endfeet TRAP lists compared to whole astrocytes (log_10_ FC⩾0, *P*_adj_⩽0.05) and were thus likely more abundant in the endfeet. This last category might represent the most abundant and most translated endfeet mRNAs. The presence of some of these mRNAs in the endfeet was further confirmed by performing high-resolution FISH on brain sections (Figure 4b). As already shown for Aqp4 and GFAP mRNAs (Figure 1a), Agt, Ptprz1, Hepacam or Gpr37l1 mRNAs were detected in most hippocampal astrocytes as discrete fluorescent points in the soma and along GFAP-immunolabeled PvAPs and endfeet surrounding IB4-stained vessels.

Here we describe a pool of ribosome-bound mRNAs abundant in astrocyte perivascular endfeet that primarily encode for membrane and secreted proteins.

### Protein translation occurs in astrocyte endfeet

**Figure 5:**
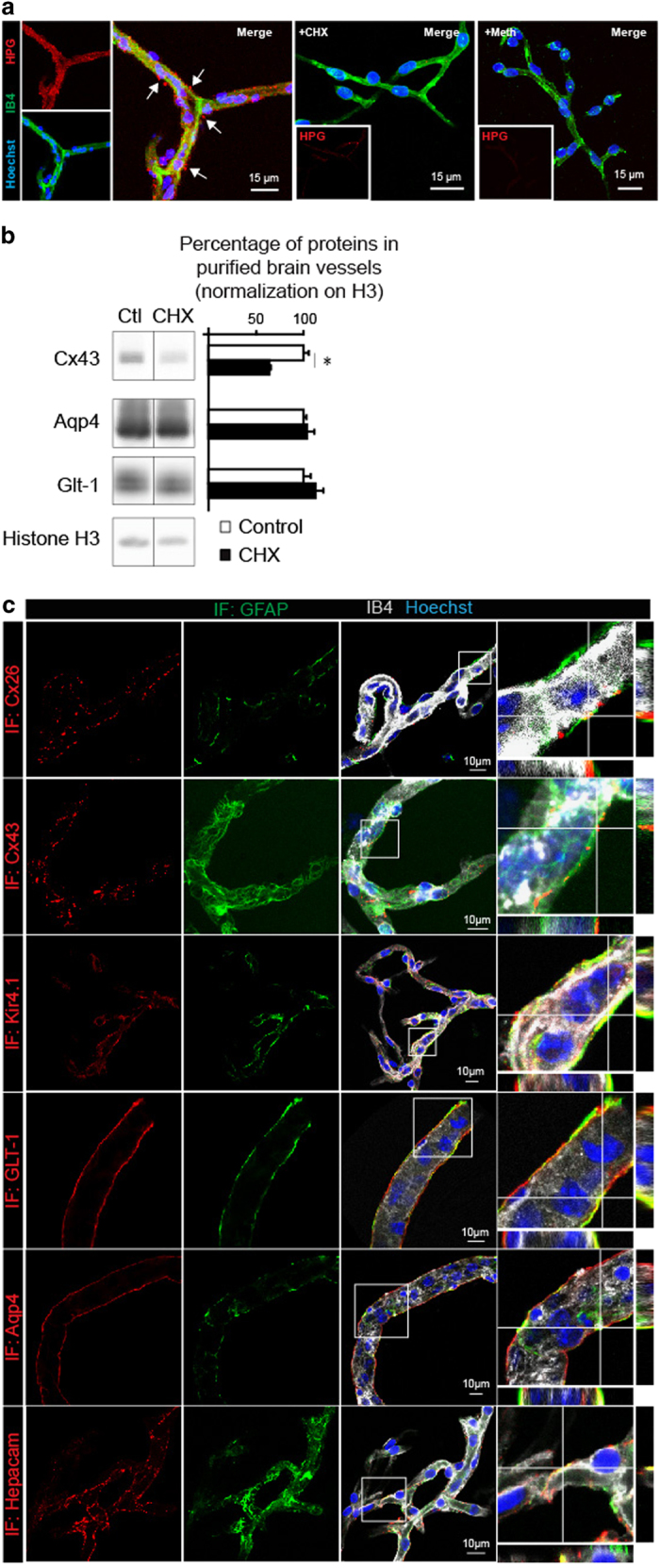
Protein translation occurs in astrocyte perivascular endfeet. (**a**) Representative confocal images of Click-iT HPG-labeled brains vessels purified from 2-month-old C57BL6 mice (red). The vessel surface is stained with IB4 (green) and nuclei by Hoechst (blue). Negative control experiments were performed using Cycloheximide (+CHX) or replacing HPG by methionine (+Meth). The white arrows indicate extravascular HPG labeling. (**b**) Cx43 turnover in endfeet is sustained by local protein translation. Western blot detection of Cx43, Aqp4 and Glt1 in protein extracts from brain vessel-associated astrocyte endfeet purified from 2-month-old C57BL6 mice submitted or not to Cycloheximide (+CHX). Protein level in untreated samples is set as 100% (*n*=3): Cx43 64±1%, *P=0.05; Aqp4 103±8%; Glt1 113±8%. Mann–Whitney two-tailed test. (**c**) Representative confocal images of immunofluorescent detection of endfeetome proteins in astrocyte endfeet (red) on 2-month-old C57BL6-purified brain vessels. The vessel surface is stained with IB4 (gray) and nuclei with Hoechst (blue). Astrocyte endfeet are immunolabeled for GFAP (gray). Enlarged views of boxed areas show details of immunofluorescence. Orthogonal analysis of areas indicated by white lines to show the detail of FISH signals at the level of GFAP fibers at the vessel surface.

The identification of ribosome-bound mRNAs in astrocyte endfeet strongly suggests that protein translation occurs on site. To detect protein translation in astrocyte endfeet, we combined brain vessel co-purification of astrocyte perivascular endfeet with the Click-iT HPG (Homoproparglyglycine) protein synthesis assay (Figure 5a). HPG is an amino-acid analog of methionine containing an alkyne moiety detectable by ligation with an Alexa Fluor azide when incorporated in nascent proteins [23]. Incubation of purified brain vessels during 1 h with HPG resulted in an intense signal in these vessels. Strikingly, perivascular areas at the level of astrocytic endfeet were also labeled. No signal was observed in vessels pretreated with cycloheximide (CHX), an inhibitor of protein synthesis, or when HPG was replaced by methionine. Thus, we here detected nascent protein translation in astrocyte endfeet.

Next, we reasoned that inhibiting protein synthesis in endfeet separated from the astrocyte somata might result in the rapid decrease of astrocyte-specific markers of the endfeetome exhibiting a short half-life. Cx43 (Gja1) life cycle is known as a dynamic process. It has been invariably measured around 1.5–5 h regardless of the cell type, leading to the rapid turnover of gap junction plaques [24–26]. In contrast, the half-life of Aqp4 would be >8 h [27] and >24 h for Glt1 (Slc1a2) [28]. We compared the level of these proteins on western blot of proteins extracted from brain vessel-associated endfeet treated or untreated with CHX for 6 h (Figure 5b). Histone 3 that has a half-life of several days [29] served as the loading control. Interestingly, although Aqp4 and Glt1 showed the same level in both conditions, Cx43 decreased by half upon CHX treatment, suggesting that translation in astrocyte endfeet sustains the rapid turnover of Cx43.

Finally, we expected proteins of the endfeetome to locate in the astrocyte endfeet. We tested this hypothesis performing immunodetection on purified brain vessels focusing on membrane endfeetome components (Figure 5c). Astrocyte endfeet were immunolabeled for GFAP in parallel, and the vessel surface was stained with IB4. Aqp4, Glt1 (Slc1a2) and Kir4.1 (Kcnj10) form a uniform labeling corresponding to the perivascular endfeet membranes. A classical punctiform gap junction perivascular signal was observed for Cx26 (Gjb2) and Cx43 (Gja1). Hepacam labeling was also linear around vessels in areas that might correspond to endfeet membrane contacts [30]. Thus, these results demonstrate the occurrence of translation as well as the presence of proteins from the endfeetome in astrocyte endfeet.

### Subcellular organization of astrocyte PvAPs and endfeet suggests for local maturation and sorting

**Figure 6:**
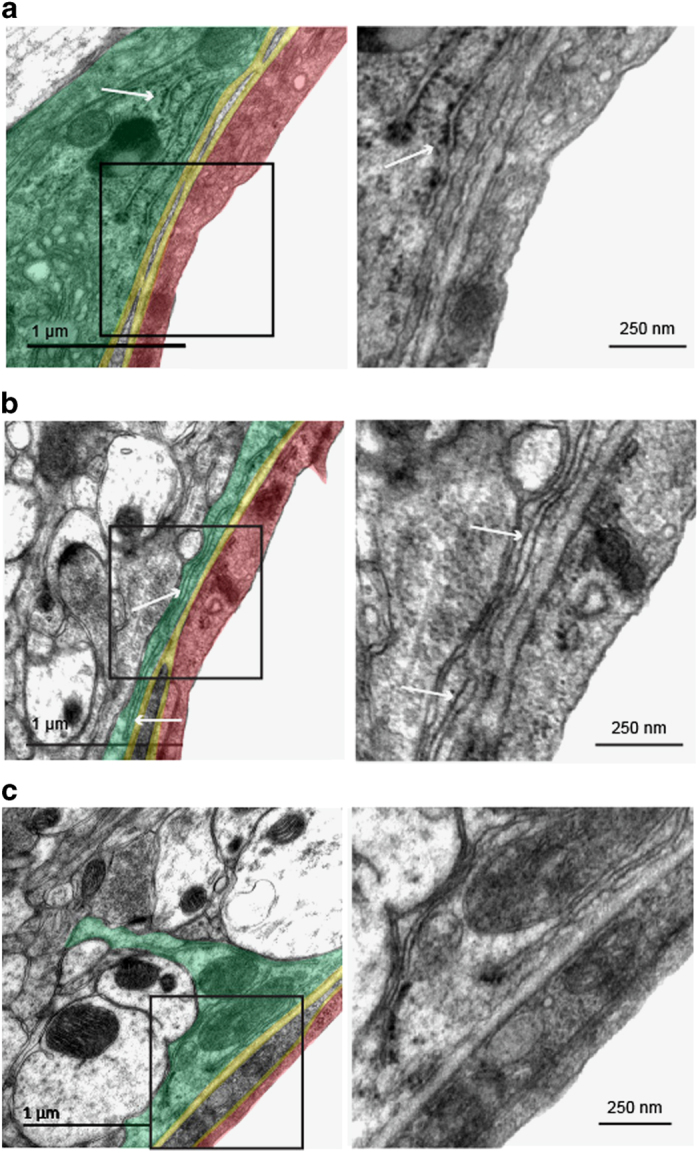
Organization of endoplasmic reticulum in astrocyte endfeet. Representative transmission electron microscopy images showing endoplasmic reticulum (ER) in cortical astrocyte endfeet surrounding 5–20 μm diameter vessels of 2-month-old C57BL6 mice. On the left images, the different structures of the gliovascular unit are colored: an astrocyte endfoot (green), the basal lamina (yellow), an endothelial cell (red). Enlarged views of squared areas show details of the ribosomes and ER structures. (**a**) An astrocyte endfoot with rough ER (RER) (white arrow). (**b**) An astrocyte endfoot with smooth ER (SER) (white arrow). (**c**) An astrocyte endfoot with SER with areas containing few bound ribosomes (white arrow).

**Figure 7:**
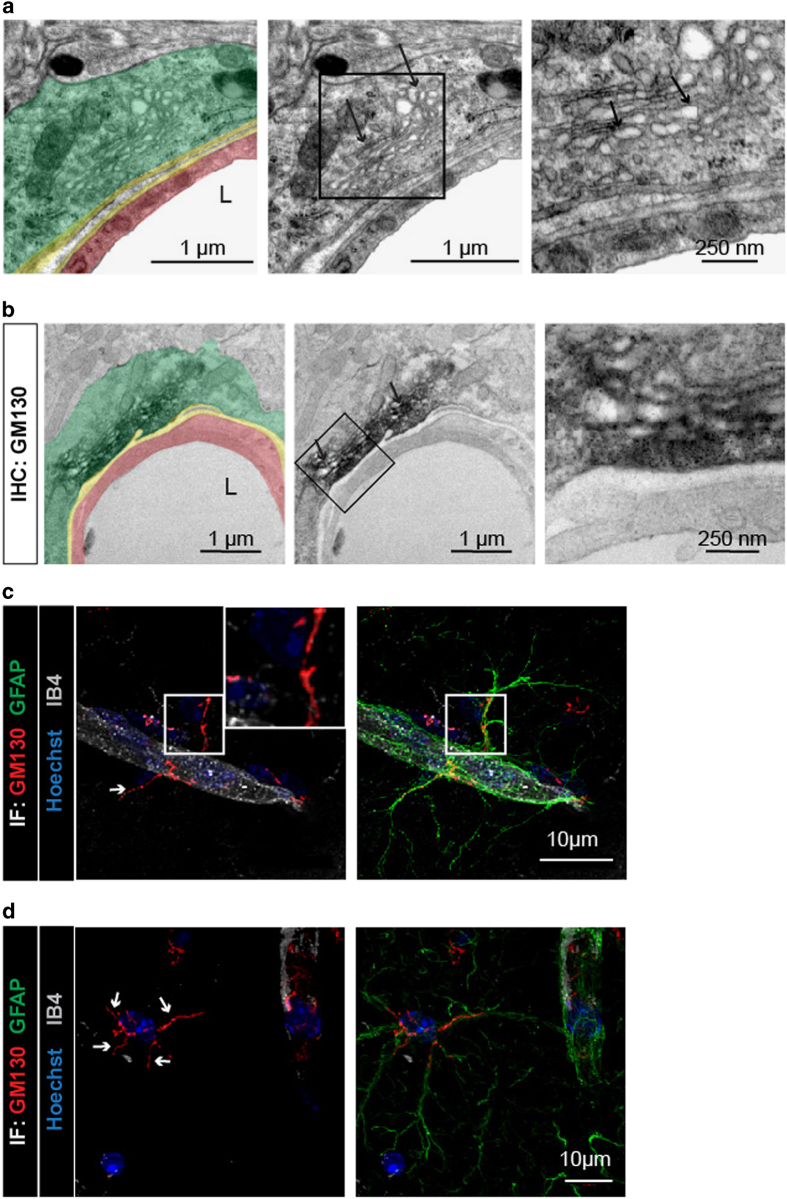
Organization of the Golgi apparatus in astrocyte perivascular processes and endfeet. (**a**, **b**) Representative transmission electron microscopy images of the Golgi apparatus (GA) in cortical astrocyte endfeet surrounding 5–20 μm diameter vessels of 2-month-old C57BL6 mice. Enlarged views of the squared areas show details of GA in the astrocyte endfeet. Black arrows indicate typical GA stacks with surrounding vesicles. On the left images, the different structures of the gliovascular unit are colored: an astrocyte endfoot (green), the basal lamina (yellow), an endothelial cell (red). (**b**) Immunolabeling for the cis-GA protein GM130 in an astrocyte endfoot. (**c**, **d**) Representative confocal images of cortical astrocytes immunolabeled for GFAP (green) and GM130 (red) on brain sections of 2-month-old C57BL6 mice. The vessels surface is stained with IB4 (gray) and nuclei with Hoechst (blue). The white arrows show the GA ramifications in the PvAPs and endfeet.

Most secreted or integral plasma membrane proteins undergo folding and maturation processes in the endoplasmic reticulum (ER) and the Golgi apparatus (GA) before reaching their final destination. The presence of ribosome-bound mRNAs encoding for such proteins in the astrocyte endfeet suggested that alternative maturation and secretory routes distal to the soma might be present in the endfeet. To address this question, we performed a transmission electron microscopy (TEM) study that focused on the astrocyte endfeet surrounding 5- to 20-μm-diameter vessels in the adult mouse cortices (*n*=78 in three mice). Remarkably, large networks of flattened *cisternae* with wavy membranes and clear lumen typical of the ER were systematically observed in the endfeet (89±12%) (Figure 6). These membranes often ran in parallel and close to the endfeet perivascular plasma membrane. ER membranes fully covered with ribosomes (thus, rough endoplasmic reticulum (RER)) were observed in 20±14% of the endfeet (Figure 6a). In 52±22% of the endfeet, SER devoid of ribosomes was present (Figure 6b). Finally, tubules of SER with few associated ribosomes were observed in 57±5% of the endfeet (Figure 6c). Compared to the abundance of ER, stacks of GA *cisternae* immunolabeled by the *cis*-GA marker GM130 and surrounded by numerous vesicles were present in few endfeet (6±4%) (Figure 7a and b). To characterize the global morphology of GA in the astrocyte PvAPs and endfeet, we developed an immunohistological approach combined with confocal microscopy (Figure 7c and d). GA was labeled for GM130 and astrocyte processes and endfeet were labeled for GFAP. Vessels were counterstained with IB4. We restricted our analysis to PvAPs surrounding vessels measuring 5–20 μm in diameter in the cortices of adult mouse brains (*n*=44 in four mice). A three-dimensional reconstruction and calculation of the PvAP length were performed [31]. For each analyzed astrocyte, the center of the nucleus was used as the starting point to calculate the lengths of PvAPs and GA processes. Only continuously GFAP-labeled PvAPs were considered. Surprisingly, in contrast to its typical central or perinuclear distribution, the GAs in these astrocytes were ramified in PvAPs in 72% of the cells; they were either continuous, or they formed detached GM130-labeled GA outposts (GOPs) (Figure 7c). In some astrocytes, the GA was ramified in several GFAP-labeled processes (Figure 7d). When the GA formed GOPs in PvAPs, the last one was considered to be the end of the GA branch. The length of the PvAPs containing the GA (21.9±5.7 μm) did not differ from that of the PvAPs without the GA (26.1±6.2 μm). In addition, when it was present in the PvAPs, the GA extended from the nucleus up to 12.3±3.7 μm, thus covering 58±20% of the PvAP length and reaching endfeet in 7% of the cells, as previously observed by TEM. Altogether, these data demonstrate that protein maturation and secretory organelles are present in PvAPs and astrocyte endfeet, which strongly suggests the existence of soma-independent routes for protein trafficking in PvAPs.

## Discussion

**Figure 8:**
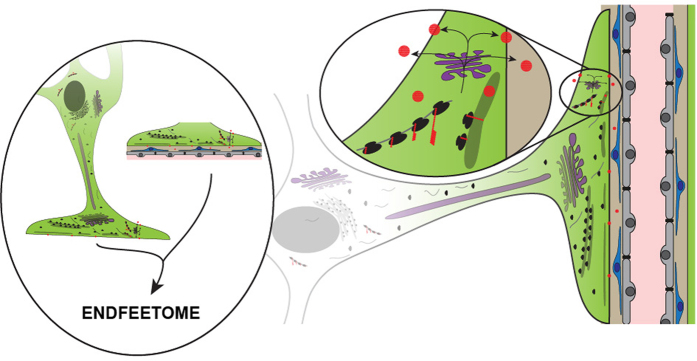
Astrocytes set protein synthesis in distal perivascular processes: graphical abstract. Astrocytes, the most numerous neuroglial cells in the central nervous system, are multipolar cells. They extend long processes terminated by endfeet (in green) at the surface of brain vessels (composed by mural cells in blue and endothelial cells in gray) and regulate vascular functions. In the present study, we demonstrate that some mRNAs (gray lines) are transported in astrocyte perivascular endfeet and bound to ribosomes (black dots) suggesting that they are translated on site. We also show that protein synthesis (red dots) occurs in endfeet. Finally, we show that endfeet are equipped with smooth and rough endoplasmic reticulum (gray) and the Golgi apparatus (purple), suggesting that the maturation of membrane and secreted proteins may occur locally. These results suggest that alternative routes for the translation, maturation and secretion of a specific pool of proteins are organized in the astrocyte perivascular endfeet. Proteins synthesized there might be either translated in the cytosol or in the RER. They can be further maturated in the local Golgi apparatus, inserted in the membrane or secreted in the perivascular space.

Until now, the proteins required for astrocyte functions encoded by nuclear genes were thought to be produced exclusively in the soma. Our observation of ribosome-bound mRNAs in perivascular endfeet together with the detection of nascent proteins and the presence of organelles for protein maturation and sorting demonstrated for the first time that astrocytes organize distal translation for a subset of mRNAs at the vascular interface and strongly suggested for local protein maturation (Figure 8).

Our approach to identify mRNAs present in astrocyte endfeet was based on the co-purification of astrocyte endfeet membranes attached to the surface of brain vessels [19]. These membranes retained mRNAs present in the endfeet. We set a BL-digestion procedure to allow the removal of these membranes. Importantly, a total BL digestion would have resulted not only in the loss of astrocyte endfeet but also of mural cells embedded in the BL. Hence, we only performed a partial BL digestion. Under these conditions, the level of known mural- and endothelial-specific or enriched mRNAs and proteins was not affected indicating that digestion had no noticeable effect on these cells. In contrast, some known astrocyte-specific or -enriched mRNAs and proteins were strongly depleted indicating the diminution of astroglial material. This result allowed us to develop a subtractive strategy comparing purified brain vessels submitted or not to BL digestion to identify mRNAs present in astrocyte endfeet. Importantly, the use of partial BL digestion combined with the low sensitivity of the microarray likely resulted in the exclusion of transcripts present only in low abundance in endfeet compared to the vascular cells. Finally, our study indicated that some of the known astrocyte-specific mRNAs, such as Hes5, Sox9 or Aldh1l1, were not present in endfeet, suggesting that the distribution of mRNAs in endfeet is selective and defines another level of astrocyte polarization at the vascular interface.

Seeking for the translational status of endfeet mRNAs ribosomal-bound profile of endfeet mRNAs, we noticed that some mRNAs identified in the endfeet transcriptome were absent in the TRAP libraries, suggesting that they might be abundant in endfeet but not or weakly translated. This was the case for Lcn2, encoding the Neutrophil Gelatinase-Associated Lipocalin (NGAL or Lipocalin 2) [32], one of the most depleted mRNAs upon BL digestion, which was not extracted by TRAP. This result suggested that Lcn2 mRNAs in endfeet may stay in a likely dormant and translationally repressed state in astrocyte endfeet. Considering that mRNA transport to distal parts requires high levels of energy, this pool of mRNAs in astrocytes may play a specific role and serve as a local supply that is easily reactivated upon specific conditions, such as neuroinflammation in the case of Lcn2 [33].

Interestingly, the endfeet transcriptome and endfeetome were mostly composed of secreted or plasma membrane proteins, highlighting the fact that mRNAs distribution and translation are compartmentalized in astrocytes. Among the endfeetome, we found molecules already known to control cerebrovascular functions such as Cx43 (Gja1), which is implicated in BBB immune quiescence [8, 34], Agt, which plays a role in BBB integrity [35], or Aqp4, Kir4.1 (Kncj10), Hepacam and Mlc1, which are all involved in perivascular homeostasis [13, 30, 36]. Their presence strongly suggested that the endfeetome represents important astroglial functions for the regulation of the brain vascular physiology. Overall, a quite novel molecular landscape emerged from this study at the astrocyte–gliovascular interface, which might reveal new fundamental aspects of the gliovascular physiology.

Another crucial point raised by our study is the observation of the ER and GA in the PvAPs and endfeet. Structures known as ribosome-associated contacts with the plasma membrane have been described in rodent and human astrocyte processes [37]. However, to our knowledge, our results are the first demonstration that these organelles ramify in astrocyte perivascular processes *in vivo*. We systematically observed ER in astrocyte endfeet, suggesting that it may form a continuous endomembrane extending from the soma to the endfeet. The ER was complex, with areas containing either SER or RER or both. SER could also be associated with only few ribosomes. The functional relevance of this complexity is an open question. ER not only ensures the proper conformational maturation of nascent proteins but also organizes lipid synthesis. Notably, we observed contact sites between the ER and the astrocyte endfeet plasma membranes (Supplementary Figure S2), suggesting that direct non-vesicular lipid transfer through the ER may sustain endfeet membrane dynamics, as recently found in neurons [38]. The ER is also a dynamic Ca^2+^ reservoir and an indispensable Ca^2+^ source for fast physiological signaling [39]. Thus, the ER in astrocyte endfeet may sustain Ca^2+^-evoked responses [40, 41] and control neurovascular coupling [42] or Ca^2+^-dependent gliotransmission to nearby synapses, as discussed below [43]. Finally, the ER interacts physically with mitochondria, which form continuous networks in endfeet [5] and might thus regulate mitochondrial Ca^2+^ homeostasis [44]. In contrast to the ER, the GA rarely reached the endfeet and was either continuous or formed detached outposts (GOPs), which suggested for local protein secretion. The GA was labeled by GM130, a protein that connects the *cis* medial and *trans* GA compartments together [45]. Consistently, the GA observed by TEM displayed a classical multi stack structure, while not ruling out the existence of single GA compartments. The presence of GA in PvAPs was not systematic, likely illustrating specificities among astrocytes. Altogether, our observation of ER and GA structures in PvAPs and astrocyte endfeet and the identification of local translation in endfeet strongly suggest that local routes for protein transport using local ER and GA may coexist with the canonical soma to endfoot route.

**Figure 9:**
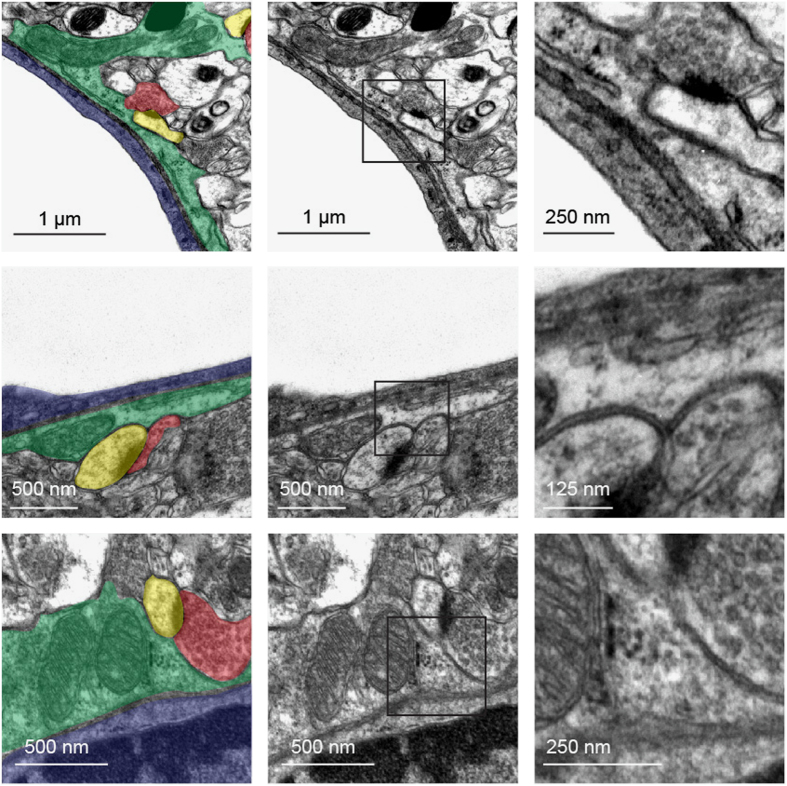
Presence of synapses abutting astrocyte endfeet. Representative transmission electron microscopy images of cortical astrocyte endfeet surrounding capillaries of a 2-month-old C57BL6 mouse. Enlarged views of the squared areas show details of the synapses abutting astrocyte endfeet. On the left image, astrocyte endfeet are colored in green, endothelial cells in blue, pre-synapses in red and post-synapses in yellow.

Our TEM study eventually led us to observe numerous synaptic elements (pre- and postsynaptic) in close vicinity to the perivascular endfeet (Figure 9). The tight coupling between astrocyte, vascular and neuronal activities has already been well described. Neuronal activity and energy supply by astrocytes are tuned together [46]. Control of water fluxes and extracellular space volume by astrocytes modifies synaptic activity; [47] astrocytes regulate neurovascular coupling [48], likely at the process level [42], and they secrete gliotransmitter modulating synaptic activity [49]. The close proximity of some synapses to astrocyte endfeet strongly suggests that astrocyte, neuronal and vascular activities can be integrated in both space and time. Consequently, local protein synthesis in endfeet might be dedicated to both gliovascular and neuroglial interactions. For instance, within the endfeetome, Myocilin [50, 51], Ptprz1 [52], the tissue plasminogen activator (tPA) [53] and the proteoglycan testican-2 (Spock2) [54] have all been shown to regulate neuronal architecture. The solute carriers Slc1a2 and Slc25a18 both promote glutamate transport at the plasma membrane and in the inner mitochondrial membrane respectively and might work in concert to regulate extra-synaptic level of glutamate and astrocyte energetic [55].

Here we demonstrate that astrocytes organize protein synthesis at the vascular interface. We characterize abundant ribosome-bound transcripts in endfeet that might constitute novel astroglial pathways regulating gliovascular interactions. Finally, we describe protein maturation and secretion organelles in astrocyte perivascular endfeet. Which mechanism(s) are involved in the transport of mRNAs to PvAPs and endfeet for translation, how this transport is regulated and whether the use of routes for protein transport is plastic and adapts to specific signals are the next outstanding questions raised by our results. Spatiotemporal regulation of protein synthesis is a general mechanism to regulate homeostasis and plasticity. Astrocyte endfeet are interfacing vessels and neurons, and control mechanisms such as BBB integrity, basal lamina composition, immune quiescence and vessel contractility, as well as neuronal activity by funneling among others extracellular potassium and glutamate [4, 7]. These functions might well be controlled by local RNA synthesis at the endfeet, as suggested by the local translation of Aqp4, Kir4.1 and Glt-1, which are key regulators of the perivascular and neuronal homeostasis [56, 57]. Local translation in astrocyte endfeet might also be involved in neurological disorders as demonstrated in neurons in the case of X-fragile syndrome [58], amyotrophic lateral sclerosis [59, 60] or spinal muscular atrophy [61, 62].

Based on the present study, we propose that local protein synthesis may organize a spatially controlled protein delivery in astroglial perivascular endfeet thereby maintaining the polarized functions of astrocytes at the vascular interface. Importantly, the existence of local protein translation and maturation in astroglial perivascular processes conceptually changes the way we think about local astrocyte-vascular signalling. As biochemical compartmentalization supports structural cellular polarization, we propose that the synthesis of a specific molecular repertoire in astrocyte perivascular processes might contribute to the astrocyte polarized functions at the vascular interface.

## Materials and Methods

### Mice

Tg(Aldh1l1-eGFP/Rpl10a) JD130Htz (MGI: 5496674) (Aldhl1:L10a-eGFP) were obtained from the laboratory of Nathaniel Heintz (Rockefeller University, NY, USA) and kept in pathogen-free conditions. This mouse strain has been generated by bacterial artificial chromosome (BAC) transgenesis [21, 63]. Genotyping protocol is described in the bacTRAP project web site: www.bactrap.org*. C57BL6* mice were purchased from Janvier Labs (France) and kept in pathogen-free conditions.

### Antibodies

See Supplementary Table S5 for antibodies references and applications.

### Study approval

Experiments and techniques reported here complied with the ethical rules of the French agency for animal experimentation.

### Isolation of brain vessels

Mechanical purification of brain vessels from 2-month-old mice was performed as previously described [19]. A volume of 100 μl ml^−1^ cycloheximide was added to all buffers when using Aldhl1:L10a-eGFP mice (see below). Brain vessels submitted to basal lamina digestion were obtained replacing brain mechanical homogenization by a 45 min digestion with DNAseI (Sigma, St Louis, MO, USA) (20 U ml^−1^) and Liberase DL (Dispase Low, Sigma; 37.5 μg ml^−1^) in HBSS (Life Technology) at 37 °C.

### High-resolution fluorescent *in situ* hybridization by RNAscope (FISH by RNAscope)

FISH was performed on frozen brain sections or purified brain vessels immobilized on a glass slide coated with Cell Tak (Corning, Corning, NY, USA), following the RNAscope procedures (Advanced Cell Diagnostics, Inc., Newark, CA, USA). Hybridization of a probe against the *Bacillus subtilis* dihydrodipicolinate reductase (dapB) gene was used as negative control. At least three independent experiments have been performed and imaged. The probe list is given in the Supplementary Informations.

### Click-iT HPG Alexa Fluor Protein synthesis assay

Mechanically purified brain vessels from four 2-month-old C57BL6 [19] were resuspended in methionine-free DMEM (Thermo Fisher, Waltham, MA, USA) with Homoproparglyglycine (HPG), RNAse inhibitors (Thermo Fisher) and EDTA-free protease inhibitors (Roche, Boulogne-Billancourt, France) according to the manufacturer’s instructions and incubated 1 h at 37 °C under gentle rotation. Vessels were then washed in PBS, fixed in PBS/PFA 4% 15 min and permeabilized in PBS/Triton X-100 0.5% 20 min at room temperature. HPG incorporation was revealed using the Click-iT reaction cocktail according to the manufacturer’s instructions. Nuclei were stained with Hoechst (1:2000) and the vessel surface with Isolectine GS-B4 (1:100). The specificity of HPG labeling was assessed incubating the brain vessels with normal DMEM containing methionine or with 500 μg ml^−1^ HPG and 500 μg ml^−1^ Cycloheximide (Sigma) for 30 min at 4 °C prior to and during the incubation. Three independent experiments have been performed and imaged.

### Microarray study

Total RNAs were extracted from 2-month-old C57BL6 brain vessels submitted or not to basal lamina digestion using the Rneasy Lipid tissue kit (Qiagen, Germany, Hilden). Four independent libraries were prepared for each condition on purified vessels pooled from four mice. Affymetrix Mouse Gene 2.0 Array data sets were controlled using Expression console (Affymetrix, Santa Clara, CA, USA) and further analyses and visualization were made using EASANA (GenoSplice Technology, www.genosplice.com), which is based on the GenoSplice’s FAST DB annotations [64]. Gene Array data were normalized using quantile normalization. Background corrections were made with antigenomic probes selected as described previously [64]. Only probes targeting exons annotated from FAST DB transcripts (release 2015_1) were selected to focus on well-annotated genes whose mRNA sequences are in public databases [64]. Bad-quality selected probes (for example, probes labeled by Affymetrix as ‘cross-hybridizing’) and probes for which intensity signal was too low compared to antigenomic background probes with the same GC content were removed from the analysis. Only probes with a DABG *P*-value ⩽0.05 in at least half of the arrays were considered for statistical analysis [64]. We performed an unpaired Student’s *t*-test to compare gene intensities in the different biological replicates. Genes were considered significantly regulated when fold-change was ⩾2 and uncorrected *P*-value ⩽0.05. For hierarchical clustering, the distance from the gene signal in a given sample to the corresponding average in all the samples was calculated for each regulated gene. Corresponding values were displayed and clustered with MeV4.9.0 from the Institute of Genome Research using Euclidean distance and average linkage clustering. Raw data files are available on the GEO repository (www.ncbi.nlm.nih.gov/geo/) under accession number GSE76299.

#### GO analyses

We used the text-mining Pathway Studio ResNet database (Ariadne Genomics, Rockville, MD, USA) and the GSEA tool [65] in Pathway Studio 11.0.5 [66] to identify overrepresented signaling pathways and biological processes within our differentially expressed data set. As parameters for the GSEA method, we selected the Mann–Whitney *U*-test, a *P*-value threshold of 0.05.

### Aldhl1:l10a-eGFP TRAP RNA sequencing and analysis

One whole brain was used for each whole astrocyte polysome extraction (*n*=3 libraries). Choroid plexus were removed prior to extraction. For endfeet polysome extraction, brain vessels purified from eight mice were pooled (*n*=6 libraries). Polysome extraction was performed following the protocol described in the bacTRAP project web site: *www.bactrap.org* and by Heiman *et al.* [22], except that vessels were homogenized in a 2 ml Teflon-glass homogenizer with 25 strokes instead of 12 for the total brain.

#### Library preparation and Illumina sequencing

An amount of 10 ng of total polysomal RNAs were amplified and converted to cDNA using NuGEN’s Ovation RNA-Seq kit. Following amplification, 1 μg of cDNA was fragmented to ∼300 bps using Covaris S200. The remainder of the library preparation was done using 200 ng of cDNA following TruSeq RNA Sample Prep v2 kit from the End Repair step. Libraries were multiplexed by 3 on 3 flow cell lanes. A 50 bp read sequencing was performed on a HiSeq 1500 device (Illumina, San Diego, CA, USA). A mean of 72±17 million passing Illumina quality filter reads was obtained for each of the nine samples.

#### RNASeq bioinformatics analysis

Analyses were performed using the Eoulsan pipeline [67], including read filtering, mapping, alignment filtering, read quantification. Before mapping, reads ⩽40 bases were removed, and reads with quality mean ⩽30 were discarded. Reads were then aligned against the Mus musculus genome (mm10 version Ensembl 75) using Bowtie (version 0.12.9, parameters -n 2 -l 34 -e 70 -k 2 –best) [68]. Alignments from reads matching more than once on the reference genome were removed using Java version of sam tools [69]. To compute gene expression, Mus musculus GFF3 genome annotation version mm10 version75 from Ensembl database was used. All overlapping regions between alignments and referenced genes were counted using HTSeq-count 0.5.3 [70]. The RNASeq gene expression data and raw fastq files are available on the GEO repository (www.ncbi.nlm.nih.gov/geo/) under accession number: GSE7293.

#### Differential gene expression analysis

For the two different sample groups (endfeet vs astrocytes), we assessed which genes displayed statistically significant differential expression using the Bioconductor package DESeq2 (version 1.4.5) written for the R statistical programming environment. DESeq2 assumes the RNA-seq counts are distributed according to negative binomial distributions. It uses generalized linear modelling to test individual null hypotheses of a log_2_ fold changes of zero between conditions for each gene [71]. Pathway analysis was performed as described above.

### Immunohistolabeling and confocal imaging

#### On brain sections

For immunostaining after FISH, slices were rinsed in PBS following the FISH procedure and incubated 1 h at room temperature in the blocking solution (1 mg ml^−1^ bovine serum albumine, 0.2% goat serum, 0.3% Triton X-100 in PBS). For simple immunostaining, cryosections were fixed in PBS/PFA 4% 15 min, rinse in PBS and incubated 1 h at room temperature in the blocking solution (2% goat serum, 0.2% Triton X-100 in PBS). Slices were then incubated in primary antibodies diluted in the blocking solution12 h at 4 °C, rinsed three times 5 min in PBS, incubated secondary antibodies diluted in the blocking solution 2 h at room temperature, rinsed three times 5 min in PBS, and mounted in Fluoromount (Southern Biotech, Birmingham, AL, USA). Images were taken on a SP5 confocal microscope (Leica, Wetzlar, Germany) and deconvoluted with Huygens software (Scientific Volume Imaging b.v., Hilversum, The Netherlands). For Golgi analysis, 3D reconstitution and calculation of length processes were performed using IMOD software [31]. The center of the nucleus was used as the start point to calculate the length of PvAPs and Golgi processes. Only PvAPs with a continuous GFAP labeling were taken into account. The last point of labeling was considered as the end of the Golgi branch when Golgi was discontinuous. Forty four astrocytes in four mice were imaged and analyzed.

#### On purified brain vessels

Purified brain vessels were immobilized on a glass slide coated with Cell Tak (Corning) and immunostaining was performed as described for brain sections.

### Quantitative RT-PCR

Total RNAs were extracted from 2-month-old C57BL6-purified brain vessels submitted or not to basal lamina digestion using the RNeasy Lipid tissue kit (Qiagen). cDNA were synthesized from 1 μg RNA using Reverse Transcriptase Superscript II (Thermo Fisher) and with random primers. cDNA samples were diluted by adding 100 μl of low TE buffer (10 mM Tris; 0.1 mM EDTA; pH=8.0) (TEKnova, Hollister, CA, USA) and stored at −20 °C. PCR were performed in triplicate on a LC480 Roche Light cycler on 2 μl cDNA using the SybrGreen master mix (Roche). Cycling was 50 °C for 2 min, 95 °C for 10 min, and 40 cycles of 95 °C for 15 s and 60 °C for 1 min. The relative abundance of amplified cDNA was calculated as 2^−ΔCt^, where ΔCt (change in cycle threshold) equals Ct in brain vessels minus Ct in digested brain vessels. Results are expressed as means of 2^−ΔCt^ tested cDNA/2^−ΔCt^ RNA18s values. Experiments were done on three independent pool of brain vessels purified from at least four mice. Statistics were done using the Mann–Whitney two-tailed test * stands for *P*=0.05. Mean values are indicated±s.e.m.

Primers: Aqp4 forward 5′- CTTTCTGGAAGGCAGTCTCAG-3′, Aqp4 reverse 5′- CCACACCGAGCAAAACAAAGAT-3′, Gfap forward 5′- GGGGCAAAAGCACCAAAGAAG-3′, Gfap reverse 5′- GGGACAACTTGTATTGTGAGCC-3′, Cldn5 forward 5′- TAAGGCACGGGTAGCACTCA-3′, Cldn5 reverse 5′- GGACAACGATGTTGGCGAAC-3′, Acta2 forward 5′- GTCCCAGACATCAGGGAGTAA-3′, Acta2 reverse 5′- TCGGATACTTCAGCGTCAGGA-3′, RNA18s forward 5′- TTGAAAATCCGGGGGAGAG-3′; RNA 18s reverse 5′- ACATTGTTCCAACATGCCAG-3′.

### Western blot

Purified brain vessels were homogenized in PBS containing 2% SDS and EDTA-free Complete Protease Inhibitor (Roche), sonicated three times at 20 Hz (Vibra cell VCX130) and centrifuged 20 min at 10 000 *g* at 4 °C. Supernatants were boiled in Laemmli loading buffer. Protein content was measured using the Pierce 660 nm protein assay reagent (Thermo Scientific, Waltham, MA, USA). Equal amounts of proteins were separated by denaturing electrophoresis in 4–12% NuPAGE gradient gel (Thermo Fisher) and electrotransfered to nitrocellulose membranes. Membranes were analyzed as previously described [72]. HRP activity was visualized by ECL using Western Lightning plus enhanced chemoluminescence system (Perkin Elmer, Waltham, MA, USA). Chemoluminescence imaging was performed on a LAS4000 (Fujifilm, Minato-ku, Tokyo, Japan). Histone 2a or 3 expression was used as a loading reference. All experiments were done in triplicates (*n*=3). *n* is a pool of brain vessels purified from at least four mice. Statistics were done using the Mann–Whitney two-tailed test * stands for *P*=0.05. Mean values are indicated±s.e.m.

### Electron microscopy

Two-month-old C57BL6 mice were anesthetized with Ketamine/Xylazine (140/8 mg kg^−1^, i.p.) and transcardially perfused with the fixative (2% paraformaldehyde, 3% glutaraldehyde, 3 mM CaCl_2_ in 0.1 M cacodylate buffer pH 7.4) for 12 min. Brains were removed and left overnight at 4 °C in the same fixative. Brain fragments (0.3×1×1 mm^3^) were then postfixed first in 0.1 M cacodylate buffer pH 7.4+1% OsO4 for 1 h at 4 °C and then in 1% aqueous Uranyl Acetate for 2 h at room temperature (RT). After dehydration in graded ethanol, followed by propylene oxide, the fragments were embedded in Epon. Ultrathin (80 nm) sections were prepared, stained in Lead Citrate and photographed in a Jeol 100S transmission electron microscope (Jeol, Croissy-sur-Seine, France) equipped with a 2×2k Orius 830 CCD camera (Roper Scientific, Evry, France). Seventy-eight astrocytes of three mice have been analyzed.

For GM130 immunolocalization, the brain was fixed by transcardial perfusion of the fixative (4% paraformaldehyde, 0.2% glutaraldehyde in 0.1 M phosphate buffer pH 7.4), then removed and fixed for an additional 2 h at 4 °C in 4% paraformaldehyde. Vibratome sections (70 μm) were cryoprotected in 30% sucrose (three changes in 24 h) and frozen/thawn in liquid nitrogen. All subsequent procedures were done at RT. After extensive washing and neutralization of free aldehyde groups in 100 mM Glycine, brain sections were incubated sequentially in 10% goat serum for 2 h, primary mouse anti-GM130 antibody (BD Biosciences Cat # 610822 RRID: AB_398141) (1:250) for 24 h and biotinylated goat anti-mouse secondary antibody (Jackson Immunoresearch, West Grove, PA, USA) (1:1000) for 24 h. Staining was revealed with the Vectastain Elite ABC kit (CliniSciences SAS, Nanterre, France) following manufacturer instruction. Sections were then postfixed first in 2.5% glutaraldehyde for 30 min at RT and then in 0.5% OsO4 for 30 min at 4 °C before being dehydrated and flat-embedded in Epon as detailed in the above section. One mm^2^ squares were cut from embedded sections in the cortex region and glued on an Epon block for sectioning on an ultramicrotome. Ultrathin sections were observed without lead staining.
