# Supplementary material for: Translation in astrocyte distal processes sets molecular heterogeneity at the gliovascular interface
Source: Cell Discov. 2017 Mar 28;3:17005–. doi: 10.1038/celldisc.2017.5 (PMC5368712; doi:10.1038/celldisc.2017.5)
Supplement: Supplementary Figure S1 [file celldisc20175-s1.pdf]

Figure S1

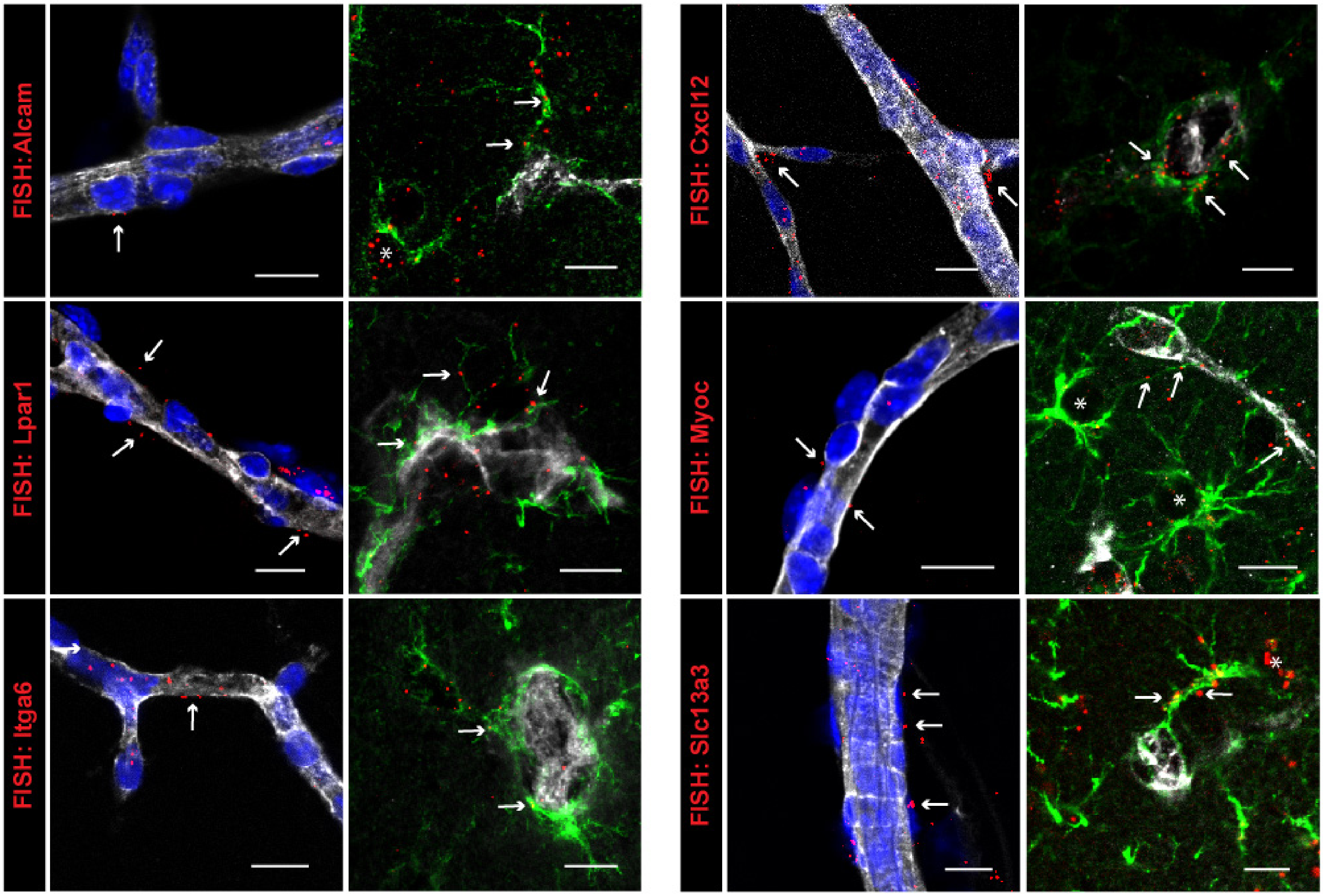

FISH:Alcam
FISH: Lpar1
FISH: Itga6
FISH: Cxcl12
FISH: Myoc
FISH: Slc13a3

Figure S1 mRNAs of the endfeet transcriptome are present in astrocyte perivascular processes and endfeet. Representative confocal images of some endfeet transcriptome transcripts detected by FISH (red) in hippocampus slices and purified brain vessels. The vessel surface is stained with IB4 (gray) and nuclei with Hoechst (blue). The astrocytes are immunostained for GFAP (green). The astrocyte somata are indicated with an asterisk. The white arrows indicate extravascular FISH labeling on the vessels and at the level of GFAP positive filaments in PvAPs and endfeet.
