# Supplementary material for: Translation in astrocyte distal processes sets molecular heterogeneity at the gliovascular interface
Source: Cell Discov. 2017 Mar 28;3:17005–. doi: 10.1038/celldisc.2017.5 (PMC5368712; doi:10.1038/celldisc.2017.5)
Supplement: Supplementary Figure S2 [file celldisc20175-s2.pdf]

Figure S2

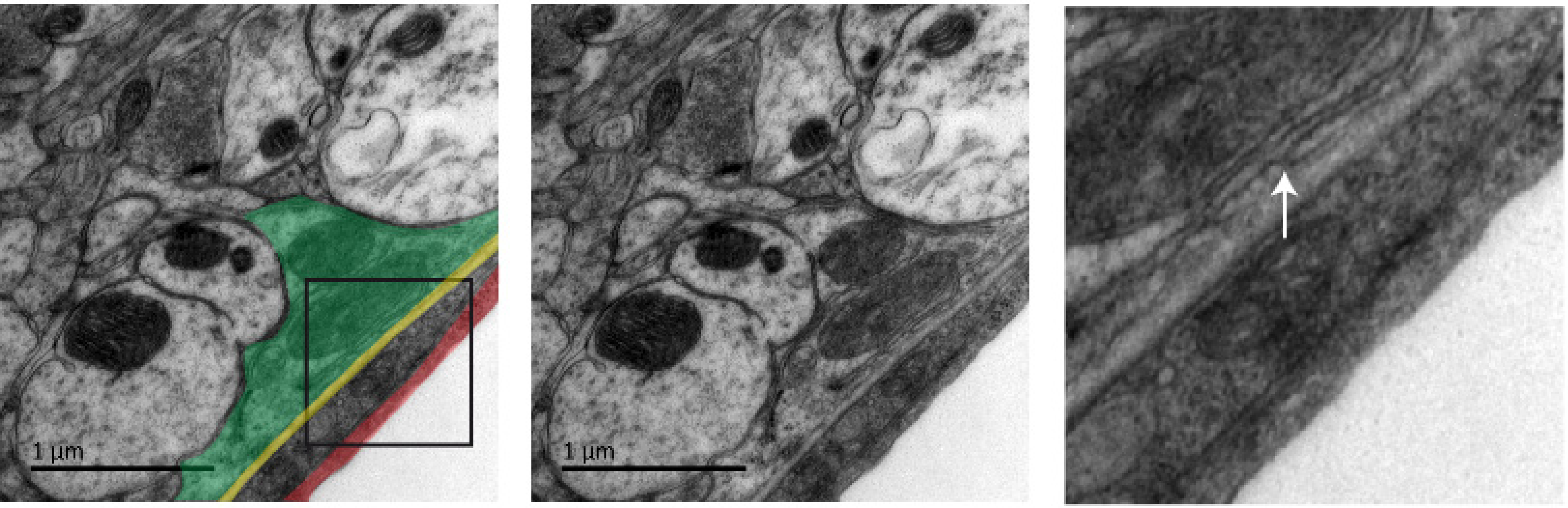

Figure S2 Contact sites between endoplasmic reticulum and plasma membrane in astrocyte endfeet. Representative transmission electron microscopy image of a contact site between endoplasmic reticulum and the astrocyte endfoot plasma membrane. Enlarged view of the squared area shows details of the contact site indicated by the white arrow. On the left image, the different structures of the gliovascular unit are colored: an astrocyte endfoot (green), the basal lamina (yellow), an endothelial cell (red).
