# Supplementary material for: Translation in astrocyte distal processes sets molecular heterogeneity at the gliovascular interface
Source: Cell Discov. 2017 Mar 28;3:17005–. doi: 10.1038/celldisc.2017.5 (PMC5368712; doi:10.1038/celldisc.2017.5)
Supplement: Supplementary Table S1 [file celldisc20175-s3.pdf]

Table S1 The astrocyte endfeet transcriptome. Microarray comparison of mRNAs extracted from brain vessels with or without partial basal lamina (BL) enzymatic digestion. Astrocyte endfeet mRNAs are depleted upon BL digestion with a fold change ≤ -2, $p \leq 0.05$ compared with the undigested vessels.

FAST DB STABLE ID	Gene name	Gene description	Change in expression upon BL digestion	
			Fold Change	p-value
GSMG0022641	Lcn2	lipocalin 2	-7.30	8.5E-03
GSMG0025256	Tmem212	transmembrane protein 212	-7.26	1.8E-04
GSMG0019171	Slc14a2	solute carrier family 14 (urea transporter), membe	-7.12	5.0E-03
GSMG0011339	Dnah12	dynein, axonemal, heavy chain 12	-6.94	2.2E-04
GSMG0037483	Ttc29	tetratricopeptide repeat domain 29	-6.85	4.0E-04
GSMG0011341	Dnah12	dynein, axonemal, heavy chain 12	-6.55	4.1E-05
GSMG0008793	Cdhr3	cadherin-related family member 3	-6.47	2.2E-04
GSMG0007463	Gfap	glial fibrillary acidic protein	-5.19	4.5E-07
GSMG0016200	T2	brachyury 2	-5.07	3.4E-04
GSMG0026519	Ccdc180	coiled-coil domain containing 180	-5.05	4.6E-04
GSMG0013872	Rgs22	regulator of G-protein signalling 22	-5.04	7.7E-05
GSMG0029823	Dnah10	dynein, axonemal, heavy chain 10	-4.91	1.0E-03
GSMG0037721	Lrrc36	leucine rich repeat containing 36	-4.91	1.2E-04
GSMG0018737	Aqp4	aquaporin 4	-4.85	1.0E-04
GSMG0036594	Dnah3	dynein, axonemal, heavy chain 3	-4.82	3.6E-04
GSMG0024721	Spag17	sperm associated antigen 17	-4.80	1.6E-04
GSMG0000970	Myoc	myocilin	-4.79	4.3E-06
GSMG0023304	Bbox1	butyrobetaine (gamma), 2-oxoglutarate dioxygena	-4.76	1.6E-04
GSMG0014419	Adamts20	a disintegrin-like and metallopeptidase (reprolysi	-4.69	1.0E-04
GSMG0028313	Mfsd2a	major facilitator superfamily domain containing 2	-4.65	8.5E-05
GSMG0025440	Ankub1	ankrin repeat and ubiquitin domain containing 1	-4.52	3.6E-05
GSMG0014362	Mlc1	megalencephalic leukoencephalopathy with subco	-4.50	1.5E-05
GSMG0003799	Adgb	androglobin	-4.39	8.3E-07
GSMG0044779	Mir495	microRNA 495	-4.36	6.4E-03
GSMG0016774	Unc5cl	unc-5 homolog C (C. elegans)-like	-4.36	2.0E-04
GSMG0043878	Lrrc71	leucine rich repeat containing 71	-4.27	1.6E-05
GSMG0038237	Got1l1	glutamic-oxaloacetic transaminase 1-like 1	-4.17	2.6E-03
GSMG0029799	Lrrc43	leucine rich repeat containing 43	-4.12	2.8E-04
GSMG0051981	Dthd1	death domain containing 1	-4.05	3.8E-04
GSMG0020090	Ppp1r32	protein phosphatase 1, regulatory subunit 32	-4.03	5.9E-03
GSMG0029792	Wdr66	WD repeat domain 66	-3.99	2.4E-04
GSMG0015617	Atp13a5	ATPase type 13A5	-3.93	1.0E-04
GSMG0011235	Sntn	sentan, cilia apical structure protein	-3.86	1.6E-05
GSMG0003879	Myb	myeloblastosis oncogene	-3.86	8.6E-04
GSMG0033364	Ccdc37	coiled-coil domain containing 37	-3.81	5.4E-04
GSMG0013041	Dnah5	dynein, axonemal, heavy chain 5	-3.75	9.6E-05
GSMG0060452	Cfap46	cilia and flagella associated protein 46	-3.70	4.1E-03
GSMG0026023	---	---	-3.70	1.1E-03
GSMG0051586	Ankrd66	ankyrin repeat domain 66	-3.65	3.0E-04
GSMG0004063	---	---	-3.56	4.4E-04
GSMG0024302	Stoml3	stomatin (Epb7.2)-like 3	-3.52	4.4E-03
GSMG0022225	---	---	-3.51	1.2E-03
GSMG0001578	Dnah7a	dynein, axonemal, heavy chain 7A	-3.50	5.2E-04
GSMG0039010	Slc7a5	solute carrier family 7 (cationic amino acid transp	-3.49	1.8E-03
GSMG0016942	Vit	vitrin	-3.48	1.2E-03
GSMG0003014	Fabp7	fatty acid binding protein 7, brain	-3.46	1.0E-04
GSMG0051950	Gm12695	predicted gene 12695	-3.46	3.8E-04
GSMG0044780	Mir667	microRNA 667	-3.45	4.4E-04

GSMG0014361	Ttll8	tubulin tyrosine ligase-like family, member 8	-3.43	2.0E-04
GSMG0017406	Angptl4	angiopoietin-like 4	-3.41	0.03
GSMG0018390	Zfp474	zinc finger protein 474	-3.40	2.6E-04
GSMG0002213	Hmcn1	hemicentin 1	-3.38	2.1E-03
GSMG0030218	Fbxl13	F-box and leucine-rich repeat protein 13	-3.35	1.6E-04
GSMG0027852	Lpar1	lysophosphatidic acid receptor 1	-3.29	2.0E-03
GSMG0013758	Slc1a3	solute carrier family 1 (glial high affinity glutama	-3.28	1.1E-03
GSMG0032429	Slc6a13	solute carrier family 6 (neurotransmitter transport	-3.26	8.2E-04
GSMG0026471	Car9	carbonic anhydrase 9	-3.26	3.2E-04
GSMG0006073	Kcnj16	potassium inwardly-rectifying channel, subfamily	-3.22	1.0E-03
GSMG0032007	Ndnf	neuron-derived neurotrophic factor	-3.21	6.0E-04
GSMG0034090	Pglyrp1	peptidoglycan recognition protein 1	-3.18	2.5E-05
GSMG0001823	Slc19a3	solute carrier family 19, member 3	-3.18	1.7E-03
GSMG0016909	Fam179a	family with sequence similarity 179, member A	-3.18	1.9E-05
GSMG0050826	Iqca	IQ motif containing with AAA domain	-3.17	1.8E-03
GSMG0037632	Mt2	metallothionein 2	-3.15	3.0E-04
GSMG0022070	Nnat	neuronatin	-3.09	3.6E-04
GSMG0025365	Slc7a11	solute carrier family 7 (cationic amino acid transp	-3.08	1.6E-03
GSMG0050889	Dnah1	dynein, axonemal, heavy chain 1	-3.08	1.5E-03
GSMG0014377	Odf3b	outer dense fiber of sperm tails 3B	-3.08	5.6E-05
GSMG0023891	Slc13a3	solute carrier family 13 (sodium-dependent dicart	-3.05	6.6E-04
GSMG0044790	Mir154	microRNA 154	-3.04	4.0E-04
GSMG0016452	Cdkn1a	cyclin-dependent kinase inhibitor 1A (P21)	-3.03	0.05
GSMG0044785	Mir300	microRNA 300	-3.03	2.4E-03
GSMG0017852	Xdh	xanthine dehydrogenase	-3.03	4.0E-03
GSMG0021475	Slc1a2	solute carrier family 1 (glial high affinity glutama	-3.01	4.5E-03
GSMG0004264	Gm867	predicted gene 867	-3.00	2.0E-05
GSMG0019565	Slc1a1	solute carrier family 1 (neuronal/epithelial high a	-2.99	2.4E-04
GSMG0008482	Serpina3n	serine (or cysteine) peptidase inhibitor, clade A, r	-2.99	0.04
GSMG0014385	Syt10	synaptotagmin X	-2.99	1.3E-03
GSMG0005688	Car4	carbonic anhydrase 4	-2.98	4.2E-03
GSMG0051138	Gm17455	predicted gene, 17455	-2.98	3.4E-04
GSMG0060093	Ranbp3l	RAN binding protein 3-like	-2.98	3.2E-04
GSMG0032380	Cxcl12	chemokine (C-X-C motif) ligand 12	-2.96	5.1E-03
GSMG0019363	Slc22a6	solute carrier family 22 (organic anion transporter	-2.95	2.7E-03
GSMG0051302	Gm11710	predicted gene 11710	-2.95	1.2E-04
GSMG0052477	Ccdc153	coiled-coil domain containing 153	-2.94	1.1E-03
GSMG0037397	Pde4c	phosphodiesterase 4C, cAMP specific	-2.94	1.2E-04
GSMG0039058	Agt	angiotensinogen (serpin peptidase inhibitor, clade	-2.92	6.1E-07
GSMG0028856	Sema3c	sema domain, immunoglobulin domain (Ig), short	-2.91	4.2E-04
GSMG0012462	Gjb2	gap junction protein, beta 2	-2.88	3.9E-03
GSMG0034319	Dmkn	dermokine	-2.87	5.2E-03
GSMG0012464	Gjb6	gap junction protein, beta 6	-2.82	2.0E-04
GSMG0022873	Dpp4	dipeptidylpeptidase 4	-2.82	6.8E-04
GSMG0028587	Gm694	predicted gene 694	-2.81	2.4E-04
GSMG0012697	Fam216b	family with sequence similarity 216, member B	-2.81	4.6E-04
GSMG0044787	Mir487b	microRNA 487b	-2.81	2.0E-03
GSMG0042749	930519F16Ri	RIKEN cDNA 4930519F16 gene	-2.80	1.4E-03
GSMG0033855	Abcc9	ATP-binding cassette, sub-family C (CFTR/MRP	-2.80	4.0E-04
GSMG0039347	Hepacam	hepatocyte cell adhesion molecule	-2.80	1.4E-04
GSMG0051938	Mir3095	microRNA 3095	-2.80	8.4E-04
GSMG0043531	Ttll3	tubulin tyrosine ligase-like family, member 3	-2.78	6.4E-05
GSMG0021209	Itga6	integrin alpha 6	-2.77	2.4E-03

GSMG0026602	Musk	muscle, skeletal, receptor tyrosine kinase	-2.76	2.9E-03
GSMG0001167	Dnah14	dynein, axonemal, heavy chain 14	-2.76	5.0E-03
GSMG0051084	Col6a5	collagen, type VI, alpha 5	-2.73	1.1E-03
GSMG0036523	Lyve1	lymphatic vessel endothelial hyaluronan receptor	-2.72	0.04
GSMG0011342	Asb14	ankyrin repeat and SOCS box-containing 14	-2.71	2.0E-05
GSMG0032430	Slc6a12	solute carrier family 6 (neurotransmitter transport	-2.70	3.9E-03
GSMG0014892	Tfrc	transferrin receptor	-2.70	4.2E-04
GSMG0028755	Vwa1	von Willebrand factor A domain containing 1	-2.70	5.2E-04
GSMG0033882	ntd1 // Gm38	lamin tail domain containing 1 // predicted gene,	-2.69	2.5E-03
GSMG0004529	Tmtc2	transmembrane and tetratricopeptide repeat conta	-2.69	1.2E-03
GSMG0001548	Slc40a1	solute carrier family 40 (iron-regulated transporte	-2.69	2.2E-03
GSMG0003006	Gja1	gap junction protein, alpha 1	-2.68	7.4E-04
GSMG0004468	Cfap54	cilia and flagella associated protein 54	-2.68	7.6E-05
GSMG0016230	Slc22a2	solute carrier family 22 (organic cation transporte	-2.67	8.8E-03
GSMG0016709	Tcte1	t-complex-associated testis expressed 1	-2.66	1.6E-03
GSMG0024713	Hmgcs2	3-hydroxy-3-methylglutaryl-Coenzyme A syntha	-2.65	0.01
GSMG0002127	Gpr37l1	G protein-coupled receptor 37-like 1	-2.65	1.7E-03
GSMG0000688	Sctr	secretin receptor	-2.64	5.2E-04
GSMG0006713	Fam183b	family with sequence similarity 183, member B	-2.64	1.5E-03
GSMG0004156	Tspan15	tetraspanin 15	-2.63	1.0E-04
GSMG0042422	Akap14	A kinase (PRKA) anchor protein 14	-2.63	1.0E-04
GSMG0037996	930567H12Ri	RIKEN cDNA 4930567H12 gene	-2.63	6.1E-05
GSMG0040835	Ccdc33	coiled-coil domain containing 33	-2.63	2.4E-03
GSMG0017434	C4b	complement component 4B (Chido blood group)	-2.63	7.6E-05
GSMG0029424	Art3	ADP-ribosyltransferase 3	-2.63	3.1E-03
GSMG0022489	Enkur	enkurin, TRPC channel interacting protein	-2.61	5.6E-04
GSMG0024772	Slc16a1	solute carrier family 16 (monocarboxylic acid tra	-2.61	4.3E-03
GSMG0006181	Ccdc40	coiled-coil domain containing 40	-2.61	3.4E-04
GSMG0032378	430408G22Ri	RIKEN cDNA 8430408G22 gene	-2.61	0.01
GSMG0002682	Syne1	spectrin repeat containing, nuclear envelope 1	-2.60	3.2E-04
GSMG0010761	Ctla2b	cytotoxic T lymphocyte-associated protein 2 beta	-2.59	3.6E-03
GSMG0002002	Cfap221	cilia and flagella associated protein 221	-2.59	3.0E-03
GSMG0028654	Ptchd2	patched domain containing 2	-2.59	3.8E-04
GSMG0005819	Ttll6	tubulin tyrosine ligase-like family, member 6	-2.59	7.0E-04
GSMG0001924	St8sia4	ST8 alpha-N-acetyl-neuraminide alpha-2,8-sialyl	-2.58	9.0E-04
GSMG0044751	Mir493	microRNA 493	-2.58	5.1E-03
GSMG0037095	Plat	plasminogen activator, tissue	-2.58	1.0E-04
GSMG0040487	Dpy19l2	dpy-19-like 2 (C. elegans)	-2.56	1.2E-04
GSMG0044773	Mir329	microRNA 329	-2.55	2.4E-03
GSMG0052074	Apold1	apolipoprotein L domain containing 1	-2.55	0.04
GSMG0021175	Nostrin	nitric oxide synthase trafficker	-2.55	5.0E-03
GSMG0036249	Ccdc81	coiled-coil domain containing 81	-2.55	4.3E-03
GSMG0003043	ock2 // Mir69	sparc/osteonectin, cwcv and kazal-like domains p	-2.54	1.2E-03
GSMG0008163	Lrrc9	leucine rich repeat containing 9	-2.54	1.2E-04
GSMG0020688	Mrc1	mannose receptor, C type 1	-2.54	5.5E-03
GSMG0016696	Clic5	chloride intracellular channel 5	-2.53	2.3E-03
GSMG0037757	Cdh1	cadherin 1	-2.51	2.8E-04
GSMG0003846	Ect2l	epithelial cell transforming sequence 2 oncogene-	-2.50	5.2E-04
GSMG0037811	Hydin	HYDIN, axonemal central pair apparatus protein	-2.50	4.1E-03
GSMG0010552	F13a1	coagulation factor XIII, A1 subunit	-2.49	0.03
GSMG0015187	Robo1	roundabout homolog 1 (Drosophila)	-2.49	0.02
GSMG0043909	Mir128-1	microRNA 128-1	-2.48	9.8E-04
GSMG0007834	Osr1	odd-skipped related 1 (Drosophila)	-2.48	1.6E-04

GSMG0018589	Mro	maestro	-2.48	3.7E-03
GSMG0051045	Dchs1	dachsous 1 (Drosophila)	-2.47	1.4E-04
GSMG0000497	933407L21Ri	RIKEN cDNA 4933407L21 gene	-2.47	4.2E-04
GSMG0023455	Gatm	glycine amidinotransferase (L-arginine:glycine ar	-2.47	7.2E-04
GSMG0014730	Serpind1	serine (or cysteine) peptidase inhibitor, clade D, r	-2.46	5.1E-03
GSMG0039450	Mpzl2	myelin protein zero-like 2	-2.46	3.1E-03
GSMG0053143	310047C21Ri	RIKEN cDNA 2810047C21 gene 1	-2.45	0.01
GSMG0010972	---	---	-2.45	1.8E-04
GSMG0046912	Mir126a	microRNA 126a	-2.45	1.6E-03
GSMG0060409	/ Mirlet7c-2 //	microRNA let7b // microRNA let7c-2 // long inte	-2.44	1.2E-04
GSMG0046639	Mir143	microRNA 143	-2.44	2.6E-04
GSMG0050812	Mir384	microRNA 384	-2.44	3.1E-03
GSMG0008310	Acot6	acyl-CoA thioesterase 6	-2.43	8.8E-04
GSMG0048759	Mir29a	microRNA 29a	-2.43	0.02
GSMG0044784	Mir376a	microRNA 376a	-2.42	5.3E-03
GSMG0002569	Ccdc121	coiled-coil domain containing 121	-2.42	5.8E-04
GSMG0030671	Tecrl	trans-2,3-enoyl-CoA reductase-like	-2.41	9.2E-03
GSMG0011998	930594M22R	RIKEN cDNA 4930594M22 gene	-2.41	5.0E-04
GSMG0032414	Slc25a18	solute carrier family 25 (mitochondrial carrier), m	-2.41	3.8E-04
GSMG0006878	// Tnfsf13 // T	tumor necrosis factor (ligand) superfamily, memb	-2.41	1.2E-04
GSMG0016933	Rasgrp3	RAS, guanyl releasing protein 3	-2.40	3.8E-04
GSMG0044758	Mir136	microRNA 136	-2.40	1.7E-05
GSMG0039844	Cd109	CD109 antigen	-2.39	1.0E-03
GSMG0021474	Pamr1	peptidase domain containing associated with mus	-2.39	1.7E-03
GSMG0051287	Gm11517	predicted gene 11517	-2.37	1.2E-03
GSMG0024736	Casq2	calsequestrin 2	-2.37	2.4E-04
GSMG0028528	C1qa	complement component 1, q subcomponent, alpha	-2.37	2.8E-03
GSMG0039772	Aldh1a2	aldehyde dehydrogenase family 1, subfamily A2	-2.36	6.9E-03
GSMG0027725	Fam166b	family with sequence similarity 166, member B	-2.36	1.1E-03
GSMG0020654	Itih5	inter-alpha (globulin) inhibitor H5	-2.35	6.1E-05
GSMG0007222	Ankfn1	ankyrin-repeat and fibronectin type III domain co	-2.35	1.4E-04
GSMG0006346	Ddc	dopa decarboxylase	-2.35	3.7E-03
GSMG0041467	Hhatl	hedgehog acyltransferase-like	-2.34	0.02
GSMG0035929	Abcc6	ATP-binding cassette, sub-family C (CFTR/MRP	-2.34	3.3E-03
GSMG0015505	Lrrc74b	leucine rich repeat containing 74B	-2.34	7.8E-03
GSMG0000301	Gm973	predicted gene 973	-2.34	5.8E-05
GSMG0027960	---	---	-2.34	0.03
GSMG0000844	ik // Mir181b	RIKEN cDNA A430106G13 gene // microRNA 1	-2.34	8.6E-04
GSMG0032868	Fam180a	family with sequence similarity 180, member A	-2.34	9.9E-03
GSMG0001064	Kcnj10	potassium inwardly-rectifying channel, subfamily	-2.34	2.0E-03
GSMG0007750	Notum	notum pectinacetylesterase homolog (Drosophila)	-2.33	2.2E-04
GSMG0023305	Fibin	fin bud initiation factor homolog (zebrafish)	-2.33	1.4E-03
GSMG0006982	Serpinf1	serine (or cysteine) peptidase inhibitor, clade F, m	-2.33	1.5E-03
GSMG0041058	Col12a1	collagen, type XII, alpha 1	-2.32	8.4E-03
GSMG0020116	Ms4a7	membrane-spanning 4-domains, subfamily A, me	-2.32	6.8E-03
GSMG0022384	Gm10115	predicted gene 10115	-2.32	9.4E-03
GSMG0032992	Rarres2	retinoic acid receptor responder (tazarotene induc	-2.31	1.9E-03
GSMG0036628	Zkscan2	zinc finger with KRAB and SCAN domains 2	-2.31	3.1E-03
GSMG0023972	Gm10714	predicted gene 10714	-2.31	1.0E-03
GSMG0007856	Ntsr2	neurotensin receptor 2	-2.31	1.3E-03
GSMG0012711	Rgcc	regulator of cell cycle	-2.31	2.6E-04
GSMG0012802	---	---	-2.30	1.3E-03
GSMG0028816	Sema3d	sema domain, immunoglobulin domain (Ig), short	-2.30	1.7E-03

GSMG0036314	Slco2b1	solute carrier organic anion transporter family, m	-2.28	7.0E-04
GSMG0016842	Adgre1	adhesion G protein-coupled receptor E1	-2.28	9.7E-05
GSMG0042913	Il13ra2	interleukin 13 receptor, alpha 2	-2.28	6.4E-03
GSMG0017879	Cyp1b1	cytochrome P450, family 1, subfamily b, polypep	-2.28	2.2E-03
GSMG0032993	Gimap6	GTPase, IMAP family member 6	-2.28	5.7E-03
GSMG0022383	Gm10855	predicted gene 10855	-2.27	0.03
GSMG0043690	Cfap54	cilia and flagella associated protein 54	-2.27	0.02
GSMG0022407	Itih2	inter-alpha trypsin inhibitor, heavy chain 2	-2.27	4.4E-04
GSMG0006671	Slc22a4	solute carrier family 22 (organic cation transporte	-2.27	1.6E-03
GSMG0042279	Gpm6b	glycoprotein m6b	-2.27	3.2E-03
GSMG0044778	Mir543	microRNA 543	-2.26	8.6E-04
GSMG0001656	Dytn	dystrotelin	-2.26	5.3E-03
GSMG0007220	932411E22Ri	RIKEN cDNA 4932411E22 gene	-2.26	2.2E-03
GSMG0030435	Fgfbp1	fibroblast growth factor binding protein 1	-2.26	0.03
GSMG0018047	---	---	-2.25	6.2E-05
GSMG0007316	Plxdc1	plexin domain containing 1	-2.24	3.4E-03
GSMG0035146	---	---	-2.24	5.1E-03
GSMG0001550	Slc39a10	solute carrier family 39 (zinc transporter), membe	-2.23	2.0E-03
GSMG0012264	---	---	-2.23	6.3E-03
GSMG0017486	H2-T24	histocompatibility 2, T region locus 24	-2.23	2.8E-04
GSMG0043694	Slc26a10	solute carrier family 26, member 10	-2.22	0.04
GSMG0005598	Vtn	vitronectin	-2.22	3.2E-03
GSMG0012607	930012K11Ri	RIKEN cDNA 9930012K11 gene	-2.22	2.3E-05
GSMG0000880	Ivns1abp	influenza virus NS1A binding protein	-2.22	2.2E-03
GSMG0030250	---	---	-2.21	1.3E-03
GSMG0012801	Tbc1d4	TBC1 domain family, member 4	-2.21	1.0E-04
GSMG0003342	Rfx4	regulatory factor X, 4 (influences HLA class II ex	-2.21	1.3E-03
GSMG0000737	Gm16083	predicted gene 16083	-2.21	4.4E-03
GSMG0032382	Gm9946	predicted gene 9946	-2.21	1.9E-03
GSMG0034878	P4ha3	procollagen-proline, 2-oxoglutarate 4-dioxygenas	-2.21	1.6E-04
GSMG0000283	Aox3	aldehyde oxidase 3	-2.20	0.01
GSMG0029801	B3gnt4	UDP-GlcNAc:betaGal beta-1,3-N-acetylglucosan	-2.20	7.9E-05
GSMG0041026	Scg3	secretogranin III	-2.19	6.2E-04
GSMG0060610	Mir361	microRNA 361	-2.18	1.7E-03
GSMG0052118	Vmn2r40	vomeronasal 2, receptor 40	-2.18	3.5E-03
GSMG0001242	Slc30a1	solute carrier family 30 (zinc transporter), membe	-2.18	0.01
GSMG0031567	Ptprz1	protein tyrosine phosphatase, receptor type Z, pol	-2.18	2.7E-03
GSMG0008754	Rsad2	radical S-adenosyl methionine domain containing	-2.18	1.2E-03
GSMG0004219	930033H14R	RIKEN cDNA A930033H14 gene	-2.17	1.5E-05
GSMG0035489	Vmn2r30	vomeronasal 2, receptor 30	-2.17	1.5E-03
GSMG0040565	Gm3428	predicted gene 3428	-2.17	9.2E-03
GSMG0052934	Mir5128	microRNA 5128	-2.17	3.5E-03
GSMG0023658	Thbd	thrombomodulin	-2.17	8.0E-05
GSMG0033399	Adamts9	a disintegrin-like and metallopeptidase (reprolysi	-2.16	6.4E-04
GSMG0013475	Ribc2	RIB43A domain with coiled-coils 2	-2.16	1.9E-05
GSMG0032877	Ptn	pleiotrophin	-2.16	4.2E-04
GSMG0039538	Slc35f2	solute carrier family 35, member F2	-2.16	4.8E-03
GSMG0004469	Cfap54	cilia and flagella associated protein 54	-2.15	0.02
GSMG0036788	---	---	-2.15	3.7E-03
GSMG0016992	Plekhh2	pleckstrin homology domain containing, family H	-2.15	2.4E-04
GSMG0033889	Itpr2	inositol 1,4,5-triphosphate receptor 2	-2.15	2.0E-03
GSMG0036575	Tmc7	transmembrane channel-like gene family 7	-2.15	6.9E-03
GSMG0008499	Ak7	adenylate kinase 7	-2.15	2.8E-03

GSMG0016386	Ccdc78	coiled-coil domain containing 78	-2.14	8.3E-05
GSMG0008538	---	---	-2.14	5.3E-03
GSMG0031304	Mmd2	monocyte to macrophage differentiation-associate	-2.14	2.2E-03
GSMG0022339	Meig1	meiosis expressed gene 1	-2.14	0.01
GSMG0000789	Fmod	fibromodulin	-2.14	6.4E-03
GSMG0015810	Alcam	activated leukocyte cell adhesion molecule	-2.13	5.6E-04
GSMG0052703	Lyz2	lysozyme 2	-2.13	1.3E-03
GSMG0003870	Pde7b	phosphodiesterase 7B	-2.12	1.6E-03
GSMG0029252	Gm15478	predicted gene 15478	-2.12	0.01
GSMG0000461	Acsl3 // Utp14	acyl-CoA synthetase long-chain family member 3	-2.12	3.5E-05
GSMG0031384	Slc7a1	solute carrier family 7 (cationic amino acid transp	-2.12	1.3E-03
GSMG0035491	Gm3912	predicted gene 3912	-2.11	0.02
GSMG0018479	Apcdd1	adenomatosis polyposis coli down-regulated 1	-2.11	1.0E-04
GSMG0003452	Dcn	decorin	-2.11	7.4E-04
GSMG0014978	Igsf11	immunoglobulin superfamily, member 11	-2.11	7.6E-04
GSMG0006040	810010H24Ri	RIKEN cDNA 1810010H24 gene	-2.10	1.4E-03
GSMG0050947	Kif6	kinesin family member 6	-2.10	2.7E-03
GSMG0043689	Cfap54	cilia and flagella associated protein 54	-2.10	2.1E-03
GSMG0032268	---	---	-2.10	1.3E-03
GSMG0013994	Gm16006	predicted gene 16006	-2.10	0.02
GSMG0036508	AA474408	expressed sequence AA474408	-2.10	5.9E-03
GSMG0040282	Ccr9	chemokine (C-C motif) receptor 9	-2.10	0.01
GSMG0007643	---	---	-2.09	2.7E-03
GSMG0016872	Lama1	laminin, alpha 1	-2.09	9.8E-04
GSMG0034268	Rasgrp4	RAS guanyl releasing protein 4	-2.09	4.8E-03
GSMG0038486	Cpe	carboxypeptidase E	-2.08	2.0E-04
GSMG0060428	2 // Palm2 // I	A kinase (PRKA) anchor protein 2 // paralemmin	-2.08	5.0E-04
GSMG0046003	ora34 // Mir12	small nucleolar RNA, H/ACA box 34 // microRN	-2.08	2.2E-03
GSMG0029384	Slc4a4	solute carrier family 4 (anion exchanger), membe	-2.08	5.8E-04
GSMG0036120	St8sia2	ST8 alpha-N-acetyl-neuraminide alpha-2,8-sialylt	-2.07	3.8E-04
GSMG0002211	Prg4	proteoglycan 4 (megakaryocyte stimulating factor	-2.07	8.1E-03
GSMG0025609	Bcan	brevican	-2.07	1.6E-04
GSMG0036161	Anpep	alanyl (membrane) aminopeptidase	-2.07	1.4E-03
GSMG0025453	4 // F630111L	purinergic receptor P2Y, G-protein coupled, 14 //	-2.07	0.02
GSMG0010630	Mirlet7d	microRNA let7d	-2.07	2.8E-03
GSMG0044771	Mir323	microRNA 323	-2.07	6.3E-03
GSMG0027304	Padi2	peptidyl arginine deiminase, type II	-2.06	2.5E-05
GSMG0046877	Mir194-2	microRNA 194-2	-2.06	1.4E-03
GSMG0029465	700007G11Ri	RIKEN cDNA 1700007G11 gene	-2.06	6.3E-03
GSMG0016523	Kank3	KN motif and ankyrin repeat domains 3	-2.06	1.4E-03
GSMG0025512	Bche	butyrylcholinesterase	-2.06	0.01
GSMG0060465	---	---	-2.05	0.04
GSMG0001197	Slc30a10	solute carrier family 30, member 10	-2.05	5.8E-04
GSMG0028527	C1qc	complement component 1, q subcomponent, C ch	-2.05	0.03
GSMG0033878	Casc1	cancer susceptibility candidate 1	-2.05	8.5E-03
GSMG0010971	Pde8b	phosphodiesterase 8B	-2.05	4.8E-04
GSMG0008227	---	---	-2.05	1.2E-03
GSMG0045400	Mir18	microRNA 18	-2.04	4.4E-05
GSMG0021390	Lrp4	low density lipoprotein receptor-related protein 4	-2.04	1.8E-04
GSMG0042268	Ace2	angiotensin I converting enzyme (peptidyl-dipept	-2.04	3.0E-03
GSMG0014261	)730005E14R	RIKEN cDNA D730005E14 gene	-2.04	1.5E-03
GSMG0000904	Teddm1a	transmembrane epididymal protein 1A	-2.04	0.02
GSMG0011585	Mhrt	myosin heavy chain associated RNA transcript	-2.04	0.01

GSMG0019619	Ifit2	interferon-induced protein with tetratricopeptide r	-2.03	7.8E-04
GSMG0025068	Ddah1	dimethylarginine dimethylaminohydrolase 1	-2.03	1.6E-03
GSMG0029510	Spp1	secreted phosphoprotein 1	-2.03	2.3E-03
GSMG0041491	Slc6a20a	solute carrier family 6 (neurotransmitter transport	-2.03	2.2E-03
GSMG0012715	Lect1	leukocyte cell derived chemotaxin 1	-2.02	5.0E-04
GSMG0023086	Olfr1046	olfactory receptor 1046	-2.02	3.2E-03
GSMG0026189	Ptgfr	prostaglandin F receptor	-2.02	2.6E-03
GSMG0010763	Ctla2a	cytotoxic T lymphocyte-associated protein 2 alph	-2.02	7.0E-03
GSMG0006088	Ttyh2	tweety homolog 2 (Drosophila)	-2.02	2.0E-03
GSMG0051009	Dnah6	dynein, axonemal, heavy chain 6	-2.02	7.8E-03
GSMG0002916	Ppil6	peptidylprolyl isomerase (cyclophilin)-like 6	-2.02	0.04
GSMG0049845	Mir27a	microRNA 27a	-2.01	0.01
GSMG0030436	Prom1	prominin 1	-2.01	6.4E-03
GSMG0019501	030003E18Ri	RIKEN cDNA E030003E18 gene	-2.01	1.5E-03
GSMG0000484	Daw1	dynein assembly factor with WDR repeat domain	-2.01	0.02
GSMG0019028	Afap1l1	actin filament associated protein 1-like 1	-2.01	0.02
GSMG0019503	Aldh1a1	aldehyde dehydrogenase family 1, subfamily A1	-2.01	2.8E-04
GSMG0028769	Isg15	ISG15 ubiquitin-like modifier	-2.01	3.7E-03
GSMG0039354	Vsig2	V-set and immunoglobulin domain containing 2	-2.01	2.5E-03
GSMG0060569	Mir669n	microRNA 669n	-2.01	2.1E-03
GSMG0026052	Enpep	glutamyl aminopeptidase	-2.01	7.2E-04
GSMG0014129	Ly6c2	lymphocyte antigen 6 complex, locus C2	-2.00	2.8E-03
GSMG0028766	Ttll10	tubulin tyrosine ligase-like family, member 10	-2.00	1.3E-03
GSMG0052660	Ripply1	ripply1 homolog (zebrafish)	-2.00	0.03
