# Supplementary material for: Translation in astrocyte distal processes sets molecular heterogeneity at the gliovascular interface
Source: Cell Discov. 2017 Mar 28;3:17005–. doi: 10.1038/celldisc.2017.5 (PMC5368712; doi:10.1038/celldisc.2017.5)
Supplement: Supplementary Table S2 [file celldisc20175-s4.pdf]

Table S2 Gene Ontology analysis of the astrocyte endfeet transcriptome. The "cellular component" and "biological process" GO pathways significantly depleted upon BL digestion are grouped by cellular compartment (cytoplasm, cytoskeleton, extracellular and membrane) and functions (development, extracellular matrix, metabolism, microtubule, signalisation and transport/homeostasis). Number of entities, the number of transcripts referenced in the pathway; number of measured entities, the number of transcripts found in the transcriptome; Median FC, median change of expression upon BL digestion. A negative score indicates a depletion upon digestion, thus an enrichment of the pathway in the endfeet.

Term type	GO Term	Number of entities	Number of measured entities	Median FC	p-value	Category
Cellular component	apical part of cell	136	13	-2.0	8.62E-04	Cytoplasme
	cell projection	575	32	-2.6	1.37E-03	
	cell projection part	170	14	-3.0	1.99E-03	
	microtubule cytoskeleton	450	15	-3.1	0	Cytoskeleton
	cytoskeletal part	774	18	-2.9	0	
	microtubule	240	8	-3.6	0	
	axoneme	37	9	-3.8	1.16E-05	
	dynein complex	34	7	-3.8	4.82E-04	
	cilium	135	13	-3.4	8.07E-04	
	cilium axoneme	23	5	-3.4	4.61E-03	
	axonemal dynein complex	6	3	-3.8	1.26E-02	
	microtubule associated complex	76	7	-3.8	2.72E-02	
	cilium part	41	5	-3.4	3.45E-02	
	axoneme part	10	3	-3.8	3.49E-02	
	cell surface	305	14	-2.3	0	Extracellular
	extracellular matrix part	92	5	-2.7	0	
	extracellular region	1680	91	-2.2	3.09E-07	
	extracellular region part	774	50	-2.2	4.09E-06	
	extracellular matrix	309	22	-2.2	4.82E-04	
	extracellular space	511	32	-2.3	8.84E-04	
	proteinaceous extracellular matrix	297	20	-2.2	1.78E-03	
	connexon complex	19	3	-2.8	0	Membrane
	plasma membrane	2906	125	-2.1	1.88E-04	
	plasma membrane part	1633	71	-2.0	8.53E-03	
	apical plasma membrane	99	9	-2.0	1.01E-02	
	integral to plasma membrane	531	25	-2.0	3.29E-02	
	gap junction	27	4	-2.8	4.68E-02	
	intrinsic to plasma membrane	552	25	-2.0	4.82E-02	

Term type	GO Term	Number of entities	Number of measured entities	Median FC	p-value	Category
Biological process	nervous system development	854	19	-2.5	0	Development
	anatomical structure morphogenesis	1114	27	-2.5	0	
	system development	2027	40	-2.4	0	
	anatomical structure development	2170	42	-2.4	0	
	developmental process	2743	50	-2.4	0	
	blood vessel development	244	10	-2.4	0	
	multicellular organismal development	2521	46	-2.4	0	
	cell adhesion	561	31	-2.1	9.40E-04	Extracellular matrix/adhesion
	positive regulation of cell-substrate adhesion	27	6	-2.4	9.50E-04	
	biological adhesion	562	31	-2.1	9.64E-04	
	positive regulation of cell adhesion	43	7	-2.5	1.40E-03	
	regulation of cell adhesion	94	10	-2.2	1.59E-03	
	extracellular matrix organization	101	10	-2.6	2.62E-03	
	extracellular structure organization	149	12	-2.6	4.08E-03	
	regulation of cell-substrate adhesion	40	6	-2.4	5.69E-03	
	chondroitin sulfate metabolic process	14	3	-2.5	0	Metabolism
	chondroitin sulfate proteoglycan metabolic proce	16	3	-2.5	0	
	glycosaminoglycan metabolic process	40	7	-2.5	9.50E-04	
	aminoglycan metabolic process	52	7	-2.5	3.76E-03	
	amine metabolic process	336	23	-2.3	3.27E-04	
	polysaccharide metabolic process	92	9	-2.4	5.18E-03	
	hormone metabolic process	90	8	-2.0	1.55E-02	
	isoprenoid biosynthetic process	22	4	-2.2	2.47E-02	
	vitamin metabolic process	69	6	-2.1	4.95E-02	
	cellular amino acid derivative biosynthetic proce	48	5	-2.5	4.97E-02	
	cellular amino acid derivative metabolic process	141	6	-2.4	0	
	glycoprotein metabolic process	147	6	-2.4	0	
	protein polyglycylation	3	3	-2.8	2.46E-03	
	peptide cross-linking	17	4	-2.5	1.21E-02	
	peptide cross-linking via chondroitin 4-sulfate g	8	3	-2.5	2.08E-02	
	microtubule-based movement	101	9	-3.5	8.99E-03	Microtubule
	microtubule-based process	211	12	-2.9	4.36E-02	
	cellular response to extracellular stimulus	45	5	-2.8	4.07E-02	Signalisation
	response to external stimulus	640	29	-2.4	1.99E-02	
	regulation of hormone levels	128	9	-2.0	3.27E-02	
	response to extracellular stimulus	133	9	-2.5	3.96E-02	
	response to wounding	347	17	-2.4	4.59E-02	
	positive regulation of cell communication	189	7	-2.9	0	
	response to stimulus	2437	48	-2.5	0	
	positive regulation of biological process	1493	33	-2.4	0	
	immune effector process	126	8	-2.4	0	
	immune response	471	15	-2.4	0	
	carboxylic acid transport	99	16	-2.1	1.54E-07	Transport/homeostasis of small molecules
	organic acid transport	100	16	-2.1	1.76E-07	
	amino acid transport	71	12	-2.1	5.67E-06	
	organic anion transport	39	9	-2.3	1.29E-05	
	amine transport	92	12	-2.1	6.90E-05	
	dicarboxylic acid transport	11	4	-3.0	3.35E-03	
	metal ion transport	442	24	-2.0	5.08E-03	
	transition metal ion transport	70	7	-2.1	1.57E-02	
	cellular zinc ion homeostasis	7	3	-2.2	1.59E-02	
	zinc ion homeostasis	8	3	-2.2	2.08E-02	
	zinc ion transport	22	4	-2.1	2.47E-02	
