# Supplementary material for: Translation in astrocyte distal processes sets molecular heterogeneity at the gliovascular interface
Source: Cell Discov. 2017 Mar 28;3:17005–. doi: 10.1038/celldisc.2017.5 (PMC5368712; doi:10.1038/celldisc.2017.5)
Supplement: Supplementary Table S3 [file celldisc20175-s5.pdf]

Table S3 The astrocyte endfeet ribosome-bound transcriptome. Selected mRNAs have a mean read ≥ 50 and exon coverage ≥ 80%. The fold change between the ribosome-bound mRNAs in endfeet and whole astrocyte TRAP libraries (Log10 FC) and the associated p value are indicated. Transcripts are ordered following their sequencing rate in the endfeet.

Ensembl ID	Gene name	Gene description	log10 FC	padj	Mean reads "total"	Mean reads "endfeet"	Exon coverage "total"	Exon coverage "endfeet"
ENSMUSG00000061808	Ttr	transthyretin	-1.5	3.8E-07	10767.7	403008.4	94	100
ENSMUSG00000005089	Slc1a2	solute carrier family 1 (glial high affinity glutamate transporter), n	-0.3	6.9E-02	102397.3	197085.7	93	92
ENSMUSG00000037852	Cpe	carboxypeptidase E	-0.6	4.9E-07	42738.0	180164.1	98	97
ENSMUSG00000022425	Enpp2	ectonucleotide pyrophosphatase/phosphodiesterase 2	-1.8	1.9E-17	1629.6	116970.5	93	99
ENSMUSG00000007097	Atp1a2	ATPase, Na+/K+ transporting, alpha 2 polypeptide	-0.7	1.8E-08	19643.3	93438.5	92	94
ENSMUSG00000025203	Scd2	stearoyl-Coenzyme A desaturase 2	-0.3	3.0E-02	28475.0	61091.5	90	90
ENSMUSG00000026678	Rgs5	regulator of G-protein signaling 5	-2.4	2.1E-97	207.0	55962.9	86	89
ENSMUSG00000029309	Sparcl1	SPARC-like 1	-0.5	1.0E-05	15918.2	55489.8	97	93
ENSMUSG00000024140	Epas1	endothelial PAS domain protein 1	-0.7	1.9E-18	9117.4	49546.7	99	99
ENSMUSG00000000184	Ccnd2	cyclin D2	-1.2	2.5E-09	1680.1	31211.3	98	96
ENSMUSG00000022708	Zbtb20	zinc finger and BTB domain containing 20	-0.4	3.8E-04	12298.7	29553.7	87	86
ENSMUSG00000030096	Slc6a6	solute carrier family 6 (neurotransmitter transporter, taurine), men	-1.7	1.0E-57	498.0	28889.9	96	99
ENSMUSG00000009575	Cbx5	chromobox 5	-0.6	4.7E-05	6657.7	28609.6	99	98
ENSMUSG00000031342	Gpm6b	glycoprotein m6b	-0.2	1.5E-01	16587.2	27929.0	86	88
ENSMUSG00000033960	9430020K01	RIKEN cDNA 9430020K01 gene	-1.7	2.2E-65	492.5	27122.1	95	98
ENSMUSG00000058297	Spock2	sparc/osteonectin, cwcv and kazal-like domains proteoglycan 2	-1.4	5.8E-44	934.8	23164.1	89	86
ENSMUSG00000004558	Ndrg2	N-myc downstream regulated gene 2	0.9	9.9E-10	167748.8	21459.7	93	91
ENSMUSG00000028649	Macf1	microtubule-actin crosslinking factor 1	-1.4	3.6E-89	781.4	20809.5	81	86
ENSMUSG00000026436	Elk4	ELK4, member of ETS oncogene family	-1.5	1.7E-22	631.1	20441.5	82	85
ENSMUSG00000024411	Aqp4	aquaporin 4	-0.5	7.0E-04	6366.9	20426.3	99	99
ENSMUSG00000026667	Uhmk1	U2AF homology motif (UHM) kinase 1	-0.4	3.1E-03	7569.7	19071.3	91	89
ENSMUSG00000027663	Zmat3	zinc finger matrin type 3	-0.4	4.8E-02	6581.6	17186.6	90	83
ENSMUSG00000025780	Itih5	inter-alpha (globulin) inhibitor H5	-1.6	2.9E-108	447.7	16880.1	97	98
ENSMUSG00000064370	mt-Cytb	mitochondrially encoded cytochrome b	-2.0	2.6E-82	160.3	16722.1	98	100
ENSMUSG00000002341	Ncan	neurocan	-0.8	2.8E-09	2785.0	16286.6	89	86
ENSMUSG00000064367	mt-Nd5	mitochondrially encoded NADH dehydrogenase 5	-2.2	9.3E-128	85.9	15555.4	91	99
ENSMUSG00000031996	Aplp2	amyloid beta (A4) precursor-like protein 2	-0.9	1.2E-20	1900.8	15519.1	93	94
ENSMUSG00000028211	Trp53inp1	transformation related protein 53 inducible nuclear protein 1	-1.2	1.9E-10	931.2	15481.1	82	81
ENSMUSG00000017707	Serinc3	serine incorporator 3	-1.1	6.8E-65	1149.4	15406.4	93	90
ENSMUSG00000010663	Fads1	fatty acid desaturase 1	-0.2	7.5E-02	8978.6	14949.1	92	94
ENSMUSG00000020849	Ywhae	tyrosine 3-monooxygenase/tryptophan 5-monooxygenase activatio	0.6	5.2E-11	54167.1	14889.2	81	80
ENSMUSG00000064341	mt-Nd1	mitochondrially encoded NADH dehydrogenase 1	-2.1	2.0E-92	115.7	14764.2	100	100
ENSMUSG00000021356	Irf4	interferon regulatory factor 4	-2.2	1.2E-25	84.7	14153.5	85	97
ENSMUSG00000001175	Calm1	calmodulin 1	0.4	2.0E-10	34950.5	14066.3	84	84
ENSMUSG00000008730	Hipk1	homeodomain interacting protein kinase 1	-0.6	4.6E-05	3716.0	13863.8	92	90
ENSMUSG00000019820	Utrn	utrophin	-1.5	6.5E-86	382.9	13714.9	84	95
ENSMUSG00000037071	Scd1	stearoyl-Coenzyme A desaturase 1	-0.4	4.7E-03	5182.4	13428.0	96	95
ENSMUSG00000028645	Slc2a1	solute carrier family 2 (facilitated glucose transporter), member 1	-1.4	2.2E-151	560.1	13303.9	91	91
ENSMUSG00000039218	Srrm2	serine/arginine repetitive matrix 2	-0.3	3.8E-03	7372.6	13290.4	91	90
ENSMUSG00000022108	Itm2b	integral membrane protein 2B	-0.4	6.5E-06	4963.6	13200.8	99	99
ENSMUSG00000021939	Ctsb	cathepsin B	-0.6	1.6E-10	3113.4	12987.5	91	84
ENSMUSG00000062078	Qk	quaking	0.7	6.8E-08	59349.0	12709.6	91	89
ENSMUSG00000024758	Rtn3	reticulon 3	-0.2	5.7E-02	7938.7	12647.7	92	81
ENSMUSG00000020932	Gfap	glial fibrillary acidic protein	0.2	3.6E-01	18668.1	12454.6	87	83
ENSMUSG00000036698	Ago2	argonaute RISC catalytic subunit 2	-0.7	2.3E-05	2341.6	12227.1	87	81
ENSMUSG00000044708	Kcnj10	potassium inwardly-rectifying channel, subfamily J, member 10	-0.3	1.8E-02	5744.5	12097.7	95	96
ENSMUSG00000026234	Ncl	nucleolin	-0.8	2.5E-10	1918.6	12044.5	91	84
ENSMUSG00000094483	Purb	purine rich element binding protein B	-0.1	3.2E-01	8494.7	12037.3	89	88
ENSMUSG00000030516	Tjp1	tight junction protein 1	-0.4	2.0E-06	4500.9	11945.3	91	88
ENSMUSG00000026434	Nucks1	nuclear casein kinase and cyclin-dependent kinase substrate 1	-0.3	1.2E-01	6266.9	11727.1	95	94
ENSMUSG00000026697	Myoc	myocilin	-1.4	1.1E-38	429.3	11713.3	92	98
ENSMUSG00000024991	Eif3a	eukaryotic translation initiation factor 3, subunit A	-0.8	2.0E-09	1839.2	11509.4	89	89
ENSMUSG00000030654	Arl6ip1	ADP-ribosylation factor-like 6 interacting protein 1	-0.5	3.3E-04	3682.1	11425.4	95	95
ENSMUSG00000031447	Lamp1	lysosomal-associated membrane protein 1	-0.5	5.2E-09	3686.1	11167.1	90	91
ENSMUSG00000037503	Fam168b	family with sequence similarity 168, member B	-0.1	NA	7928.9	11133.4	93	82
ENSMUSG00000020368	Canx	calnexin	-0.7	3.1E-17	2098.3	11060.5	89	88
ENSMUSG00000068748	Ptprz1	protein tyrosine phosphatase, receptor type Z, polypeptide 1	-0.7	2.8E-06	2215.0	10927.6	94	93
ENSMUSG00000017291	Taok1	TAO kinase 1	-0.1	4.8E-01	8746.4	10886.8	93	90
ENSMUSG00000024661	Fth1	ferritin heavy chain 1	-0.4	1.8E-05	4180.8	10618.1	94	94
ENSMUSG00000025666	Tmem47	transmembrane protein 47	-0.2	2.2E-01	7128.2	10544.0	99	99
ENSMUSG00000030275	Etnk1	ethanolamine kinase 1	-0.4	6.2E-03	3863.4	10484.8	94	92
ENSMUSG00000052727	Map1b	microtubule-associated protein 1B	-0.1	1.4E-01	8278.9	10320.7	95	95
ENSMUSG00000043131	Mob1a	MOB kinase activator 1A	-0.5	2.3E-03	2828.7	10019.0	88	86
ENSMUSG00000032053	Pou2af1	POU domain, class 2, associating factor 1	-2.1	2.4E-24	63.4	9990.8	83	91
ENSMUSG00000060802	B2m	beta-2 microglobulin	-0.7	1.6E-14	1868.7	9576.2	97	97
ENSMUSG00000055322	Tns1	tensin 1	-0.9	7.3E-27	1172.7	9574.4	87	90
ENSMUSG00000020611	Gna13	guanine nucleotide binding protein, alpha 13	0.4	3.9E-02	21824.5	9565.4	95	87
ENSMUSG00000029467	Atp2a2	ATPase, Ca++ transporting, cardiac muscle, slow twitch 2	-0.3	1.6E-03	4587.5	9536.0	83	84
ENSMUSG00000032186	Tmod2	tropomodulin 2	0.3	4.1E-03	20860.5	9525.1	90	89
ENSMUSG00000059495	Arhgef12	Rho guanine nucleotide exchange factor (GEF) 12	-0.1	2.2E-01	7289.5	9379.5	89	83
ENSMUSG00000058013	Sept11	septin 11	-0.7	4.3E-18	1891.2	9221.4	97	94
ENSMUSG00000044468	Fam46c	family with sequence similarity 46, member C	-1.9	3.8E-25	104.7	9212.6	81	88
ENSMUSG00000038612	Mcl1	myeloid cell leukemia sequence 1	-0.6	6.8E-05	2493.1	9053.0	82	80
ENSMUSG00000017390	Aldoc	aldolase C, fructose-bisphosphate	1.2	4.2E-20	138750.1	8753.7	85	83
ENSMUSG00000041577	Prelp	proline arginine-rich end leucine-rich repeat	-1.0	2.3E-19	850.9	8717.4	99	99
ENSMUSG00000026576	Atp1b1	ATPase, Na+/K+ transporting, beta 1 polypeptide	-0.3	4.8E-02	4739.1	8505.9	84	83
ENSMUSG00000049354	Dcaf7	DDB1 and CUL4 associated factor 7	-0.3	9.1E-02	3625.1	8246.7	86	84
ENSMUSG00000020091	Eif4ebp2	eukaryotic translation initiation factor 4E binding protein 2	-0.9	7.3E-10	947.6	8221.9	93	93
ENSMUSG00000004207	Psap	prosaposin	-0.7	9.4E-08	1685.1	8112.5	87	89
ENSMUSG00000031517	Gpm6a	glycoprotein m6a	0.1	4.5E-01	10686.2	8086.1	92	88
ENSMUSG00000068284	Gm608	predicted gene 608	-0.8	8.7E-12	1301.6	7928.8	92	81
ENSMUSG00000056429	Tgoln1	trans-golgi network protein	-0.7	4.0E-14	1503.0	7894.7	90	93
ENSMUSG00000041852	Tcf20	transcription factor 20	-1.0	3.1E-10	790.8	7886.1	88	80
ENSMUSG00000032399	Rpl4	ribosomal protein L4	-0.6	5.2E-04	1883.8	7862.4	83	81
ENSMUSG00000048960	Prex2	phosphatidylinositol-3,4,5-trisphosphate-dependent Rac exchange	0.2	2.6E-01	11080.0	7783.1	91	87
ENSMUSG00000024294	Mib1	mindbomb homolog 1 (Drosophila)	-0.1	3.2E-01	5633.9	7779.0	88	85
ENSMUSG00000030310	Slc6a1	solute carrier family 6 (neurotransmitter transporter, GABA), men	0.0	8.9E-01	7313.7	7722.0	91	92
ENSMUSG00000057530	Ece1	endothelin converting enzyme 1	-1.3	6.8E-48	425.2	7695.6	83	83
ENSMUSG00000096188	Cmtm4	CKLF-like MARVEL transmembrane domain containing 4	-0.8	2.7E-06	1268.1	7668.8	89	82
ENSMUSG00000002028	Kmt2a	lysine (K)-specific methyltransferase 2A	-0.9	4.4E-15	963.2	7655.4	85	86
ENSMUSG00000026185	Igfbp5	insulin-like growth factor binding protein 5	-0.5	2.7E-05	2232.8	7636.8	97	96
ENSMUSG00000021750	Fam107a	family with sequence similarity 107, member A	1.3	1.7E-37	164879.7	7554.3	85	82

ENSMUSG00000063796	Slc22a8	solute carrier family 22 (organic anion transporter), member 8	-1.5	8.7E-36	257.3	7534.6	89	98
ENSMUSG00000005583	Mef2c	myocyte enhancer factor 2C	-0.3	2.9E-02	3423.1	7531.6	90	83
ENSMUSG00000039100	March6	membrane-associated ring finger (C3HC4) 6	-0.7	8.6E-09	1412.6	7414.6	83	86
ENSMUSG00000021427	Ssr1	signal sequence receptor, alpha	-0.7	1.9E-14	1319.5	7309.1	88	87
ENSMUSG00000030201	Lrp6	low density lipoprotein receptor-related protein 6	-1.2	2.6E-57	469.4	7275.6	82	82
ENSMUSG00000079037	Prnp	prion protein	-0.1	6.6E-01	6202.1	7272.8	91	92
ENSMUSG00000019818	Cd164	CD164 antigen	-0.6	9.3E-06	1970.5	7260.8	97	98
ENSMUSG00000035245	Eogt	EGF domain-specific O-linked N-acetylglucosamine (GlcNAc) tra	-1.1	1.6E-58	578.0	7227.4	87	82
ENSMUSG00000040848	Sft2d2	SFT2 domain containing 2	-0.4	6.6E-03	2603.1	7221.2	95	90
ENSMUSG00000031239	Itm2a	integral membrane protein 2A	-1.8	4.0E-46	97.9	7183.2	88	94
ENSMUSG00000031393	Mecp2	methyl CpG binding protein 2	-0.3	1.1E-02	3850.5	7172.5	89	81
ENSMUSG00000037062	Sh3glb1	SH3-domain GRB2-like B1 (endophilin)	0.0	7.1E-01	7745.1	7146.7	93	88
ENSMUSG00000027763	Mbnl1	muscleblind-like 1 (Drosophila)	-0.2	1.3E-02	4059.3	7138.9	98	95
ENSMUSG00000027630	Tbl1xr1	transducin (beta)-like 1X-linked receptor 1	-0.3	5.7E-02	3528.1	7087.9	91	81
ENSMUSG00000032199	Polr2m	polymerase (RNA) II (DNA directed) polypeptide M	-0.3	3.4E-02	3953.3	7085.5	96	94
ENSMUSG00000023169	Slc38a1	solute carrier family 38, member 1	-0.8	1.4E-13	1213.5	7065.9	83	80
ENSMUSG00000005873	Reep5	receptor accessory protein 5	-0.2	3.2E-02	4191.2	7065.0	96	97
ENSMUSG00000034994	Eef2	eukaryotic translation elongation factor 2	-0.7	1.2E-08	1450.8	7058.3	88	87
ENSMUSG00000055447	Cd47	CD47 antigen (Rh-related antigen, integrin-associated signal trans	-0.6	2.3E-16	1635.6	6907.5	95	93
ENSMUSG00000087370	Tmem170b	transmembrane protein 170B	-0.3	1.7E-01	3652.5	6870.2	97	90
ENSMUSG00000034297	Med13	mediator complex subunit 13	-0.1	3.6E-01	5275.9	6825.5	92	85
ENSMUSG00000042444	Fam63b	family with sequence similarity 63, member B	0.1	6.4E-01	8068.0	6788.5	89	86
ENSMUSG00000036333	Kidins220	kinase D-interacting substrate 220	-0.5	1.0E-14	1908.6	6686.3	92	91
ENSMUSG00000032181	Scg3	secretogranin III	-0.1	8.0E-01	5893.0	6634.9	100	100
ENSMUSG00000067336	Bmpr2	bone morphogenetic protein receptor, type II (serine/threonine kin	-0.8	1.5E-16	1159.6	6595.4	95	91
ENSMUSG00000019132	BC005537	cDNA sequence BC005537	-0.1	6.1E-01	5066.2	6497.3	91	89
ENSMUSG00000017466	Timp2	tissue inhibitor of metalloproteinase 2	-0.6	2.1E-07	1724.7	6463.8	84	82
ENSMUSG00000027162	Lin7c	lin-7 homolog C (C, elegans)	0.1	4.4E-01	8580.9	6443.4	95	93
ENSMUSG00000032050	Rdx	radixin	-0.5	3.1E-14	1961.7	6408.0	84	80
ENSMUSG00000027189	Trim44	tripartite motif-containing 44	-0.1	6.4E-01	5688.5	6398.8	93	87
ENSMUSG00000009569	Mkl2	MKL/myocardin-like 2	-0.5	1.1E-16	1931.2	6374.7	93	86
ENSMUSG00000018846	Pank3	pantothenate kinase 3	-0.3	4.5E-02	3517.1	6373.8	92	88
ENSMUSG00000027852	Nras	neuroblastoma ras oncogene	-0.1	4.8E-01	5075.4	6320.2	91	81
ENSMUSG00000022892	App	amyloid beta (A4) precursor protein	-0.8	4.0E-26	982.5	6270.6	96	96
ENSMUSG00000035847	Ids	iduronate 2-sulfatase	0.0	9.3E-01	6077.3	6249.5	90	93
ENSMUSG00000034088	Hdlbp	high density lipoprotein (HDL) binding protein	-0.5	2.3E-05	2190.9	6236.2	92	91
ENSMUSG00000056602	Fry	furry homolog (Drosophila)	-0.8	3.6E-15	986.7	6226.3	92	84
ENSMUSG00000030235	Slco1c1	solute carrier organic anion transporter family, member 1c1	-1.0	2.3E-28	587.7	6199.5	94	97
ENSMUSG00000028382	Ptbp3	polypyrimidine tract binding protein 3	-0.9	1.3E-13	711.2	6194.9	88	83
ENSMUSG00000025016	Tm9sf3	transmembrane 9 superfamily member 3	-0.7	3.3E-20	1315.5	6180.9	92	92
ENSMUSG00000052387	Trpm3	transient receptor potential cation channel, subfamily M, member	-0.9	6.6E-07	805.1	6110.3	84	89
ENSMUSG00000055065	Ddx17	DEAD (Asp-Glu-Ala-Asp) box polypeptide 17	-0.1	3.4E-01	4717.6	6100.4	81	80
ENSMUSG00000058655	Eif4b	eukaryotic translation initiation factor 4B	-0.8	4.9E-07	971.8	6036.9	85	86
ENSMUSG00000050953	Gja1	gap junction protein, alpha 1	0.0	9.9E-01	6004.3	6027.7	98	95
ENSMUSG00000032324	Tspan3	tetraspanin 3	-0.2	7.4E-02	4109.3	6005.5	92	87
ENSMUSG00000020580	Rock2	Rho-associated coiled-coil containing protein kinase 2	-0.1	1.3E-01	4315.1	6003.7	88	89
ENSMUSG00000030307	Slc6a11	solute carrier family 6 (neurotransmitter transporter, GABA), men	-0.1	4.5E-01	4501.3	5999.1	91	89
ENSMUSG00000058690	Ccser2	coiled-coil serine rich 2	-0.3	6.5E-06	3292.6	5995.0	83	83
ENSMUSG00000001089	Luzp1	leucine zipper protein 1	-1.0	2.2E-18	613.0	5979.3	86	85
ENSMUSG00000027002	Nckap1	NCK-associated protein 1	0.2	1.3E-03	10271.8	5964.9	85	81
ENSMUSG00000041028	Ghitm	growth hormone inducible transmembrane protein	0.4	4.4E-12	14552.9	5943.9	94	88
ENSMUSG00000030306	Tmtc1	transmembrane and tetratricopeptide repeat containing 1	-1.3	7.4E-40	278.6	5923.2	81	88
ENSMUSG00000021488	Nsd1	nuclear receptor-binding SET-domain protein 1	-0.4	8.5E-09	2543.4	5770.9	89	86
ENSMUSG00000022048	Dpysl2	dihydropyrimidinase-like 2	0.1	2.8E-01	7889.7	5698.3	93	88
ENSMUSG00000027177	Hipk3	homeodomain interacting protein kinase 3	-0.7	6.3E-13	1013.8	5624.0	86	81
ENSMUSG00000066150	Slc31a1	solute carrier family 31, member 1	-0.8	6.5E-11	790.4	5603.2	84	87
ENSMUSG00000041559	Fmod	fibromodulin	-1.2	1.5E-15	361.4	5601.9	87	90
ENSMUSG00000035805	Mlc1	megalencephalic leukoencephalopathy with subcortical cysts 1 ho	-0.3	9.9E-02	2921.6	5582.5	88	80
ENSMUSG00000001280	Sp1	trans-acting transcription factor 1	-0.6	3.8E-10	1491.2	5560.8	89	80
ENSMUSG00000038370	Pcp4l1	Purkinje cell protein 4-like 1	-0.4	1.6E-05	2091.2	5541.1	99	98
ENSMUSG00000037815	Ctnna1	catenin (cadherin associated protein), alpha 1	-0.1	2.7E-01	4252.1	5534.2	95	90
ENSMUSG00000033965	Slc16a2	solute carrier family 16 (monocarboxylic acid transporters), memb	-1.4	8.1E-75	211.4	5504.6	89	92
ENSMUSG00000021696	Elovl7	ELOVL family member 7, elongation of long chain fatty acids (ye	-1.7	5.2E-31	111.1	5486.7	83	91
ENSMUSG00000032883	Acsl3	acyl-CoA synthetase long-chain family member 3	0.2	4.2E-01	7960.4	5410.4	82	83
ENSMUSG00000021796	Bmpr1a	bone morphogenetic protein receptor, type 1A	-0.3	6.4E-04	2513.4	5409.2	86	81
ENSMUSG00000028053	Ash1l	ash1 (absent, small, or homeotic)-like (Drosophila)	-0.1	3.2E-01	3828.9	5334.3	93	80
ENSMUSG00000062014	Gmfb	glia maturation factor, beta	0.3	2.0E-01	9588.8	5328.8	90	91
ENSMUSG00000032393	Dpp8	dipeptidylpeptidase 8	-0.1	7.4E-01	4682.1	5294.1	94	93
ENSMUSG00000022136	Dnajc3	DnaJ (Hsp40) homolog, subfamily C, member 3	-0.8	6.2E-15	756.1	5282.7	93	96
ENSMUSG00000048578	Mlec	malectin	-0.8	5.3E-15	744.8	5243.2	93	90
ENSMUSG00000030104	Edem1	ER degradation enhancer, mannosidase alpha-like 1	-1.4	1.3E-14	199.4	5241.9	83	81
ENSMUSG00000020088	Sar1a	SAR1 gene homolog A (S, cerevisiae)	-0.1	6.3E-01	4610.0	5212.9	96	94
ENSMUSG00000026219	Trip12	thyroid hormone receptor interactor 12	-0.3	2.8E-03	2821.2	5188.7	89	81
ENSMUSG00000043252	Tmem64	transmembrane protein 64	-0.8	5.1E-10	898.7	5174.4	87	86
ENSMUSG00000032562	Gnai2	guanine nucleotide binding protein (G protein), alpha inhibiting 2	-0.1	4.1E-01	3876.5	5137.6	95	92
ENSMUSG00000038095	Sbno1	sno, strawberry notch homolog 1 (Drosophila)	-0.5	7.3E-08	1729.5	5110.8	95	88
ENSMUSG00000021171	Esyt2	extended synaptotagmin-like protein 2	-1.3	3.7E-82	229.4	5076.6	82	84
ENSMUSG00000051510	Mafg	v-maf musculoaponeurotic fibrosarcoma oncogene family, protein	-1.1	4.2E-09	365.0	5052.8	84	83
ENSMUSG00000017009	Sdc4	syndecan 4	0.0	9.4E-01	5244.5	5023.7	90	92
ENSMUSG00000047414	Flrt2	fibronectin leucine rich transmembrane protein 2	-1.1	4.5E-30	430.4	4944.6	88	88
ENSMUSG00000052397	Ezr	ezrin	-0.1	6.1E-01	3884.8	4942.1	86	85
ENSMUSG00000020029	Nudt4	nudix (nucleoside diphosphate linked moiety X)-type motif 4	-0.3	1.9E-03	2468.4	4919.7	84	83
ENSMUSG00000025241	Fyco1	FYVE and coiled-coil domain containing 1	-0.9	1.3E-30	593.3	4900.7	80	84
ENSMUSG00000029004	Kmt2e	lysine (K)-specific methyltransferase 2E	-0.1	1.1E-01	3480.1	4857.6	88	86
ENSMUSG00000030218	Mgp	matrix Gla protein	-1.7	6.5E-53	89.7	4856.2	94	97
ENSMUSG00000030894	Tpp1	tripeptidyl peptidase I	-0.5	1.3E-06	1490.6	4848.4	96	92
ENSMUSG00000003970	Rpl8	ribosomal protein L8	-0.3	1.0E-01	2594.5	4821.2	93	91
ENSMUSG00000024654	Asrgl1	asparaginase like 1	0.8	4.7E-10	32853.6	4793.5	94	90
ENSMUSG00000066568	Lsm14a	LSM14 homolog A (SCD6, S, cerevisiae)	-0.2	4.8E-02	3271.3	4772.7	89	88
ENSMUSG00000013662	Atad1	ATPase family, AAA domain containing 1	-0.5	3.1E-04	1521.7	4759.6	96	90
ENSMUSG00000032244	Fem1b	feminization 1 homolog b (C, elegans)	-0.1	5.0E-01	3667.8	4721.9	93	81
ENSMUSG00000003131	Pafah1b2	platelet-activating factor acetylhydrolase, isoform 1b, subunit 2	0.2	2.8E-01	6937.1	4709.0	97	90
ENSMUSG00000053279	Aldh1a1	aldehyde dehydrogenase family 1, subfamily A1	0.7	7.8E-08	22006.8	4668.5	86	81
ENSMUSG00000038615	Nfe2l1	nuclear factor, erythroid derived 2,-like 1	-0.6	2.0E-11	1096.1	4663.8	81	85
ENSMUSG00000025931	Paqr8	progestin and adipoQ receptor family member VIII	0.1	6.7E-01	5568.4	4647.4	93	95

ENSMUSG00000030002	Dusp11	dual specificity phosphatase 11 (RNA/RNP complex 1-interacting	-0.4	6.3E-06	1660.6	4644.1	85	80
ENSMUSG00000026112	Coa5	cytochrome C oxidase assembly factor 5	-0.1	7.5E-01	3913.8	4592.0	96	89
ENSMUSG00000045092	S1pr1	sphingosine-1-phosphate receptor 1	0.4	1.6E-08	11517.0	4576.4	93	95
ENSMUSG00000027808	Serp1	stress-associated endoplasmic reticulum protein 1	0.1	6.0E-01	6199.7	4538.9	92	83
ENSMUSG00000025809	Itgb1	integrin beta 1 (fibronectin receptor beta)	-1.1	1.6E-25	321.3	4474.7	81	86
ENSMUSG00000028517	Ppap2b	phosphatidic acid phosphatase type 2B	0.3	1.3E-01	8290.3	4453.2	94	94
ENSMUSG00000065954	Tacc1	transforming, acidic coiled-coil containing protein 1	-0.5	1.9E-06	1427.0	4432.9	88	89
ENSMUSG00000074305	C230081A13	RIKEN cDNA C230081A13 gene	-1.0	4.3E-34	424.1	4430.8	85	80
ENSMUSG00000068747	Sort1	sortilin 1	-0.6	7.2E-12	1177.3	4425.5	81	81
ENSMUSG00000029571	Tmem106b	transmembrane protein 106B	-0.2	1.6E-02	2592.8	4362.9	83	84
ENSMUSG00000026424	Gpr37l1	G protein-coupled receptor 37-like 1	0.0	8.2E-01	4791.5	4347.7	93	88
ENSMUSG00000034485	Uaca	uveal autoantigen with coiled-coil domains and ankyrin repeats	-1.6	1.7E-68	104.6	4327.7	86	92
ENSMUSG00000031592	Pcm1	pericentriolar material 1	-0.3	3.1E-02	2139.5	4313.4	90	88
ENSMUSG00000057522	Spop	speckle-type POZ protein	-0.7	1.6E-12	807.8	4280.0	90	89
ENSMUSG00000037487	Ubr5	ubiquitin protein ligase E3 component n-recognin 5	-0.3	4.1E-04	1963.2	4273.7	88	84
ENSMUSG00000027007	Ssfa2	sperm specific antigen 2	-0.5	8.5E-08	1235.6	4237.7	80	80
ENSMUSG00000029840	Mtpn	myotrophin	0.6	2.4E-07	16347.3	4233.5	91	87
ENSMUSG00000005683	Cs	citrate synthase	0.5	5.7E-08	14756.3	4209.5	94	87
ENSMUSG00000055204	Ankrd17	ankyrin repeat domain 17	-0.2	5.7E-02	2417.7	4148.1	83	80
ENSMUSG00000022016	Akap11	A kinase (PRKA) anchor protein 11	-0.5	4.0E-06	1285.9	4117.4	85	83
ENSMUSG00000020340	Cyfip2	cytoplasmic FMR1 interacting protein 2	-0.1	5.7E-01	3597.6	4117.0	83	80
ENSMUSG00000004364	Cul3	cullin 3	0.2	1.8E-02	7244.3	4112.9	88	82
ENSMUSG00000019873	Reep3	receptor accessory protein 3	-0.7	9.7E-08	881.4	4109.9	80	81
ENSMUSG00000031924	Cyb5b	cytochrome b5 type B	0.2	9.9E-02	7002.0	4088.5	96	91
ENSMUSG00000031532	Tmem66	transmembrane protein 66	-0.5	4.0E-06	1204.6	4077.5	86	87
ENSMUSG00000000346	Dazap2	DAZ associated protein 2	0.7	3.5E-15	18399.1	4076.0	86	86
ENSMUSG00000005534	Insr	insulin receptor	-1.0	2.1E-14	389.8	4052.4	82	83
ENSMUSG00000022462	Slc38a2	solute carrier family 38, member 2	-0.7	1.9E-22	737.9	4040.0	93	91
ENSMUSG00000041417	Pik3r1	phosphatidylinositol 3-kinase, regulatory subunit, polypeptide 1 (p	0.2	8.6E-02	6524.1	4037.3	89	81
ENSMUSG00000035133	Arhgap5	Rho GTPase activating protein 5	0.5	2.5E-06	13159.9	4027.6	95	95
ENSMUSG00000073725	Lmbrd1	LMBR1 domain containing 1	-0.6	5.4E-06	1001.3	4012.3	93	90
ENSMUSG00000066324	Impad1	inositol monophosphatase domain containing 1	-0.4	5.0E-03	1613.4	4006.1	88	85
ENSMUSG00000038530	Rgs4	regulator of G-protein signaling 4	-0.3	2.8E-03	1890.3	3987.3	91	90
ENSMUSG00000032340	Neo1	neogenin	-0.7	5.5E-14	822.3	3957.4	82	82
ENSMUSG00000032549	Rab6b	RAB6B, member RAS oncogene family	0.4	9.1E-05	9514.9	3944.0	95	86
ENSMUSG00000024098	Twsg1	twisted gastrulation homolog 1 (Drosophila)	-0.8	3.2E-13	552.9	3920.4	92	90
ENSMUSG00000019929	Dcn	decorin	-1.5	3.6E-44	130.2	3894.4	88	90
ENSMUSG00000021390	Ogn	osteoglycin	-1.2	2.0E-26	242.4	3843.7	99	100
ENSMUSG00000034707	Gns	glucosamine (N-acetyl)-6-sulfatase	-1.0	3.0E-42	423.5	3812.9	90	91
ENSMUSG00000038481	Cdk19	cyclin-dependent kinase 19	-0.2	1.0E-01	2162.2	3783.8	91	84
ENSMUSG00000022122	Ednrb	endothelin receptor type B	-0.2	3.3E-01	2522.2	3775.8	95	95
ENSMUSG00000033629	Ptplad1	protein tyrosine phosphatase-like A domain containing 1	-0.1	5.2E-01	2718.1	3725.3	91	91
ENSMUSG00000021477	Ctsl	cathepsin L	-0.5	1.2E-05	1211.4	3721.5	89	96
ENSMUSG00000025130	P4hb	prolyl 4-hydroxylase, beta polypeptide	-0.9	7.1E-16	499.5	3716.4	84	85
ENSMUSG00000039952	Dag1	dystroglycan 1	-0.7	2.7E-19	724.3	3714.6	88	85
ENSMUSG00000089774	Slc5a3	solute carrier family 5 (inositol transporters), member 3	-1.0	6.3E-14	336.1	3686.4	80	86
ENSMUSG00000024302	Dtna	dystrobrevin alpha	0.5	5.5E-04	12433.5	3672.6	89	84
ENSMUSG00000029238	Clock	circadian locomotor output cycles kaput	-0.3	8.2E-03	1714.9	3663.0	91	85
ENSMUSG00000025982	Sf3b1	splicing factor 3b, subunit 1	-0.2	1.4E-01	2234.6	3642.9	93	89
ENSMUSG00000024782	Ak3	adenylate kinase 3	0.5	7.9E-06	12425.5	3638.7	91	94
ENSMUSG00000023150	Ivns1abp	influenza virus NS1A binding protein	0.0	7.9E-01	3275.2	3616.9	89	83
ENSMUSG00000037712	Fermt2	fermitin family homolog 2 (Drosophila)	0.4	2.3E-07	10181.4	3600.2	84	83
ENSMUSG00000074994	Qser1	glutamine and serine rich 1	-0.6	1.8E-08	904.0	3598.8	91	82
ENSMUSG00000021978	Extl3	exostoses (multiple)-like 3	-0.5	4.6E-29	1086.4	3581.0	88	87
ENSMUSG00000021366	Hivep1	human immunodeficiency virus type I enhancer binding protein 1	-0.5	2.0E-07	1089.7	3567.7	84	83
ENSMUSG00000031751	Amfr	autocrine motility factor receptor	-0.5	2.9E-13	1058.7	3531.5	80	82
ENSMUSG00000034160	Ogt	O-linked N-acetylglucosamine (GlcNAc) transferase (UDP-N-ace	-0.5	7.9E-04	1172.2	3526.8	82	87
ENSMUSG00000040010	Slc7a5	solute carrier family 7 (cationic amino acid transporter, y+ system	-1.1	1.1E-21	266.2	3505.4	83	80
ENSMUSG00000017776	Crk	v-crk sarcoma virus CT10 oncogene homolog (avian)	0.2	5.1E-02	5431.6	3505.4	85	85
ENSMUSG00000019966	Kitl	kit ligand	-1.2	5.6E-21	213.5	3503.9	85	86
ENSMUSG00000020423	Btg2	B cell translocation gene 2, anti-proliferative	-1.2	1.9E-10	202.3	3502.8	94	91
ENSMUSG00000024900	Cpt1a	carnitine palmitoyltransferase 1a, liver	-0.2	1.4E-01	2044.8	3470.1	84	80
ENSMUSG00000038708	Golga4	golgi autoantigen, golgin subfamily a, 4	-0.6	2.1E-08	770.0	3447.5	91	88
ENSMUSG00000020591	Ntsr2	neurotensin receptor 2	0.1	3.2E-01	4863.2	3446.9	89	81
ENSMUSG00000027962	Vcam1	vascular cell adhesion molecule 1	-0.5	3.1E-04	1210.7	3445.6	89	90
ENSMUSG00000021027	Ralgapa1	Ral GTPase activating protein, alpha subunit 1	-0.5	9.0E-05	985.3	3443.3	88	88
ENSMUSG00000030536	Iqgap1	IQ motif containing GTPase activating protein 1	-1.3	7.8E-93	164.3	3442.2	84	83
ENSMUSG00000048388	Fam171b	family with sequence similarity 171, member B	0.3	1.3E-02	7150.0	3407.0	89	88
ENSMUSG00000021709	Erbb2ip	Erbb2 interacting protein	-0.1	6.2E-01	2943.6	3392.3	89	85
ENSMUSG00000060657	Marf1	meiosis arrest female 1	-0.4	2.0E-05	1273.1	3391.3	92	80
ENSMUSG00000026782	Abi2	abl-interactor 2	0.1	9.1E-02	4699.8	3363.2	93	88
ENSMUSG00000074746	Pdzd8	PDZ domain containing 8	-0.7	2.2E-15	629.0	3353.7	82	82
ENSMUSG00000045767	B230219D22	RIKEN cDNA B230219D22 gene	-0.1	3.7E-01	2531.7	3351.5	93	87
ENSMUSG00000025810	Nrp1	neuropilin 1	-1.3	1.9E-24	175.1	3314.1	85	86
ENSMUSG00000021669	Col4a3bp	collagen, type IV, alpha 3 (Goodpasture antigen) binding protein	-0.5	9.2E-20	1023.4	3291.8	89	81
ENSMUSG00000068566	Myadm	myeloid-associated differentiation marker	-1.1	8.4E-45	239.3	3274.6	86	88
ENSMUSG00000039361	Picalm	phosphatidylinositol binding clathrin assembly protein	-0.1	5.2E-01	2805.4	3271.1	90	87
ENSMUSG00000034024	Cct2	chaperonin containing Tcp1, subunit 2 (beta)	-0.1	NA	2345.3	3262.1	99	95
ENSMUSG00000026421	Csrp1	cysteine and glycine-rich protein 1	0.8	5.8E-14	21212.6	3253.9	87	85
ENSMUSG00000050912	Tmem123	transmembrane protein 123	-1.2	4.6E-36	218.5	3249.1	83	89
ENSMUSG00000038014	Fam120a	family with sequence similarity 120, member A	0.0	1.0E+00	3201.8	3210.9	86	80
ENSMUSG00000031980	Agt	angiotensinogen (serpin peptidase inhibitor, clade A, member 8)	-0.6	5.0E-04	771.1	3194.0	95	97
ENSMUSG00000020326	Ccng1	cyclin G1	0.2	3.2E-01	4880.7	3190.6	96	85
ENSMUSG00000067818	Myl9	myosin, light polypeptide 9, regulatory	-1.0	1.8E-17	319.2	3190.4	96	94
ENSMUSG00000039959	Hip1	huntingtin interacting protein 1	-0.3	1.4E-06	1644.3	3154.2	84	83
ENSMUSG00000020572	Nampt	nicotinamide phosphoribosyltransferase	-0.4	2.6E-15	1243.9	3152.2	83	82
ENSMUSG00000027803	Wwtr1	WW domain containing transcription regulator 1	0.0	9.0E-01	3246.9	3147.5	92	88
ENSMUSG00000020850	Prpf8	pre-mRNA processing factor 8	-0.2	2.1E-02	2056.9	3101.1	92	83
ENSMUSG00000004980	Hnrnpa2b1	heterogeneous nuclear ribonucleoprotein A2/B1	0.3	9.4E-04	5802.8	3093.4	84	82
ENSMUSG00000031538	Plat	plasminogen activator, tissue	-1.4	5.1E-22	106.2	3072.2	85	89
ENSMUSG00000000078	Klf6	Kruppel-like factor 6	-0.3	1.9E-02	1448.0	3069.7	91	85
ENSMUSG00000031703	Itfg1	integrin alpha FG-GAP repeat containing 1	-0.4	1.5E-04	1219.1	3060.0	90	88
ENSMUSG00000031785	Gpr56	G protein-coupled receptor 56	-0.5	3.8E-07	1042.5	3013.2	87	88
ENSMUSG00000017615	Tnfaip1	tumor necrosis factor, alpha-induced protein 1 (endothelial)	-0.1	4.5E-01	2236.5	3000.0	90	85
ENSMUSG00000096054	Syne1	spectrin repeat containing, nuclear envelope 1	-0.8	2.4E-12	468.6	2987.8	89	83

ENSMUSG00000019699	Akt3	thymoma viral proto-oncogene 3	0.0	9.5E-01	3039.4	2982.7	92	86
ENSMUSG00000020671	Rab10	RAB10, member RAS oncogene family	0.6	7.6E-08	11448.9	2978.9	87	82
ENSMUSG00000015749	Anp32e	acidic (leucine-rich) nuclear phosphoprotein 32 family, member E	0.2	3.5E-02	4909.3	2978.6	82	86
ENSMUSG00000039967	Zfp292	zinc finger protein 292	-0.5	7.6E-07	1041.6	2973.5	89	80
ENSMUSG00000019978	Epb4.1l2	erythrocyte protein band 4,1-like 2	0.2	1.9E-02	4449.2	2968.6	88	89
ENSMUSG00000003316	Glg1	golgi apparatus protein 1	-0.7	5.0E-14	604.2	2955.7	86	84
ENSMUSG00000022141	Nipbl	Nipped-B homolog (Drosophila)	-0.4	1.3E-04	1289.2	2945.9	89	84
ENSMUSG00000020954	Strn3	striatin, calmodulin binding protein 3	0.1	1.6E-01	4004.9	2942.4	86	82
ENSMUSG00000021484	Lman2	lectin, mannose-binding 2	-0.6	2.2E-26	730.3	2939.8	90	84
ENSMUSG00000021728	Emb	embigin	-1.3	3.8E-09	136.4	2938.3	85	92
ENSMUSG00000022200	Golph3	golgi phosphoprotein 3	0.4	3.3E-04	7296.1	2935.3	89	85
ENSMUSG00000027712	Anxa5	annexin A5	0.3	3.8E-02	5382.8	2932.7	89	88
ENSMUSG00000031246	Sh3bgrl	SH3-binding domain glutamic acid-rich protein like	0.1	1.6E-01	4075.9	2925.2	83	87
ENSMUSG00000056211	R3hdm1	R3H domain containing 1	-0.1	5.6E-01	2343.0	2915.3	93	94
ENSMUSG00000027562	Car2	carbonic anhydrase 2	1.0	4.5E-09	27630.9	2905.8	87	88
ENSMUSG00000003226	Ranbp2	RAN binding protein 2	-0.4	7.4E-05	1060.3	2882.9	88	87
ENSMUSG00000020598	Nrcam	neuron-glia-CAM-related cell adhesion molecule	-0.3	7.8E-03	1416.6	2876.4	85	82
ENSMUSG00000006373	Pgrmc1	progesterone receptor membrane component 1	-0.7	9.2E-15	522.5	2870.2	87	91
ENSMUSG00000024603	Dctn4	dynactin 4	0.4	1.4E-03	6539.6	2847.1	96	83
ENSMUSG00000024290	Rock1	Rho-associated coiled-coil containing protein kinase 1	-0.4	1.9E-05	1010.2	2799.2	88	82
ENSMUSG00000015501	Hivep2	human immunodeficiency virus type I enhancer binding protein 2	-0.3	1.9E-03	1307.5	2798.6	85	81
ENSMUSG00000022521	Crebbp	CREB binding protein	-0.4	4.3E-03	1151.8	2781.0	89	81
ENSMUSG00000025935	Tram1	translocating chain-associating membrane protein 1	-0.8	3.7E-17	423.0	2749.8	93	97
ENSMUSG00000024533	Spire1	spire homolog 1 (Drosophila)	0.6	1.6E-07	10374.2	2730.4	86	84
ENSMUSG00000000001	Gnai3	guanine nucleotide binding protein (G protein), alpha inhibiting 3	0.1	4.4E-01	3397.2	2729.6	94	89
ENSMUSG00000022314	Rad21	RAD21 homolog (S, pombe)	0.1	1.3E-01	3505.3	2705.6	89	88
ENSMUSG00000031701	Dnaja2	DnaJ (Hsp40) homolog, subfamily A, member 2	0.3	1.1E-01	5482.8	2701.6	98	93
ENSMUSG00000093930	Hmgcs1	3-hydroxy-3-methylglutaryl-Coenzyme A synthase 1	0.5	4.4E-06	8801.1	2668.6	88	81
ENSMUSG00000037608	Bclaf1	BCL2-associated transcription factor 1	0.3	1.2E-02	5373.5	2645.9	89	84
ENSMUSG00000007476	Lrrc8a	leucine rich repeat containing 8A	-0.4	4.3E-06	1076.1	2611.2	87	80
ENSMUSG00000035770	Dync1li2	dynein, cytoplasmic 1 light intermediate chain 2	0.1	4.0E-01	3229.0	2603.3	86	83
ENSMUSG00000037236	Matr3	matrin 3	-0.1	6.9E-01	2280.8	2590.1	88	80
ENSMUSG00000060510	Zfp266	zinc finger protein 266	-0.1	NA	1931.5	2587.6	90	86
ENSMUSG00000001472	Tcf25	transcription factor 25 (basic helix-loop-helix)	0.3	1.2E-02	4924.8	2568.3	96	82
ENSMUSG00000054408	Spcs3	signal peptidase complex subunit 3 homolog (S, cerevisiae)	-0.6	4.0E-15	655.1	2563.8	97	95
ENSMUSG00000023960	Enpp5	ectonucleotide pyrophosphatase/phosphodiesterase 5	-0.4	1.2E-02	1062.4	2561.9	90	91
ENSMUSG00000030245	Golt1b	golgi transport 1 homolog B (S, cerevisiae)	-0.8	2.3E-04	352.9	2551.0	95	80
ENSMUSG00000058587	Tmod3	tropomodulin 3	-0.3	1.8E-03	1239.7	2530.8	94	91
ENSMUSG00000029461	Fam168a	family with sequence similarity 168, member A	0.4	8.0E-04	6118.9	2525.7	86	81
ENSMUSG00000054008	Ndst1	N-deacetylase/N-sulfotransferase (heparan glucosaminyl) 1	-0.7	3.2E-07	477.1	2498.4	92	85
ENSMUSG00000064210	Ano6	anoctamin 6	-1.0	1.9E-31	229.7	2479.4	82	84
ENSMUSG00000030016	Zfml	zinc finger, matrin-like	0.3	3.6E-02	4430.8	2476.6	90	80
ENSMUSG00000022261	Sdc2	syndecan 2	-0.3	7.4E-04	1159.2	2447.3	92	89
ENSMUSG00000020300	Cpeb4	cytoplasmic polyadenylation element binding protein 4	0.3	2.5E-02	4433.8	2439.5	88	80
ENSMUSG00000023845	Lnpep	leucyl/cystinyl aminopeptidase	-0.7	3.8E-07	520.5	2427.9	95	87
ENSMUSG00000029178	Klf3	Kruppel-like factor 3 (basic)	-0.2	2.2E-01	1653.3	2412.5	82	84
ENSMUSG00000024350	Dnajc18	DnaJ (Hsp40) homolog, subfamily C, member 18	-0.1	4.2E-01	1945.8	2408.4	92	90
ENSMUSG00000037119	D15Ertd621e	DNA segment, Chr 15, ERATO Doi 621, expressed	0.1	3.7E-01	3051.2	2408.4	89	80
ENSMUSG00000022139	Mbnl2	muscleblind-like 2	0.3	2.7E-04	4856.8	2396.0	93	93
ENSMUSG00000024712	Rfk	riboflavin kinase	0.2	3.3E-02	3822.1	2385.3	86	87
ENSMUSG00000024665	Fads2	fatty acid desaturase 2	-0.4	1.5E-03	948.4	2383.5	97	100
ENSMUSG00000026393	Nek7	NIMA (never in mitosis gene a)-related expressed kinase 7	0.1	6.9E-01	2857.4	2375.1	93	89
ENSMUSG00000028277	Ube2j1	ubiquitin-conjugating enzyme E2J 1	0.0	9.1E-01	2552.0	2372.5	94	83
ENSMUSG00000031639	Tlr3	toll-like receptor 3	-0.3	8.2E-02	1230.0	2372.2	90	87
ENSMUSG00000031983	2310022B05	RIKEN cDNA 2310022B05 gene	0.7	6.3E-10	12555.7	2370.2	87	81
ENSMUSG00000024083	Pja2	praja 2, RING-H2 motif containing	0.1	5.3E-01	2646.1	2355.8	89	81
ENSMUSG00000024423	Impact	imprinted and ancient	0.3	1.6E-01	4438.1	2353.2	95	88
ENSMUSG00000031451	Gas6	growth arrest specific 6	-0.9	7.1E-16	280.3	2344.2	87	89
ENSMUSG00000053094	Tmem248	transmembrane protein 248	-0.9	4.5E-12	320.4	2332.2	83	80
ENSMUSG00000040731	Eif4h	eukaryotic translation initiation factor 4H	0.3	7.4E-05	4166.8	2330.2	89	80
ENSMUSG00000022505	Emp2	epithelial membrane protein 2	-0.6	3.2E-12	533.1	2329.2	87	84
ENSMUSG00000040021	Lats1	large tumor suppressor	0.0	7.5E-01	2602.4	2325.6	86	83
ENSMUSG00000041935	AW549877	expressed sequence AW549877	0.3	1.5E-01	4297.9	2321.8	96	84
ENSMUSG00000030605	Mfge8	milk fat globule-EGF factor 8 protein	0.0	9.5E-01	2261.7	2319.9	86	81
ENSMUSG00000054459	Vsnl1	visinin-like 1	0.6	7.3E-08	8745.3	2309.2	97	82
ENSMUSG00000038975	Rabggtb	RAB geranylgeranyl transferase, b subunit	0.1	7.1E-01	2787.7	2304.8	83	81
ENSMUSG00000060450	Rnf14	ring finger protein 14	0.4	1.7E-03	5589.4	2286.4	84	81
ENSMUSG00000040289	Hey1	hairy/enhancer-of-split related with YRPW motif 1	0.5	4.6E-03	7644.0	2276.3	90	80
ENSMUSG00000025132	Arhgdia	Rho GDP dissociation inhibitor (GDI) alpha	0.3	2.0E-03	5103.8	2272.8	86	80
ENSMUSG00000021843	Ktn1	kinectin 1	-0.7	1.1E-09	485.3	2266.0	87	85
ENSMUSG00000024650	Slc22a6	solute carrier family 22 (organic anion transporter), member 6	-1.1	2.4E-16	155.1	2257.5	87	85
ENSMUSG00000022228	Zscan26	zinc finger and SCAN domain containing 26	-0.2	1.1E-01	1313.5	2227.7	87	80
ENSMUSG00000026353	Ubxn4	UBX domain protein 4	-0.1	6.5E-01	1883.1	2221.1	86	83
ENSMUSG00000069874	Irgm2	immunity-related GTPase family M member 2	-1.0	1.4E-17	206.8	2215.9	85	84
ENSMUSG00000030934	Oat	ornithine aminotransferase	0.6	4.7E-04	8101.6	2194.5	88	85
ENSMUSG00000033998	Kcnk1	potassium channel, subfamily K, member 1	0.2	1.9E-01	3532.3	2194.4	85	85
ENSMUSG00000026615	Eprs	glutamyl-prolyl-tRNA synthetase	0.2	1.6E-01	3199.6	2178.7	93	85
ENSMUSG00000022564	Grina	glutamate receptor, ionotropic, N-methyl D-aspartate-associated p	0.1	5.0E-01	2895.8	2144.8	88	92
ENSMUSG00000030660	Pik3c2a	phosphatidylinositol 3-kinase, C2 domain containing, alpha polyp	-0.3	3.1E-05	1054.7	2134.5	83	82
ENSMUSG00000033981	Gria2	glutamate receptor, ionotropic, AMPA2 (alpha 2)	0.0	9.8E-01	2110.8	2133.0	87	85
ENSMUSG00000028698	Pik3r3	phosphatidylinositol 3 kinase, regulatory subunit, polypeptide 3 (p	-0.4	2.3E-13	794.9	2124.2	94	84
ENSMUSG00000059336	Slc14a1	solute carrier family 14 (urea transporter), member 1	-0.3	1.1E-01	1161.6	2122.5	87	85
ENSMUSG00000031902	Nfatc3	nuclear factor of activated T cells, cytoplasmic, calcineurin depen	-0.5	3.2E-04	736.4	2102.8	89	80
ENSMUSG00000033208	S100b	S100 protein, beta polypeptide, neural	1.1	2.7E-12	25217.0	2100.7	94	96
ENSMUSG00000024587	Nars	asparaginyl-tRNA synthetase	0.2	9.4E-02	3174.0	2100.3	97	91
ENSMUSG00000024583	Txnl1	thioredoxin-like 1	0.2	3.0E-01	3377.7	2098.0	92	86
ENSMUSG00000018377	Vezf1	vascular endothelial zinc finger 1	0.2	1.9E-01	3598.6	2097.0	87	83
ENSMUSG00000035237	Lcat	lecithin cholesterol acyltransferase	-0.3	7.4E-03	1094.5	2075.4	98	98
ENSMUSG00000005533	Igf1r	insulin-like growth factor I receptor	-1.3	1.2E-28	99.9	2069.1	84	81
ENSMUSG00000026374	Tsn	translin	0.0	8.0E-01	2292.4	2065.8	95	91
ENSMUSG00000039542	Ncam1	neural cell adhesion molecule 1	-0.5	6.3E-05	666.7	2049.1	88	87
ENSMUSG00000025492	Ifitm3	interferon induced transmembrane protein 3	-0.9	1.1E-16	248.9	2021.7	88	89
ENSMUSG00000052681	Rap1b	RAS related protein 1b	0.0	8.0E-01	1862.4	2017.6	92	86
ENSMUSG00000036503	Rnf13	ring finger protein 13	-0.2	1.8E-01	1314.9	2013.6	94	98
ENSMUSG00000023915	Tnfrsf21	tumor necrosis factor receptor superfamily, member 21	-0.8	3.2E-11	344.2	2007.1	82	82

ENSMUSG00000037235	Mxd4	Max dimerization protein 4	0.2	1.6E-03	3467.8	1999.9	87	81
ENSMUSG00000025373	Rnf41	ring finger protein 41	-0.4	7.1E-02	848.7	1994.3	91	80
ENSMUSG00000041702	Btbd7	BTB (POZ) domain containing 7	-0.7	1.2E-06	404.3	1991.4	87	82
ENSMUSG00000030105	Arl8b	ADP-ribosylation factor-like 8B	0.5	1.9E-04	5646.1	1980.3	93	89
ENSMUSG00000001833	Sept7	septin 7	0.5	6.7E-15	6991.4	1976.5	90	88
ENSMUSG00000031839	Hsbp1	heat shock factor binding protein 1	0.6	4.8E-04	7662.6	1971.7	97	100
ENSMUSG00000046352	Gjb2	gap junction protein, beta 2	-0.6	2.6E-05	463.2	1968.6	92	87
ENSMUSG00000037656	Slc20a2	solute carrier family 20, member 2	-0.6	6.1E-07	533.3	1968.2	85	84
ENSMUSG00000026051	1500015O10	RIKEN cDNA 1500015O10 gene	-1.4	1.8E-08	64.6	1955.6	84	93
ENSMUSG00000020664	Dld	dihydrolipoamide dehydrogenase	0.4	1.5E-02	4565.3	1950.9	92	94
ENSMUSG00000039601	Rcan2	regulator of calcineurin 2	0.4	2.4E-10	4495.9	1946.7	85	81
ENSMUSG00000024491	Rbm27	RNA binding motif protein 27	-0.1	7.5E-01	1689.6	1941.2	86	81
ENSMUSG00000027303	Ptpra	protein tyrosine phosphatase, receptor type, A	-0.4	2.7E-08	833.7	1935.1	86	87
ENSMUSG00000072704	2700089E24	RIKEN cDNA 2700089E24 gene	0.0	8.0E-01	2145.7	1921.6	90	90
ENSMUSG00000024474	Ik	IK cytokine	0.2	8.0E-02	3218.8	1886.9	88	88
ENSMUSG00000021112	Mpp5	membrane protein, palmitoylated 5 (MAGUK p55 subfamily mem	0.2	9.9E-02	2819.1	1865.2	88	85
ENSMUSG00000021981	Cab39l	calcium binding protein 39-like	-0.3	1.4E-01	964.1	1853.8	89	88
ENSMUSG00000034135	Sik3	SIK family kinase 3	0.3	2.6E-03	3753.8	1851.1	82	80
ENSMUSG00000025066	Sfr1	SWI5 dependent recombination repair 1	0.2	2.0E-01	2648.0	1818.4	91	86
ENSMUSG00000031776	Arl2bp	ADP-ribosylation factor-like 2 binding protein	0.1	5.8E-01	2227.4	1809.8	97	85
ENSMUSG00000075470	Alg10b	asparagine-linked glycosylation 10B (alpha-1,2-glucosyltransfera	-1.1	2.1E-19	139.8	1807.8	88	81
ENSMUSG00000027680	Fxr1	fragile X mental retardation gene 1, autosomal homolog	-0.2	3.3E-01	1128.1	1801.2	93	87
ENSMUSG00000016541	Atxn10	ataxin 10	0.3	2.6E-02	3914.5	1795.3	94	84
ENSMUSG00000020390	Ube2b	ubiquitin-conjugating enzyme E2B	0.4	1.1E-04	4157.3	1784.5	82	85
ENSMUSG00000022964	Tmem50b	transmembrane protein 50B	0.1	7.9E-01	2010.3	1781.9	88	85
ENSMUSG00000035248	Zcchc6	zinc finger, CCHC domain containing 6	-0.4	8.9E-06	710.8	1772.0	87	85
ENSMUSG00000022698	Naa50	N(alpha)-acetyltransferase 50, NatE catalytic subunit	0.1	4.8E-01	2249.0	1769.1	90	91
ENSMUSG00000032458	Copb2	coatomer protein complex, subunit beta 2 (beta prime)	-0.1	7.0E-01	1550.2	1761.3	91	86
ENSMUSG00000046240	Hepacam	hepatocyte cell adhesion molecule	0.6	1.9E-05	7424.3	1745.2	85	89
ENSMUSG00000030213	Atf7ip	activating transcription factor 7 interacting protein	-0.5	1.8E-11	491.6	1730.2	91	88
ENSMUSG00000061740	Cyp2d22	cytochrome P450, family 2, subfamily d, polypeptide 22	-0.2	4.7E-02	1053.3	1723.2	83	84
ENSMUSG00000025369	Smarcc2	SWI/SNF related, matrix associated, actin dependent regulator of	0.0	9.4E-01	1660.3	1714.1	81	82
ENSMUSG00000036949	Slc39a12	solute carrier family 39 (zinc transporter), member 12	-0.2	1.3E-01	1047.9	1705.8	84	81
ENSMUSG00000026880	Stom	stomatin	-1.1	4.0E-19	135.7	1696.2	91	89
ENSMUSG00000074748	Atxn7l3b	ataxin 7-like 3B	0.5	9.1E-12	5684.4	1691.3	91	91
ENSMUSG00000026753	Ppp6c	protein phosphatase 6, catalytic subunit	0.3	1.5E-02	3669.2	1688.2	91	88
ENSMUSG00000062604	Srpk2	serine/arginine-rich protein specific kinase 2	0.2	4.5E-02	2799.3	1683.9	87	80
ENSMUSG00000029202	Pds5a	PDS5, regulator of cohesion maintenance, homolog A (S, cerevisi	-0.5	3.7E-04	507.5	1672.4	92	83
ENSMUSG00000037206	Islr	immunoglobulin superfamily containing leucine-rich repeat	-1.1	2.5E-19	124.3	1671.1	82	82
ENSMUSG00000008734	Gprc5b	G protein-coupled receptor, family C, group 5, member B	-0.2	8.6E-02	952.5	1670.7	87	85
ENSMUSG00000063856	Gpx1	glutathione peroxidase 1	0.0	9.6E-01	1634.1	1670.4	82	80
ENSMUSG00000029536	Gatc	glutamyl-tRNA(Gln) amidotransferase, subunit C homolog (bacte	0.2	4.6E-01	2413.5	1663.3	95	92
ENSMUSG00000025823	Pdia4	protein disulfide isomerase associated 4	-0.8	1.1E-09	231.0	1656.2	80	82
ENSMUSG00000005371	Fbxo11	F-box protein 11	0.1	4.5E-01	2058.8	1649.7	85	85
ENSMUSG00000024740	Ddb1	damage specific DNA binding protein 1	0.0	8.5E-01	1542.5	1649.2	88	81
ENSMUSG00000025630	Hprt	hypoxanthine guanine phosphoribosyl transferase	-0.3	1.2E-02	780.3	1644.8	94	94
ENSMUSG00000011884	Gltp	glycolipid transfer protein	0.0	7.7E-01	1783.9	1632.2	93	83
ENSMUSG00000042390	Gatad2b	GATA zinc finger domain containing 2B	-0.1	7.1E-01	1429.9	1622.2	99	94
ENSMUSG00000075701	Vimp	VCP-interacting membrane protein	-0.3	1.4E-01	805.3	1619.7	93	94
ENSMUSG00000024908	Ppp6r3	protein phosphatase 6, regulatory subunit 3	0.0	9.9E-01	1604.0	1615.7	96	89
ENSMUSG00000000149	Gna12	guanine nucleotide binding protein, alpha 12	0.2	1.2E-01	2289.7	1610.0	89	80
ENSMUSG00000032405	Pias1	protein inhibitor of activated STAT 1	0.0	9.4E-01	1562.8	1607.4	94	86
ENSMUSG00000059811	Atl2	atlastin GTPase 2	0.0	9.3E-01	1660.3	1604.9	92	91
ENSMUSG00000020386	Sar1b	SAR1 gene homolog B (S, cerevisiae)	0.1	7.0E-01	1976.4	1586.1	88	87
ENSMUSG00000031765	Mt1	metallothionein 1	1.0	2.0E-22	16844.4	1585.6	99	98
ENSMUSG00000022867	Usp25	ubiquitin specific peptidase 25	-0.3	2.4E-02	801.0	1583.7	93	91
ENSMUSG00000050017	Pitpnb	phosphatidylinositol transfer protein, beta	-0.1	8.2E-01	1375.1	1574.1	93	91
ENSMUSG00000003974	Grm3	glutamate receptor, metabotropic 3	0.0	9.2E-01	1470.8	1567.0	90	84
ENSMUSG00000060992	Copz1	coatomer protein complex, subunit zeta 1	0.4	8.3E-04	3820.2	1563.4	90	87
ENSMUSG00000027206	Cops2	COP9 (constitutive photomorphogenic) homolog, subunit 2 (Arab	0.2	4.4E-02	2599.6	1563.0	89	84
ENSMUSG00000033917	Gde1	glycerophosphodiester phosphodiesterase 1	-0.4	1.3E-05	602.5	1543.9	85	83
ENSMUSG00000005103	Wdr1	WD repeat domain 1	0.1	2.5E-01	2079.2	1542.6	88	84
ENSMUSG00000032212	Sltm	SAFB-like, transcription modulator	-0.4	4.1E-03	565.8	1541.3	92	91
ENSMUSG00000028149	Rap1gds1	RAP1, GTP-GDP dissociation stimulator 1	0.2	1.5E-01	2370.9	1539.0	90	83
ENSMUSG00000003518	Dusp3	dual specificity phosphatase 3 (vaccinia virus phosphatase VH1-re	-0.1	3.5E-01	1260.3	1527.0	84	80
ENSMUSG00000059263	Usp47	ubiquitin specific peptidase 47	0.0	9.0E-01	1567.6	1509.3	86	85
ENSMUSG00000074238	Ap1ar	adaptor-related protein complex 1 associated regulatory protein	-0.2	1.7E-01	939.6	1507.8	85	82
ENSMUSG00000022000	Zc3h13	zinc finger CCCH type containing 13	-0.4	7.9E-04	551.2	1475.6	83	83
ENSMUSG00000028173	Wls	wntless homolog (Drosophila)	-0.7	1.8E-06	269.1	1471.0	89	87
ENSMUSG00000020994	Pnn	pinin	-0.4	2.5E-02	588.4	1446.8	87	85
ENSMUSG00000030774	Pak1	p21 protein (Cdc42/Rac)-activated kinase 1	0.4	1.8E-05	3445.6	1446.6	89	89
ENSMUSG00000059713	Rcan3	regulator of calcineurin 3	-0.1	6.3E-01	1246.3	1442.2	87	81
ENSMUSG00000009035	Tmem184b	transmembrane protein 184b	-0.7	6.8E-07	290.4	1435.0	85	84
ENSMUSG00000022091	Sorbs3	sorbin and SH3 domain containing 3	0.2	8.3E-02	2147.8	1433.3	88	84
ENSMUSG00000044229	Nxpe4	neurexophilin and PC-esterase domain family, member 4	-0.6	2.2E-23	337.1	1425.7	89	89
ENSMUSG00000021537	Cetn3	centrin 3	0.4	6.4E-06	3794.9	1403.7	98	95
ENSMUSG00000021474	Sfxn1	sideroflexin 1	0.4	4.4E-04	3733.4	1395.4	89	87
ENSMUSG00000029649	Pomp	proteasome maturation protein	0.3	3.1E-02	2902.8	1394.7	95	91
ENSMUSG00000029169	Dhx15	DEAH (Asp-Glu-Ala-His) box polypeptide 15	0.1	5.6E-01	1637.4	1373.9	89	87
ENSMUSG00000020467	Efemp1	epidermal growth factor-containing fibulin-like extracellular matri	-1.0	6.1E-17	138.8	1372.4	89	87
ENSMUSG00000029407	Uso1	USO1 vesicle docking factor	0.0	8.5E-01	1296.7	1372.2	88	81
ENSMUSG00000024404	Riok3	RIO kinase 3	0.0	9.1E-01	1449.3	1368.4	82	85
ENSMUSG00000020650	Bcap29	B cell receptor associated protein 29	-0.5	5.9E-03	440.7	1365.3	83	81
ENSMUSG00000031696	Vps35	vacuolar protein sorting 35	0.3	9.9E-02	2610.3	1359.5	94	89
ENSMUSG00000070923	Klhl9	kelch-like 9	0.5	1.5E-06	4176.4	1354.7	91	81
ENSMUSG00000037049	Smpd1	sphingomyelin phosphodiesterase 1, acid lysosomal	-0.4	1.7E-02	543.2	1351.8	89	84
ENSMUSG00000029084	Cd38	CD38 antigen	-0.3	8.3E-02	684.1	1332.8	88	88
ENSMUSG00000027500	Stmn2	stathmin-like 2	-0.1	6.8E-01	1115.1	1325.5	98	97
ENSMUSG00000039704	Lmbrd2	LMBR1 domain containing 2	-0.7	3.5E-07	263.4	1323.6	89	81
ENSMUSG00000047213	Ythdf3	YTH domain family 3	0.1	6.1E-01	1687.1	1320.6	85	85
ENSMUSG00000003420	Fcgrt	Fc receptor, IgG, alpha chain transporter	-0.8	1.0E-15	204.6	1304.9	93	97
ENSMUSG00000031021	Tmem9b	TMEM9 domain family, member B	-0.1	3.2E-01	1067.5	1303.7	87	82
ENSMUSG00000081534	Slc48a1	solute carrier family 48 (heme transporter), member 1	0.0	8.5E-01	1422.7	1301.2	88	85
ENSMUSG00000056851	Pcbp2	poly(rC) binding protein 2	0.0	9.2E-01	1333.5	1299.1	86	84
ENSMUSG00000027363	Usp8	ubiquitin specific peptidase 8	0.2	1.1E-01	2044.0	1297.2	86	86

ENSMUSG00000023921	Mut	methylmalonyl-Coenzyme A mutase	0.2	4.2E-01	2061.0	1294.1	83	85
ENSMUSG00000025544	Tm9sf2	transmembrane 9 superfamily member 2	-0.6	7.6E-11	358.5	1290.8	82	83
ENSMUSG00000028419	Chmp5	charged multivesicular body protein 5	0.5	2.6E-05	4165.8	1290.3	91	81
ENSMUSG00000034463	Scara3	scavenger receptor class A, member 3	-0.2	2.5E-01	784.8	1275.6	82	83
ENSMUSG00000020074	Ccar1	cell division cycle and apoptosis regulator 1	0.0	9.2E-01	1332.8	1272.9	81	84
ENSMUSG00000038546	Ranbp9	RAN binding protein 9	0.3	6.3E-02	2469.2	1268.2	83	84
ENSMUSG00000079111	Kdelr2	KDEL (Lys-Asp-Glu-Leu) endoplasmic reticulum protein retentio	-0.6	1.1E-07	324.8	1261.0	91	91
ENSMUSG00000034152	Exoc3	exocyst complex component 3	0.2	1.8E-01	1907.0	1250.1	87	81
ENSMUSG00000053012	Krcc1	lysine-rich coiled-coil 1	0.1	5.8E-01	1582.2	1244.2	88	87
ENSMUSG00000032348	Gsta4	glutathione S-transferase, alpha 4	0.3	6.1E-03	2553.7	1242.0	93	89
ENSMUSG00000049612	Omg	oligodendrocyte myelin glycoprotein	0.3	7.3E-03	2581.1	1234.2	91	80
ENSMUSG00000026202	Tuba4a	tubulin, alpha 4A	0.6	NA	5131.2	1233.0	99	90
ENSMUSG00000033940	Brk1	BRICK1, SCAR/WAVE actin-nucleating complex subunit	0.7	5.5E-09	5965.3	1224.6	91	85
ENSMUSG00000032060	Cryab	crystallin, alpha B	0.6	8.7E-11	4421.0	1218.8	85	81
ENSMUSG00000021760	Gpx8	glutathione peroxidase 8 (putative)	-0.8	4.5E-07	182.6	1214.5	97	100
ENSMUSG00000028416	Bag1	BCL2-associated athanogene 1	0.3	2.0E-01	2307.4	1212.9	83	81
ENSMUSG00000030754	Copb1	coatomer protein complex, subunit beta 1	-0.2	6.3E-02	684.0	1196.4	95	88
ENSMUSG00000032026	Rexo2	REX2, RNA exonuclease 2 homolog (S, cerevisiae)	0.2	1.3E-02	1912.5	1191.0	91	90
ENSMUSG00000036095	Dgkb	diacylglycerol kinase, beta	0.5	8.0E-05	4113.3	1168.0	85	81
ENSMUSG00000008859	Rala	v-ral simian leukemia viral oncogene homolog A (ras related)	0.3	3.9E-03	2261.5	1161.6	86	85
ENSMUSG00000021238	Aldh6a1	aldehyde dehydrogenase family 6, subfamily A1	0.8	6.8E-06	8419.3	1153.4	85	81
ENSMUSG00000030647	Ndufc2	NADH dehydrogenase (ubiquinone) 1, subcomplex unknown, 2	0.5	1.4E-02	3402.0	1144.4	88	82
ENSMUSG00000001942	Siae	sialic acid acetylesterase	-0.8	2.9E-12	171.5	1141.1	88	84
ENSMUSG00000027428	Rbbp9	retinoblastoma binding protein 9	0.5	4.0E-04	3728.8	1130.3	90	83
ENSMUSG00000028822	Tmem50a	transmembrane protein 50A	-0.3	6.0E-03	555.6	1125.8	80	80
ENSMUSG00000022403	St13	suppression of tumorigenicity 13	0.5	1.5E-14	3484.6	1124.2	87	86
ENSMUSG00000021114	Atp6v1d	ATPase, H+ transporting, lysosomal V1 subunit D	0.4	1.2E-04	2578.7	1111.2	88	84
ENSMUSG00000033047	Eif3l	eukaryotic translation initiation factor 3, subunit L	0.0	8.4E-01	1030.9	1100.1	95	94
ENSMUSG00000027495	Fam210b	family with sequence similarity 210, member B	0.1	7.4E-01	1352.2	1082.4	81	82
ENSMUSG00000020589	Fam49a	family with sequence similarity 49, member A	0.6	4.7E-08	4547.7	1077.6	88	82
ENSMUSG00000004789	Dlst	dihydrolipoamide S-succinyltransferase (E2 component of 2-oxo-g	0.3	4.0E-02	1936.7	1075.1	87	83
ENSMUSG00000000399	Ndufa9	NADH dehydrogenase (ubiquinone) 1 alpha subcomplex, 9	0.4	3.4E-03	2949.8	1071.6	92	81
ENSMUSG00000032011	Thy1	thymus cell antigen 1, theta	0.1	7.4E-01	1201.1	1065.6	84	81
ENSMUSG00000020708	Psmc5	protease (prosome, macropain) 26S subunit, ATPase 5	0.4	3.7E-03	2445.6	1053.0	96	92
ENSMUSG00000031791	Tmem38a	transmembrane protein 38A	-0.3	5.7E-02	507.3	1044.2	80	81
ENSMUSG00000040520	Manea	mannosidase, endo-alpha	-0.1	6.1E-01	784.0	1037.7	82	81
ENSMUSG00000029777	Gars	glycyl-tRNA synthetase	0.0	8.6E-01	944.4	1033.7	87	85
ENSMUSG00000039007	Cpq	carboxypeptidase Q	-0.8	1.5E-08	151.8	1019.2	84	81
ENSMUSG00000025006	Sorbs1	sorbin and SH3 domain containing 1	0.1	4.8E-01	1272.3	1014.8	89	80
ENSMUSG00000030059	Tmf1	TATA element modulatory factor 1	-0.1	5.6E-01	846.4	1013.1	87	81
ENSMUSG00000073639	Rab18	RAB18, member RAS oncogene family	0.7	4.6E-07	5195.0	1001.2	91	80
ENSMUSG00000021701	Plk2	polo-like kinase 2	-0.2	2.4E-01	574.2	991.4	89	88
ENSMUSG00000004394	Tmed4	transmembrane emp24 protein transport domain containing 4	-0.4	4.2E-05	389.1	989.4	83	83
ENSMUSG00000032766	Gng11	guanine nucleotide binding protein (G protein), gamma 11	-0.4	1.7E-02	423.2	974.7	86	82
ENSMUSG00000001774	Chordc1	cysteine and histidine-rich domain (CHORD)-containing, zinc-bin	0.2	2.3E-01	1484.7	969.2	92	83
ENSMUSG00000041645	Ddx24	DEAD (Asp-Glu-Ala-Asp) box polypeptide 24	0.0	8.0E-01	880.0	960.5	81	82
ENSMUSG00000032018	Sc5d	sterol-C5-desaturase (fungal ERG3, delta-5-desaturase) homolog (	-0.3	2.3E-02	468.5	955.4	88	88
ENSMUSG00000020644	Id2	inhibitor of DNA binding 2	1.2	2.4E-19	17351.5	942.1	92	81
ENSMUSG00000032279	Idh3a	isocitrate dehydrogenase 3 (NAD+) alpha	0.6	2.7E-08	3922.2	933.1	91	84
ENSMUSG00000063297	Luzp2	leucine zipper protein 2	0.5	1.4E-02	3006.1	924.0	92	99
ENSMUSG00000029156	Sgcb	sarcoglycan, beta (dystrophin-associated glycoprotein)	-0.3	1.5E-01	492.7	923.5	82	90
ENSMUSG00000006360	Crip1	cysteine-rich protein 1 (intestinal)	-1.0	1.1E-11	84.2	913.8	93	93
ENSMUSG00000028005	Gucy1b3	guanylate cyclase 1, soluble, beta 3	-0.1	5.5E-01	727.2	908.6	85	80
ENSMUSG00000023367	Tmem176a	transmembrane protein 176A	-0.3	1.8E-03	437.3	903.7	90	92
ENSMUSG00000022295	Atp6v1c1	ATPase, H+ transporting, lysosomal V1 subunit C1	0.5	7.9E-03	2928.9	900.2	91	83
ENSMUSG00000062929	Cfl2	cofilin 2, muscle	0.6	2.5E-04	3574.1	899.4	81	81
ENSMUSG00000036295	Lrrn3	leucine rich repeat protein 3, neuronal	-0.7	7.5E-07	180.2	895.7	86	91
ENSMUSG00000066232	Ipo7	importin 7	-0.2	1.6E-01	520.4	890.2	95	81
ENSMUSG00000002015	Bcap31	B cell receptor associated protein 31	-0.5	2.4E-02	296.0	888.9	88	91
ENSMUSG00000016018	Skiv2l2	superkiller viralicidic activity 2-like 2 (S, cerevisiae)	0.0	7.8E-01	804.5	888.3	86	80
ENSMUSG00000096262	Ap1g1	adaptor protein complex AP-1, gamma 1 subunit	-0.2	3.0E-01	606.3	874.8	96	96
ENSMUSG00000072294	Klf12	Kruppel-like factor 12	-0.1	3.3E-01	633.2	871.1	90	84
ENSMUSG00000031954	Cfdp1	craniofacial development protein 1	0.4	1.0E-04	2194.3	866.4	94	84
ENSMUSG00000046818	Ddit4l	DNA-damage-inducible transcript 4-like	0.4	3.8E-03	2331.7	864.6	81	83
ENSMUSG00000094870	Zfp131	zinc finger protein 131	-0.3	1.6E-02	449.3	861.2	93	85
ENSMUSG00000041986	Elmod1	ELMO/CED-12 domain containing 1	-0.2	1.1E-01	560.0	861.1	86	85
ENSMUSG00000039756	Dnttip2	deoxynucleotidyltransferase, terminal, interacting protein 2	-0.1	5.6E-01	697.4	859.7	91	85
ENSMUSG00000033793	Atp6v1h	ATPase, H+ transporting, lysosomal V1 subunit H	0.3	2.0E-02	1904.2	859.5	90	81
ENSMUSG00000039067	Psmd7	proteasome (prosome, macropain) 26S subunit, non-ATPase, 7	0.4	8.7E-04	2252.5	848.5	91	87
ENSMUSG00000025381	Cnpy2	canopy 2 homolog (zebrafish)	-0.3	6.8E-02	411.2	845.0	84	80
ENSMUSG00000059970	Hspa2	heat shock protein 2	0.5	1.6E-03	2894.8	843.2	80	89
ENSMUSG00000022404	Slc25a17	solute carrier family 25 (mitochondrial carrier, peroxisomal memb	0.0	9.1E-01	777.8	842.6	94	80
ENSMUSG00000071180	Smim15	small integral membrane protein 15	0.4	1.1E-02	2105.0	840.1	98	95
ENSMUSG00000029048	Rer1	RER1 retention in endoplasmic reticulum 1 homolog (S, cerevisia	0.0	8.8E-01	782.4	830.7	82	89
ENSMUSG00000068882	Ssb	Sjogren syndrome antigen B	0.3	1.7E-02	1604.6	825.1	91	85
ENSMUSG00000021823	Vcl	vinculin	-0.3	2.0E-01	398.7	823.3	87	88
ENSMUSG00000031948	Kars	lysyl-tRNA synthetase	-0.1	3.8E-01	679.4	813.2	91	83
ENSMUSG00000022766	Serpind1	serine (or cysteine) peptidase inhibitor, clade D, member 1	-1.1	5.3E-09	66.7	812.9	84	85
ENSMUSG00000040385	Ppp1ca	protein phosphatase 1, catalytic subunit, alpha isoform	0.4	2.1E-03	2148.9	808.0	95	90
ENSMUSG00000019210	Atp6v1e1	ATPase, H+ transporting, lysosomal V1 subunit E1	0.6	1.2E-11	3197.4	806.9	93	89
ENSMUSG00000042229	Rabif	RAB interacting factor	0.1	7.3E-01	978.8	805.0	90	83
ENSMUSG00000047036	Zfp445	zinc finger protein 445	-0.3	3.3E-02	381.0	780.8	87	84
ENSMUSG00000004096	Cwc15	CWC15 homolog (S, cerevisiae)	0.5	1.3E-06	2312.9	776.1	93	81
ENSMUSG00000036880	Acaa2	acetyl-Coenzyme A acyltransferase 2 (mitochondrial 3-oxoacyl-C	0.5	1.5E-06	2485.5	772.4	87	92
ENSMUSG00000026020	Nop58	NOP58 ribonucleoprotein	-0.3	1.5E-03	388.0	768.4	91	86
ENSMUSG00000070697	Utp3	UTP3, small subunit (SSU) processome component, homolog (S, c	-0.1	3.9E-01	581.7	758.4	93	94
ENSMUSG00000044080	S100a1	S100 calcium binding protein A1	1.0	4.8E-07	7640.1	753.9	92	83
ENSMUSG00000019173	Rab5c	RAB5C, member RAS oncogene family	0.6	3.7E-06	2904.9	751.2	80	81
ENSMUSG00000022354	Ndufb9	NADH dehydrogenase (ubiquinone) 1 beta subcomplex, 9	0.5	1.0E-08	2678.7	750.5	94	90
ENSMUSG00000068617	Efcab1	EF hand calcium binding domain 1	-0.8	2.0E-05	123.5	744.5	86	81
ENSMUSG00000024870	Rab1b	RAB1B, member RAS oncogene family	0.4	5.5E-03	1928.4	721.7	85	88
ENSMUSG00000041084	Ostc	oligosaccharyltransferase complex subunit	0.0	7.6E-01	643.4	716.9	91	90
ENSMUSG00000031729	Ist1	increased sodium tolerance 1 homolog (yeast)	0.3	1.7E-01	1305.7	708.7	84	81
ENSMUSG00000053565	Eif3k	eukaryotic translation initiation factor 3, subunit K	0.2	2.7E-01	1016.2	704.9	100	99
ENSMUSG00000003380	Rabac1	Rab acceptor 1 (prenylated)	0.0	7.3E-01	641.9	704.8	88	89

ENSMUSG00000024097	Srsf7	serine/arginine-rich splicing factor 7	0.3	1.2E-01	1582.8	704.3	85	80
ENSMUSG00000032606	Nicn1	nicolin 1	0.4	6.5E-06	1856.8	700.5	91	88
ENSMUSG00000070319	Eif3g	eukaryotic translation initiation factor 3, subunit G	0.2	5.2E-02	1200.8	690.5	94	86
ENSMUSG00000042082	Arsb	arylsulfatase B	-0.5	4.5E-03	215.9	672.2	87	93
ENSMUSG00000030317	Timp4	tissue inhibitor of metalloproteinase 4	0.9	5.4E-05	5744.6	671.5	83	81
ENSMUSG00000022551	Cyc1	cytochrome c-1	0.8	2.3E-07	4221.9	662.1	90	80
ENSMUSG00000070934	Rraga	Ras-related GTP binding A	0.3	9.1E-03	1434.8	661.9	90	81
ENSMUSG00000028843	Sh3bgrl3	SH3 domain binding glutamic acid-rich protein-like 3	0.0	9.3E-01	625.6	655.5	90	85
ENSMUSG00000071655	Ubxn1	UBX domain protein 1	0.3	3.6E-02	1303.3	642.6	95	87
ENSMUSG00000026245	Farsb	phenylalanyl-tRNA synthetase, beta subunit	0.1	3.5E-01	855.1	638.9	90	80
ENSMUSG00000030847	Bag3	BCL2-associated athanogene 3	0.1	6.2E-01	849.2	635.2	80	84
ENSMUSG00000022490	Ppp1r1a	protein phosphatase 1, regulatory (inhibitor) subunit 1A	0.0	8.0E-01	561.6	617.2	84	92
ENSMUSG00000014769	Psmb1	proteasome (prosome, macropain) subunit, beta type 1	0.4	1.5E-02	1495.4	613.0	93	91
ENSMUSG00000014294	Ndufa2	NADH dehydrogenase (ubiquinone) 1 alpha subcomplex, 2	0.3	1.4E-01	1266.4	611.9	97	95
ENSMUSG00000029632	Ndufa4	NADH dehydrogenase (ubiquinone) 1 alpha subcomplex, 4	0.5	5.2E-03	1962.2	609.1	96	98
ENSMUSG00000022453	Naga	N-acetyl galactosaminidase, alpha	-0.3	1.5E-04	306.8	591.8	87	82
ENSMUSG00000016528	Mapkapk2	MAP kinase-activated protein kinase 2	0.1	5.1E-01	704.5	589.4	90	81
ENSMUSG00000021832	Psmc6	proteasome (prosome, macropain) 26S subunit, ATPase, 6	0.2	1.1E-01	1002.4	584.2	90	86
ENSMUSG00000041126	H2afv	H2A histone family, member V	0.6	4.3E-05	2405.6	582.1	90	88
ENSMUSG00000048970	C1galt1c1	C1GALT1-specific chaperone 1	-0.2	3.9E-01	333.8	580.6	81	90
ENSMUSG00000025868	Higd2a	HIG1 domain family, member 2A	0.6	7.2E-07	2208.0	578.1	94	94
ENSMUSG00000024780	Cdc37l1	cell division cycle 37-like 1	0.6	1.9E-12	2305.7	571.3	92	81
ENSMUSG00000024997	Prdx3	peroxiredoxin 3	0.6	2.2E-05	2399.7	557.3	87	87
ENSMUSG00000055720	Ubl7	ubiquitin-like 7 (bone marrow stromal cell-derived)	0.3	1.1E-02	1171.8	551.6	89	80
ENSMUSG00000004902	Slc25a18	solute carrier family 25 (mitochondrial carrier), member 18	1.0	3.2E-04	6134.8	538.1	83	94
ENSMUSG00000019789	Hey2	hairy/enhancer-of-split related with YRPW motif 2	0.1	4.3E-01	719.5	532.6	80	85
ENSMUSG00000010376	Nedd8	neural precursor cell expressed, developmentally down-regulated	0.6	5.2E-07	2057.6	526.4	99	85
ENSMUSG00000035765	Dym	dymeclin	0.1	5.0E-01	693.7	507.9	88	82
ENSMUSG00000006315	Tmem147	transmembrane protein 147	-0.4	1.5E-05	197.8	505.8	89	82
ENSMUSG00000036570	Fxyd1	FXYD domain-containing ion transport regulator 1	0.1	7.7E-01	578.8	504.1	86	89
ENSMUSG00000009281	Rarres2	retinoic acid receptor responder (tazarotene induced) 2	-0.9	5.9E-11	55.7	501.5	99	99
ENSMUSG00000025651	Uqcrc1	ubiquinol-cytochrome c reductase core protein 1	0.5	4.2E-06	1523.7	496.6	87	83
ENSMUSG00000051256	Jagn1	jagunal homolog 1 (Drosophila)	-0.1	5.9E-01	400.8	484.3	83	83
ENSMUSG00000062381	Vps28	vacuolar protein sorting 28 (yeast)	0.6	3.4E-11	1715.1	474.1	92	89
ENSMUSG00000021033	Gstz1	glutathione transferase zeta 1 (maleylacetoacetate isomerase)	0.6	1.2E-10	1760.0	472.1	86	85
ENSMUSG00000002580	Mien1	migration and invasion enhancer 1	0.6	NA	1779.7	470.4	85	84
ENSMUSG00000024194	Cuta	cutA divalent cation tolerance homolog (E, coli)	0.4	3.8E-02	1234.8	466.3	94	85
ENSMUSG00000030629	Zfand6	zinc finger, AN1-type domain 6	0.4	2.3E-03	1234.9	463.9	88	81
ENSMUSG00000039168	Dap	death-associated protein	-0.2	2.1E-01	295.4	463.9	85	82
ENSMUSG00000006736	Tspan31	tetraspanin 31	-0.3	2.1E-01	219.7	460.4	92	94
ENSMUSG00000022450	Ndufa6	NADH dehydrogenase (ubiquinone) 1 alpha subcomplex, 6 (B14)	0.3	3.5E-02	960.0	447.4	87	84
ENSMUSG00000040759	Cmtm5	CKLF-like MARVEL transmembrane domain containing 5	0.8	5.5E-05	2879.2	440.7	90	99
ENSMUSG00000021290	2010107E04	RIKEN cDNA 2010107E04 gene	0.8	6.9E-18	3000.0	435.1	93	95
ENSMUSG00000039197	Adk	adenosine kinase	1.1	4.1E-08	5365.6	421.7	82	85
ENSMUSG00000063200	Nol7	nucleolar protein 7	0.4	8.6E-03	938.4	413.3	80	81
ENSMUSG00000031578	Mak16	MAK16 homolog (S, cerevisiae)	0.1	4.2E-01	507.3	409.7	87	81
ENSMUSG00000026895	Ndufa8	NADH dehydrogenase (ubiquinone) 1 alpha subcomplex, 8	0.7	1.9E-06	2003.8	405.3	81	85
ENSMUSG00000036751	Cox6b1	cytochrome c oxidase, subunit VIb polypeptide 1	0.7	1.4E-08	2201.7	402.3	93	83
ENSMUSG00000059534	Uqcr10	ubiquinol-cytochrome c reductase, complex III subunit X	0.7	1.3E-09	1796.6	397.0	88	80
ENSMUSG00000025035	Arl3	ADP-ribosylation factor-like 3	0.6	4.4E-09	1666.7	396.7	97	90
ENSMUSG00000059734	Ndufs8	NADH dehydrogenase (ubiquinone) Fe-S protein 8	0.6	9.9E-04	1654.3	394.5	86	85
ENSMUSG00000021607	Mrpl36	mitochondrial ribosomal protein L36	0.5	1.1E-06	1287.8	378.9	91	88
ENSMUSG00000024537	Psmg2	proteasome (prosome, macropain) assembly chaperone 2	0.0	9.8E-01	366.6	364.7	95	80
ENSMUSG00000005054	Cstb	cystatin B	0.5	4.0E-03	1249.2	357.4	91	92
ENSMUSG00000028029	Aimp1	aminoacyl tRNA synthetase complex-interacting multifunctional	0.0	8.9E-01	379.7	352.6	90	82
ENSMUSG00000033916	Chmp2a	charged multivesicular body protein 2A	0.4	2.2E-04	777.4	343.2	89	80
ENSMUSG00000003955	Fam162a	family with sequence similarity 162, member A	0.6	2.6E-03	1485.3	335.9	94	94
ENSMUSG00000019362	D8Ertd738e	DNA segment, Chr 8, ERATO Doi 738, expressed	0.6	4.0E-04	1302.5	331.0	84	81
ENSMUSG00000039016	Timm8b	translocase of inner mitochondrial membrane 8B	0.6	1.7E-09	1283.4	330.5	96	96
ENSMUSG00000062054	Iah1	isoamyl acetate-hydrolyzing esterase 1 homolog (S, cerevisiae)	-0.3	2.2E-01	151.1	322.3	85	86
ENSMUSG00000071662	Polr2g	polymerase (RNA) II (DNA directed) polypeptide G	0.5	6.1E-03	986.3	322.3	91	84
ENSMUSG00000027667	Zfp639	zinc finger protein 639	0.1	5.6E-01	406.9	296.3	88	81
ENSMUSG00000029864	Gstk1	glutathione S-transferase kappa 1	0.6	2.9E-05	1058.2	294.5	93	84
ENSMUSG00000057411	Fam173a	family with sequence similarity 173, member A	0.2	4.4E-01	432.3	291.5	94	86
ENSMUSG00000000088	Cox5a	cytochrome c oxidase subunit Va	0.7	2.2E-42	1525.7	281.0	91	90
ENSMUSG00000074754	Gm561	predicted gene 561	0.2	4.9E-01	420.8	280.0	98	94
ENSMUSG00000033429	Mcee	methylmalonyl CoA epimerase	0.8	2.6E-05	1550.1	255.8	80	87
ENSMUSG00000016427	Ndufa1	NADH dehydrogenase (ubiquinone) 1 alpha subcomplex, 1	0.2	3.5E-01	379.0	254.1	95	95
ENSMUSG00000001445	Mrpl10	mitochondrial ribosomal protein L10	0.3	6.1E-02	549.3	250.9	86	86
ENSMUSG00000039887	Alg14	asparagine-linked glycosylation 14	0.1	7.6E-01	307.4	244.2	85	82
ENSMUSG00000041571	Sepw1	selenoprotein W, muscle 1	1.0	2.2E-11	2527.7	234.9	84	82
ENSMUSG00000067713	Prkag1	protein kinase, AMP-activated, gamma 1 non-catalytic subunit	0.4	2.0E-02	604.3	232.2	81	80
ENSMUSG00000010406	Mrpl52	mitochondrial ribosomal protein L52	0.2	4.5E-01	330.4	230.2	97	99
ENSMUSG00000020949	Fkbp3	FK506 binding protein 3	0.6	2.4E-03	862.3	225.6	93	95
ENSMUSG00000078348	Sf3b5	splicing factor 3b, subunit 5	0.6	1.2E-06	838.6	216.6	91	93
ENSMUSG00000032526	Deb1	differentially expressed in B16F10 1	0.7	1.9E-10	881.9	186.6	95	89
ENSMUSG00000079437	Tmem179b	transmembrane protein 179B	-0.2	3.8E-01	121.8	185.3	85	88
ENSMUSG00000041736	Tspo	translocator protein	-0.3	5.9E-02	89.6	168.0	82	83
ENSMUSG00000053453	Thoc7	THO complex 7 homolog (Drosophila)	0.6	5.1E-03	652.8	147.6	81	87
ENSMUSG00000063787	Chchd1	coiled-coil-helix-coiled-coil-helix domain containing 1	0.5	1.7E-05	376.5	122.8	81	83
ENSMUSG00000034880	Mrpl34	mitochondrial ribosomal protein L34	0.8	6.0E-21	720.3	118.7	82	88
ENSMUSG00000035443	Thyn1	thymocyte nuclear protein 1	0.4	2.3E-01	335.1	113.6	95	87
ENSMUSG00000035048	Anapc13	anaphase promoting complex subunit 13	0.5	2.4E-03	331.3	100.7	82	82
