# Supplementary material for: Translation in astrocyte distal processes sets molecular heterogeneity at the gliovascular interface
Source: Cell Discov. 2017 Mar 28;3:17005–. doi: 10.1038/celldisc.2017.5 (PMC5368712; doi:10.1038/celldisc.2017.5)
Supplement: Supplementary Table S4 [file celldisc20175-s6.pdf]

Table S4 Gene Ontology analysis of the astrocyte endfeet ribosome-bound transcriptome.

The first 25 most enriched “cellular component” and “biological process” GO pathways in the endfeet compared to the astrocyte TRAP transcriptomes are grouped by cellular compartment (extracellular, membrane and intracellular organelles) and biological functions (extracellular matrix, immunity, transport, vascular functions). Number of entities, the number of transcripts referenced in the pathway; Number of measured entities, the number of transcripts found in the TRAP transcriptomes; Median FC, the median change of expression between the astrocyte and endfeet TRAP transcriptomes. A positive score indicates an enrichment of the pathway in the endfeet.

Term type	GO Term	Number of entities	Number of measured entities	Median FC	p-value	Category
Cellular component	extracellular region	2319	1357	1.3	3.4E-47	Extracellular
	extracellular matrix	250	216	1.9	2.5E-35	
	proteinaceous extracellular matrix	318	259	1.8	2.7E-35	
	extracellular space	1166	718	1.4	7.1E-35	
	cell surface	508	418	1.5	6.4E-21	
	basement membrane	108	86	2.3	2.0E-19	
	collagen	98	74	2.3	1.4E-17	
	basal lamina	20	16	2.5	4.0E-07	
	laminin-1 complex	7	7	3.3	1.0E-05	
	integral component of membrane	7193	3863	1.3	7.4E-32	Membrane
	integral component of plasma membrane	1292	926	1.4	4.7E-29	
	external side of plasma membrane	292	199	1.9	8.5E-26	
	plasma membrane	5701	3124	1.2	9.4E-22	
	membrane	7556	5362	1.16	4.40E-17	
	integrin complex	30	27	2.3	2.0E-09	
	receptor complex	123	102	1.5	1.6E-08	
	apical plasma membrane	301	248	1.3	7.2E-08	
	basolateral plasma membrane	185	157	1.4	1.0E-07	
	membrane raft	210	172	1.3	2.9E-06	
	endoplasmic reticulum lumen	187	155	1.6	3.6E-17	Intracellular organelles
	platelet alpha granule lumen	48	39	1.9	1.6E-07	
	extracellular vesicular exosome	83	70	1.6	2.9E-07	
	lysosome	307	258	1.3	3.2E-07	
	lysosomal lumen	73	67	1.6	2.6E-06	
	Golgi lumen	88	62	1.6	9.9E-06	

Term type	GO Term	Number of entities	Number of measured entities	Median FC	p-value	Category
Biological process	extracellular matrix organization	290	239	1.87	1.31E-38	Extracellular matrix/cell adhesion
	cell adhesion	658	542	1.38	1.18E-22	
	extracellular matrix disassembly	82	63	2.19	2.93E-18	
	collagen catabolic process	74	60	2.02	3.14E-17	
	cell-cell adhesion	144	125	1.46	8.47E-10	
	glycosaminoglycan metabolic process	115	107	1.58	1.02E-09	
	cell-matrix adhesion	87	79	1.76	1.18E-09	
	collagen fibril organization	45	37	1.99	4.03E-09	
	positive regulation of cell migration	158	137	1.49	1.02E-08	
	immune response	449	260	1.49	1.29E-18	Immunity
	immune system process	308	270	1.50	1.85E-16	
	inflammatory response	367	283	1.38	2.42E-14	
	wound healing	121	92	1.69	2.53E-14	
	regulation of immune response	114	63	1.90	3.99E-12	
	response to lipopolysaccharide	228	186	1.30	6.67E-12	
	leukocyte migration	115	99	1.71	3.19E-09	
	defense response to bacterium	226	67	1.54	3.86E-08	
	aging	211	183	1.20	4.46E-08	
	transmembrane transport	717	595	1.29	3.60E-09	Transport
	ion transport	650	552	1.26	9.90E-09	
	angiogenesis	249	208	1.41	1.09E-14	Vascular functions
	positive regulation of angiogenesis	114	87	1.65	3.14E-13	
	blood coagulation	482	398	1.30	4.67E-11	
	platelet degranulation	81	68	1.67	5.12E-10	
	response to hypoxia	251	220	1.21	2.41E-09	
