# Supplementary material for: Translation in astrocyte distal processes sets molecular heterogeneity at the gliovascular interface
Source: Cell Discov. 2017 Mar 28;3:17005–. doi: 10.1038/celldisc.2017.5 (PMC5368712; doi:10.1038/celldisc.2017.5)
Supplement: Supplementary Table S5 [file celldisc20175-s7.pdf]

	Accession No	Gene name	Catalog number Advanced Cell Diagnostics	Target Region	Channel
FISH probes	NM_009700.2	Aqp4-C2	417161-C2	1646 - 2644	C2
	NM_007428.3	Agt	426941	347 - 1538	C1
	NM_009655.2	Alcam	462061	501 - 1441	C1
	NM_021704.3	Cxcl12α	460921	431 - 1496	C1
	EF191515	dapB	310043	414 - 862	C1
	NM_001010937.2	Gjb6	458811	251 - 1383	C1
	NM_134438.3	Gpr37l1	319301	251 - 1388	C1
	NM_175189.4	Hepacam	476101	166 - 1082	C1
	NM_008397.4	Itga6	441701	895 - 1860	C1
	NM_009022.4	Lpar1	318591	188 - 1315	C1
	NM_010865.3	Myoc	460981	102 - 1068	C1
	NM_010865.3	Myoc	460981	102 - 1068	C1
	NM_001081306.1	Ptprz1	460991	1374 - 2358	C1
	NM_054055.2	Slc13a3	461041	149 - 1111	C1

	Gene name	Species	company	Dilution Immunofluorescence	Western Blot
Antibodies	Acta2	Mouse monoclonal (clone 1A4)	Sigma	1/500	1/500
	Aqp4	Rabbit polyclonal	Sigma	1/400	1/500
	Cldn5	Rabbit polyclonal	Thermo fisher	/	1/500
	Cx26	Rabbit polyclonal	Thermo fisher	1/500	1/500
	Cx43	Mouse monoclonal	BD Bioscience	1/500	1/500
	GFAP	Chicken polyclonal	Abcam	1/500	/
	Glt 1	Rabbit polyclonal	Frontier institute	1/1000	1/10000
	H3	Rabbit polyclonal	Cell signaling	/	1/2000
	Hepacam/Glialcam	Rabbit polyclonal	Raul Estevez	1/500	/
	Kir4.1	Rabbit polyclonal	Alomone Labs	1/500	1/500

Table S5 List of antibodies and FISH probes
